# Safety and immunogenicity of a ferritin nanoparticle H2 influenza vaccine in healthy adults: a phase 1 trial

**DOI:** 10.1038/s41591-021-01660-8

**Published:** 2022-02-03

**Authors:** Katherine V. Houser, Grace L. Chen, Cristina Carter, Michelle C. Crank, Thuy A. Nguyen, Maria Claudia Burgos Florez, Nina M. Berkowitz, Floreliz Mendoza, Cynthia Starr Hendel, Ingelise J. Gordon, Emily E. Coates, Sandra Vazquez, Judy Stein, Christopher L. Case, Heather Lawlor, Kevin Carlton, Martin R. Gaudinski, Larisa Strom, Amelia R. Hofstetter, C. Jason Liang, Sandeep Narpala, Christian Hatcher, Rebecca A. Gillespie, Adrian Creanga, Masaru Kanekiyo, Julie E. Raab, Sarah F. Andrews, Yi Zhang, Eun Sung Yang, Lingshu Wang, Kwanyee Leung, Wing-Pui Kong, Alec W. Freyn, Raffael Nachbagauer, Peter Palese, Robert T. Bailer, Adrian B. McDermott, Richard A. Koup, Jason G. Gall, Frank Arnold, John R. Mascola, Barney S. Graham, Julie E. Ledgerwood, the VRC 316 Study Team

**Affiliations:** 1 Vaccine Research Center, National Institute of Allergy and Infectious Diseases, National Institutes of Health, Bethesda, MD, USA.; 2 Vaccine Clinical Materials Program, Leidos Biomedical Research, Inc., Frederick National Laboratory for Cancer Research, Frederick, MD, USA.; 3 Commissioned Corps, U.S. Public Health Service, Rockville, MD, USA.; 4 Biostatistics Research Branch, Division of Clinical Research, National Institute of Allergy and Infectious Diseases, National Institutes of Health, Bethesda, MD, USA.; 5 Department of Microbiology, Icahn School of Medicine at Mount Sinai, New York, NY, USA.; 6 Department of Medicine, Icahn School of Medicine at Mount Sinai, New York, NY, USA.; 7 These authors contributed equally: Katherine V. Houser, Grace L. Chen.

## Abstract

Currently, licensed seasonal influenza vaccines display variable vaccine effectiveness, and there remains a need for novel vaccine platforms capable of inducing broader responses against viral protein domains conserved among influenza subtypes. We conducted a first-in-human, randomized, open-label, phase 1 clinical trial (NCT03186781) to evaluate a novel ferritin (H2HA-Ferritin) nanoparticle influenza vaccine platform. The H2 subtype has not circulated in humans since 1968. Adults born after 1968 have been exposed to only the H1 subtype of group 1 influenza viruses, which shares a conserved stem with H2. Including both H2-naive and H2-exposed adults in the trial allowed us to evaluate memory responses against the conserved stem domain in the presence or absence of pre-existing responses against the immunodominant HA head domain. Fifty healthy participants 18–70 years of age received H2HA-Ferritin intramuscularly as a single 20-μg dose (*n* = 5) or a 60-μg dose either twice in a homologous (*n* = 25) prime-boost regimen or once in a heterologous (*n* = 20) prime-boost regimen after a matched H2 DNA vaccine prime. The primary objective of this trial was to evaluate the safety and tolerability of H2HA-Ferritin either alone or in prime-boost regimens. The secondary objective was to evaluate antibody responses after vaccination. Both vaccines were safe and well tolerated, with the most common solicited symptom being mild headache after both H2HA-Ferritin (*n* = 15, 22%) and H2 DNA (*n* = 5, 25%). Exploratory analyses identified neutralizing antibody responses elicited by the H2HA-Ferritin vaccine in both H2-naive and H2-exposed populations. Furthermore, broadly neutralizing antibody responses against group 1 influenza viruses, including both seasonal H1 and avian H5 subtypes, were induced in the H2-naive population through targeting the HA stem. This ferritin nanoparticle vaccine technology represents a novel, safe and immunogenic platform with potential application for pandemic preparedness and universal influenza vaccine development.

Seasonal influenza epidemics and the threat of pandemic influenza outbreaks are perennial global public health challenges. In an average year, seasonal influenza epidemics cause 3–5 million cases of severe illness and 300,000–650,000 deaths worldwide^1,2^, with pandemics capable of much greater morbidity and mortality^3^. Vaccination is an essential tool for seasonal influenza control; however, currently licensed influenza vaccines require annual reformulation and immunization due to their specificity for the variable circulating strains^4^. Influenza viruses are diverse, with many antigenically unique strains of each seasonal subtype circulating simultaneously in the human population. Even when the circulating viruses are well matched to the strains within the vaccine, the effectiveness of seasonal vaccines typically ranges from 40% to 60%, likely due to the lack of adjuvants in the vaccines and the pre-existing immunity to the seasonal subtypes in the population^1^.

Influenza A hemagglutinin (HA) glycoproteins, which mediate binding and entry of host cells, are phylogenetically categorized into two groups: group 1 (for example, H1, H2, H5, H6 and H9) and group 2 (for example, H3, H7 and H10). The H1 and H3 subtypes are currently responsible for the seasonal epidemics, whereas several other group 1 and group 2 subtypes display pandemic potential^5^. Currently licensed seasonal influenza vaccines predominantly elicit antibody responses directed toward the immunodominant globular head domain of HA^6^. The HA head is highly prone to mutations, and vaccine responses against this domain are generally strain specific. However, substantive evidence has emerged that eliciting antibodies against the highly conserved HA stem domain would yield a universal influenza vaccine capable of broad protection within and across group 1 and group 2 viruses, including subtypes with pandemic potential^7–10^. Promising technologies are being developed to predominantly target the HA stem domain to overcome the current limitations of seasonal influenza vaccines, with the goal of developing an improved influenza vaccine^11–13^.

An ideal vaccine design strategy would result in an influenza vaccine platform that is also capable of being rapidly implemented during an emerging pandemic. To address this need, we developed a ferritin nanoparticle-based vaccine platform^11^. Ferritin is a ubiquitous iron storage protein capable of self-assembling into octahedral particles. The presence of three-fold axes on the particle surface allows for the orderly display of trimeric viral antigens, including influenza HA glycoproteins^11^. Because the ferritin subunits self-assemble, the ferritin nanoparticle-based vaccine can be manufactured recombinantly without relying on virus propagation in embryonated eggs. This allows for expedited production time-lines, which are a considerable benefit during a pandemic response. In preclinical studies, this platform was safe and immunogenic and elicited neutralizing antibodies to conserved antigenic sites in both the HA head and stem^11^.

In this phase 1 clinical trial, we evaluated an influenza HA ferritin (H2HA-Ferritin) vaccine in healthy adults between 18 and 70 years of age. The H2HA-Ferritin vaccine is composed of the ectodomain of the HA from the H2N2 pandemic strain A/Singapore/1/57 genetically fused to the ferritin subunit derived from *Helicobacter pylori*^11^. The assembled vaccine displays eight antigenically intact influenza H2 HA trimers in an orderly array on the nanoparticle surface. The H2N2 subtype (comprising H2 HA and N2 neuraminidase) emerged as a pandemic virus in 1957 and circulated in humans until 1968 (ref.^14^). H2 influenza viruses persist in avian reservoirs and, therefore, retain strong pandemic potential^14,15^. Since the H2N2 subtype stopped circulating in humans in 1968, both immunologically naive and experienced individuals exist in the present population. This presented a unique opportunity to evaluate a novel vaccine platform in two distinct exposure populations within the same trial. This first-in-human, dose-escalation, phase 1 clinical trial was designed to assess the safety, tolerability and vaccine-induced antibody response of two doses of H2HA-Ferritin in both H2-naive adults (born after 1969, after H2 influenza stopped circulating in humans) and H2-exposed adults (born before 1966, while H2 influenza circulated). We excluded individuals born between 1966 and 1969 in an attempt to clearly separate the potential historical exposure to H2N2 influenza in the enrolled groups. We also evaluated the utility of this vaccine platform in both homologous and heterologous prime-boost regimens by including a matched H2 HA DNA prime in a subset of participants. Inclusion of a DNA prime in previous phase 1 influenza trials showed that DNA priming can lead to improved heterologous responses^16^. The breadth and potency of vaccine-induced antibody responses were assessed up to 40 weeks after vaccination.

## Results

### Clinical trial design and participants.

**Extended Data Fig. 1 |:**
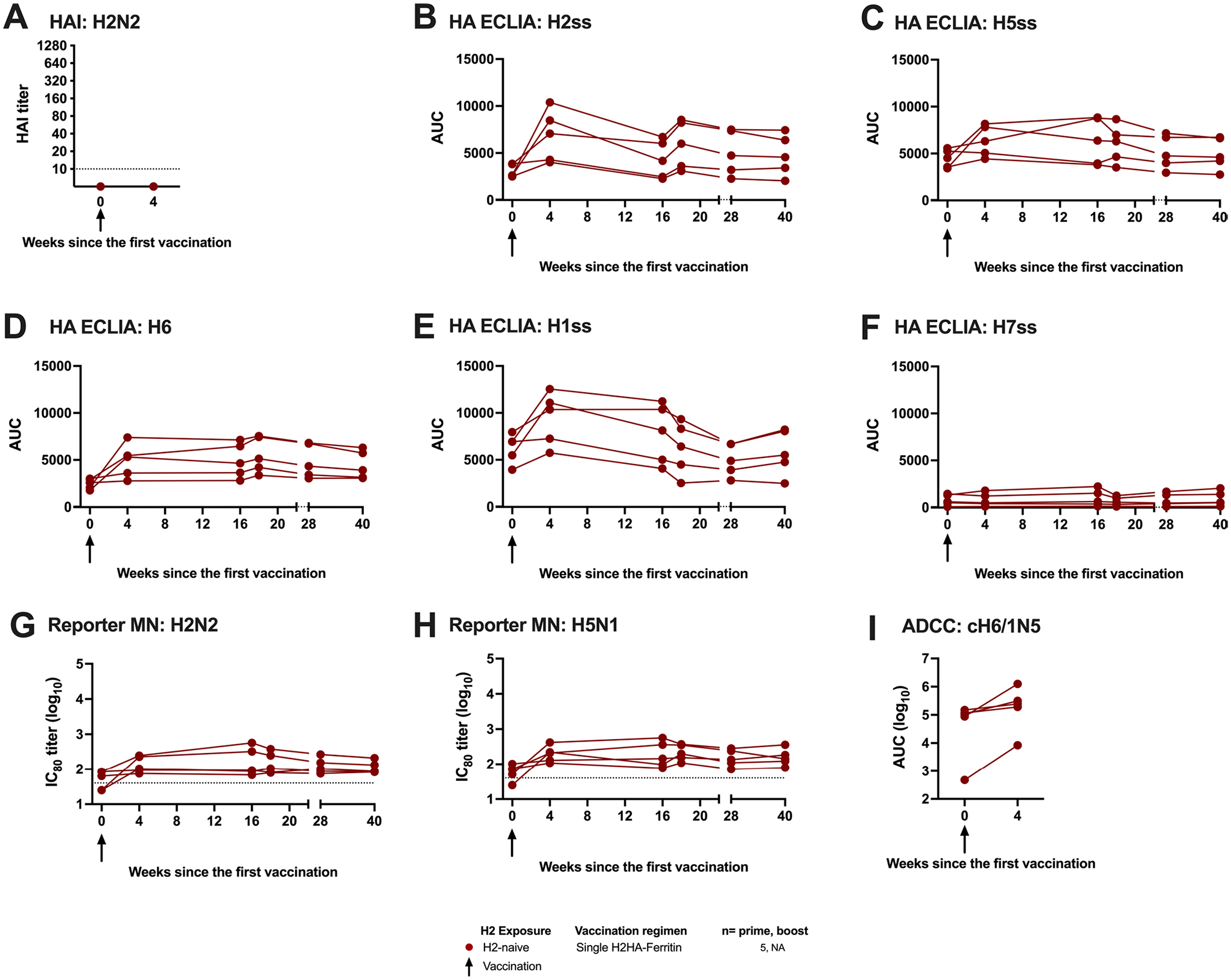
Summary of 20 μg H2HA-Ferritin immunogenicity results. Sera from H2-naïve participants who received a single dose of H2HA-Ferritin (red circles) were analyzed for vaccine-induced antibody responses. Individual participant results are displayed for (**a**) hemagglutination inhibition assay (HAI), (**b**-**f**) HA stem-binding ECLIAs, (**g,h**) reporter microneutralization assays, and (I) Fc-mediated antibody-dependent cell-mediated cytotoxicity ADCC reporter assays. n = 5 for each assay. Dotted lines indicate the lower limit of detection for each assay, arrows indicate vaccination timepoints. Negative samples were reported and calculated as half the limit of detection.

**Fig. 1 |:**
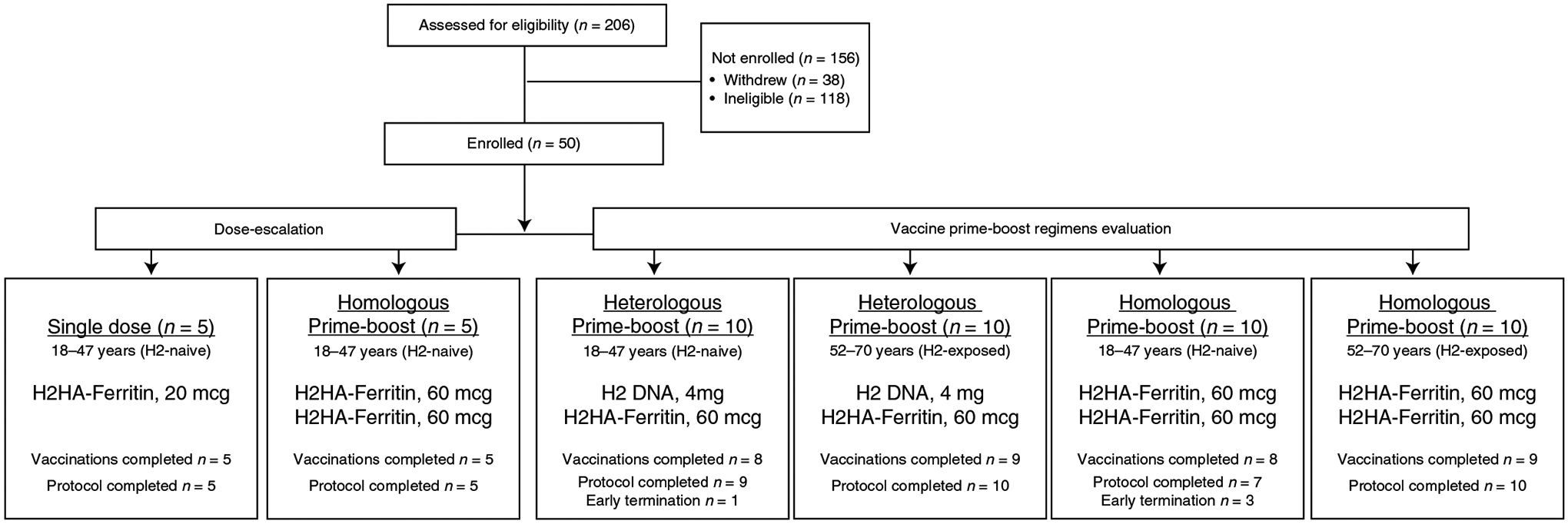
CONSORT diagram for the clinical trial. Participants 18–47 years of age (born after 1969) were considered ‘H2-naive’, and those 52–70 years of age (born before 1966) were considered ‘H2-exposed’, based on potential historical exposure to H2N2 influenza. The interval between prime and boost vaccinations was 16 weeks. Participants who had altered or discontinued vaccination schedules were monitored for safety and were included in the immunogenicity analysis until their vaccination schedules were changed.

To evaluate the safety and vaccine-induced antibody response after H2HA-Ferritin administration, we vaccinated both H2-naive and H2-exposed adults in either homologous or heterologous prime-boost regimens (Fig. 1). For the homologous prime-boost regimen, participants received two 60-μg doses of H2HA-Ferritin approximately 16 weeks apart. In the heterologous prime-boost regimen, participants received a 4-mg H2 DNA prime encoding the matched full-length H2 HA protein administered 16 weeks before vaccination with 60 μg of H2HA-Ferritin. A subset of H2-naive participants received a single dose of 20-μg H2HA-Ferritin in the dose-escalation portion of the study. These 20-μg-dose participants provided valuable safety data for the vaccine platform but were not the focus of the immunological analyses, and, therefore, the results from those participants are summarized in Extended Data Fig. 1.

Fifty healthy adults were enrolled into the trial between 25 October 2017 and 26 November 2018. The final study visit occurred on 3 September 2019. The 25 (50%) male and 25 (50%) female participants had median ages of 34 years (H2-naive adults) and 59 years (H2-exposed adults) (Supplementary Table 1). Most participants (82%) had received at least three seasonal influenza vaccines in the 5 years before the trial (Supplementary Table 2). Forty-six participants completed the protocol, and 44 completed all scheduled vaccinations (Fig. 1), for a total of 69 H2HA-Ferritin and 20 H2 DNA administrations. Slower enrollment in the H2-exposed cohort resulted in two enrollments occurring late in the accrual period. To limit the variation in environmental influenza exposure during the influenza season in the participants, the booster vaccination for these two participants (one H2-exposed participant in each prime-boost regimen) was not administered. Both participants remained active in the study for safety and immunogenicity evaluations related to their initial vaccination. One H2-naive participant in the heterologous prime-boost regimen did not receive the second vaccination due to an intercurrent illness but remained active in the study. Three participants moved or were lost to follow-up (one H2-naive participant in the heterologous prime-boost regimen and two H2-naive participants in the homologous prime-boost regimen), and one H2-naive participant in the homologous prime-boost regimen voluntarily withdrew due to time commitment.

### Safety.

**Fig. 2 |:**
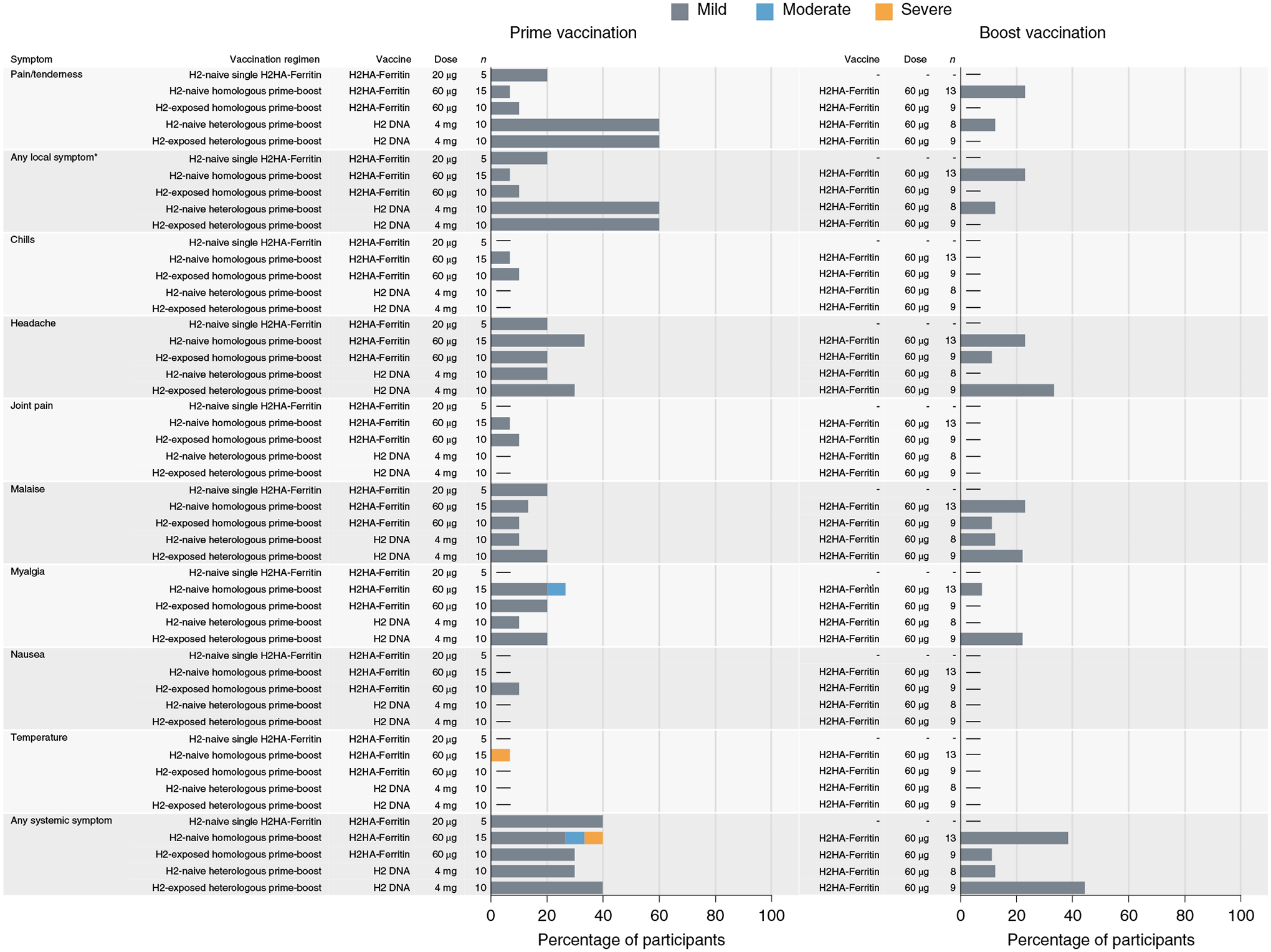
Maximum local and systemic solicited reactogenicity. Percent of participants (*x* axis) who reported a local or systemic symptom (*y* axis) in the 7 d after prime (week 0) or boost (week 16) vaccination. Participants 18–47 years of age (born after 1969) were considered ‘H2-naive’, and those 52–70 years of age (born before 1966) were considered ‘H2-exposed’, based on potential historical exposure to H2N2 influenza. *Local solicited symptoms of swelling or redness were not observed after vaccination.

The primary objective of the clinical trial examined the safety and tolerability of the H2HA-Ferritin and H2 DNA vaccines. Local reactogenicity after H2HA-Ferritin or H2 DNA vaccination was mild, with more reports of mild pain after H2 DNA needle-free administration (*n* = 12, 60%) compared to H2HA-Ferritin administration via needle and syringe (*n* = 7, 10%) (*P* < 0.001) (Fig. 2 and Supplementary Table 3). Most systemic reactogenicity of both vaccines was mild to moderate, with the most common symptom reported being mild headache after both H2HA-Ferritin (*n* = 15, 22%) and H2 DNA (*n* = 5, 25%) administration. One report of a severe fever occurred in an H2-naive participant who received the homologous prime-boost regimen 2 d after the first H2HA-Ferritin vaccination (Supplementary Table 3). This fever coincided with one of the two influenza-like illnesses (ILIs) reported during the trial (Supplementary Table 2). The other ILI occurred in an H2-naive participant in the heterologous prime-boost regimen at 81 d after the H2HA-Ferritin vaccination. Neither ILI was laboratory confirmed as influenza. Six mild to moderate adverse events (AEs) were attributed to vaccination during the trial; all resolved without sequelae (Supplementary Table 4). One serious adverse event (SAE) occurred during the trial but was unrelated to vaccination based on temporality, biological plausibility and alternative causes. On the basis of these results, we determined that these vaccines were safe and well tolerated in this phase 1 clinical trial.

### Response to *H. pylori* ferritin.

**Extended Data Fig. 2 |:**
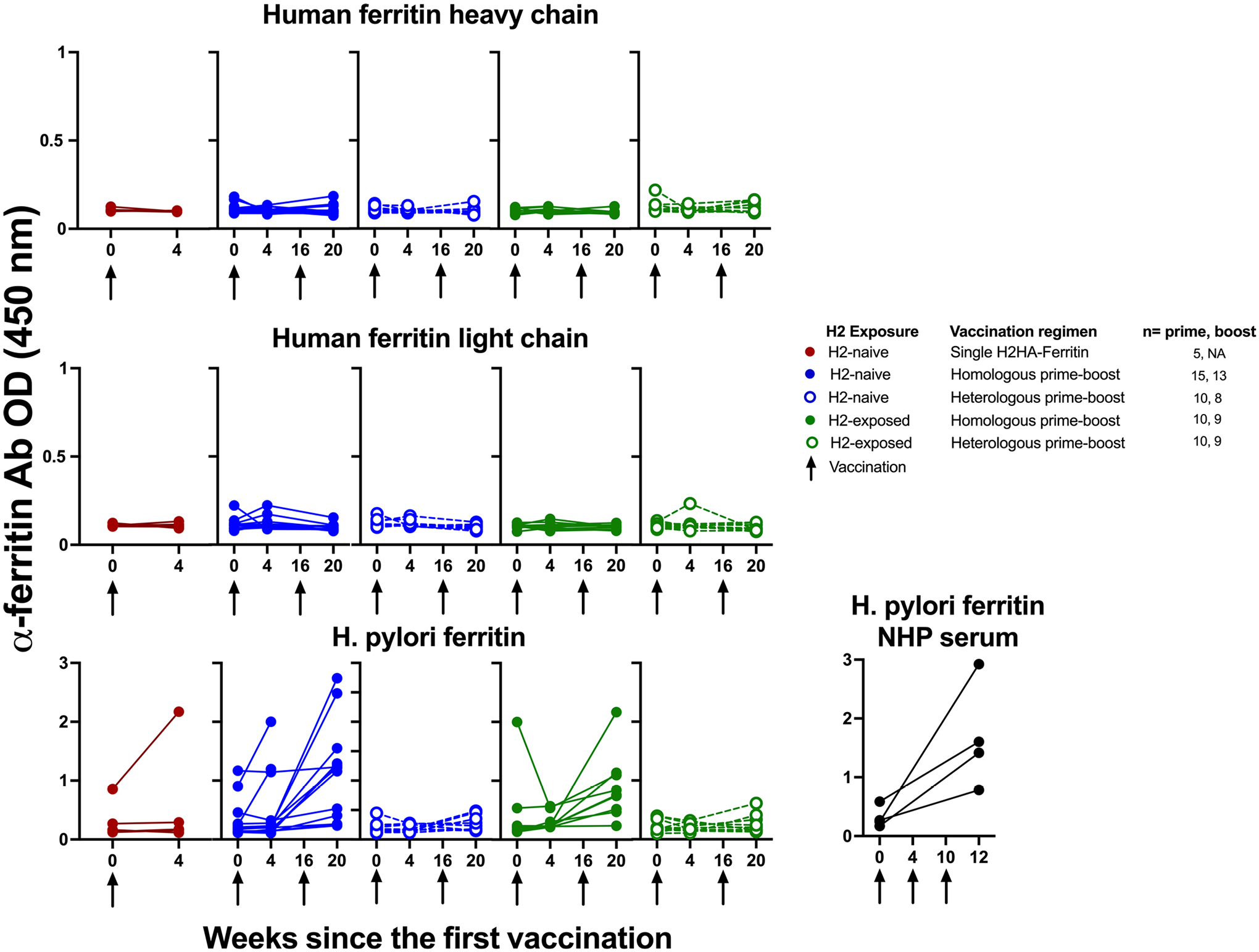
H2HA-Ferritin vaccination did not result in notable antibody development against human ferritin, despite developing a response to H. pylori ferritin. Participant sera were tested by ELISA for antibodies against human ferritin (heavy and light chains) as well as *H. pylori* ferritin at baseline and four weeks after each vaccination. Sera from four NHPs (cynomolgus macaques) vaccinated with an influenza ferritin vaccine at weeks 0, 4, and 10 were included in the *H. pylori* analysis as positive controls. Arrows indicate vaccination time points.

As viral antigen display on *H. pylori*-derived ferritin particles represents a novel vaccine platform, participants were monitored for immunological responses to the ferritin platform throughout the trial. Although *H. pylori* ferritin is highly divergent from mammalian ferritin, we assessed antibody reactivity against both *H. pylori* and human ferritins in an exploratory objective. We detected increases against *H. pylori*-derived ferritin after vaccination in the trial participants, similarly to observations from preclinical models (Extended Data Fig. 2)^11,17^. Notably, no significant increase in antibodies was observed against human ferritin in any participant compared to baseline, indicating that there was no cross-reactive antibody response after one or two doses of H2HA-Ferritin (Extended Data Fig. 2). Additionally, there was no observed effect of H2HA-Ferritin vaccination on monitored hematologic parameters of participants, which included iron levels, hemoglobin levels, hematocrit, white blood cell counts and neutrophil counts.

### Vaccine-induced antibody responses against H2.

**Extended Data Fig. 3 |:**
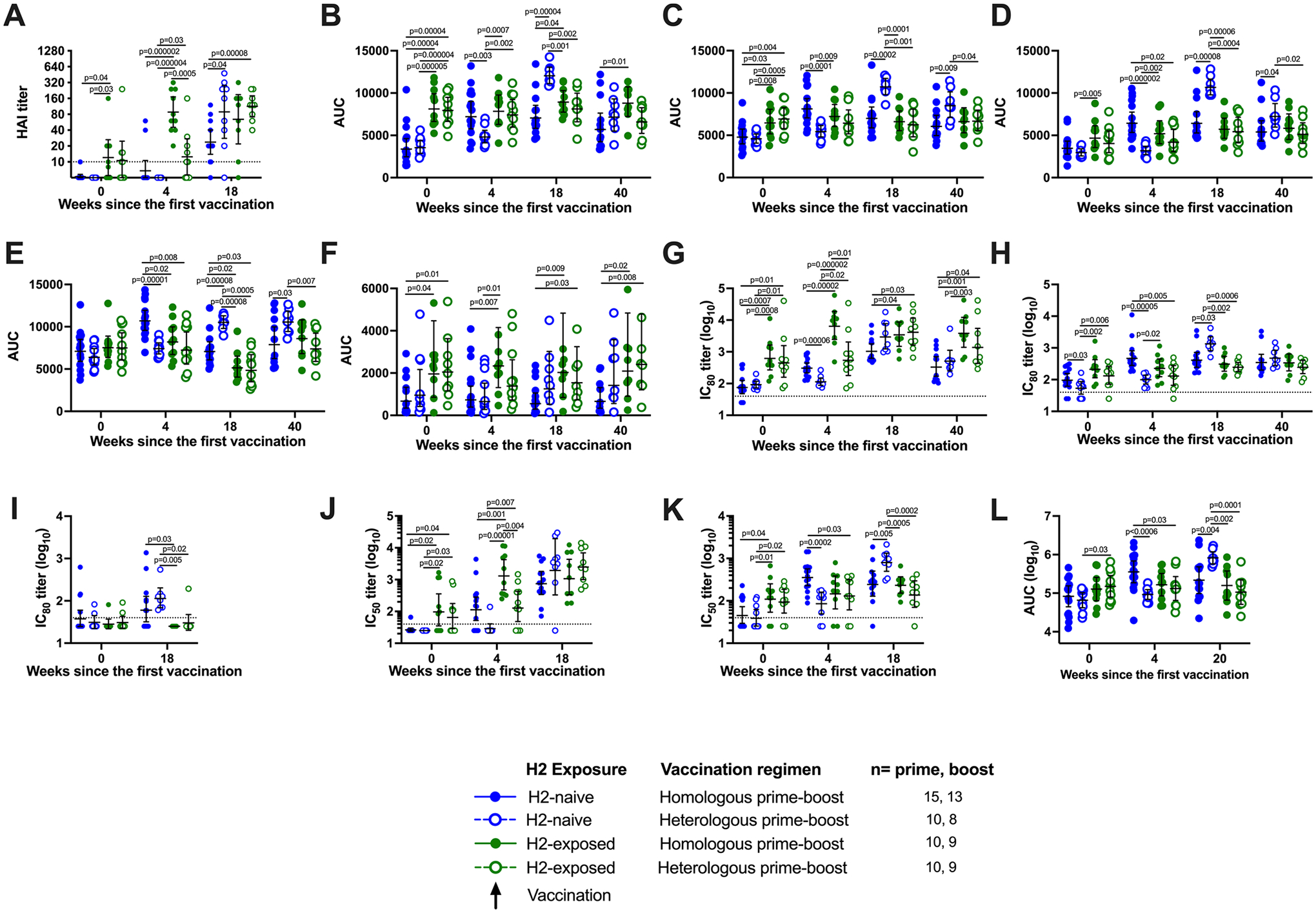
Statistical comparison between groups at key timepoints following H2HA-Ferritin vaccination. Sera from H2-naïve (blue circles) and H2-exposed (green circles) participants who received H2HA-Ferritin in either homologous (closed circles) or heterologous (open circles) prime-boost regimens were compared at select timepoints, including baseline, four weeks after the prime vaccination, two weeks after the boost vaccination (four weeks after the boost for the ADCC assay), and at 40 weeks after the prime vaccination. Results are shown for (**a**) hemagglutination inhibition assays (HAI), (**b**-**f**) HA stem-binding ECLIAs, (**g**-**i**) reporter microneutralization assays, (**j**-**k**) pseudotyped lentivirus neutralization assays, and (**l**) Fc-mediated antibody-dependent cell-mediated cytotoxicity (ADCC) reporter assays. Values displayed represent the group geometric mean titers, with whiskers indicating the 95% confidence intervals. Dotted lines indicate the lower limit of detection. Negative samples were reported and calculated as half the limit of detection. Comparisons between treatment groups were made using two-sided two-sample t-tests, after log_10_ (for the neutralization and ADCC assays) transformations of the raw titers. Number of participant sera analyzed at each time point is summarized in Supplementary Table 7.

**Fig. 3 |:**
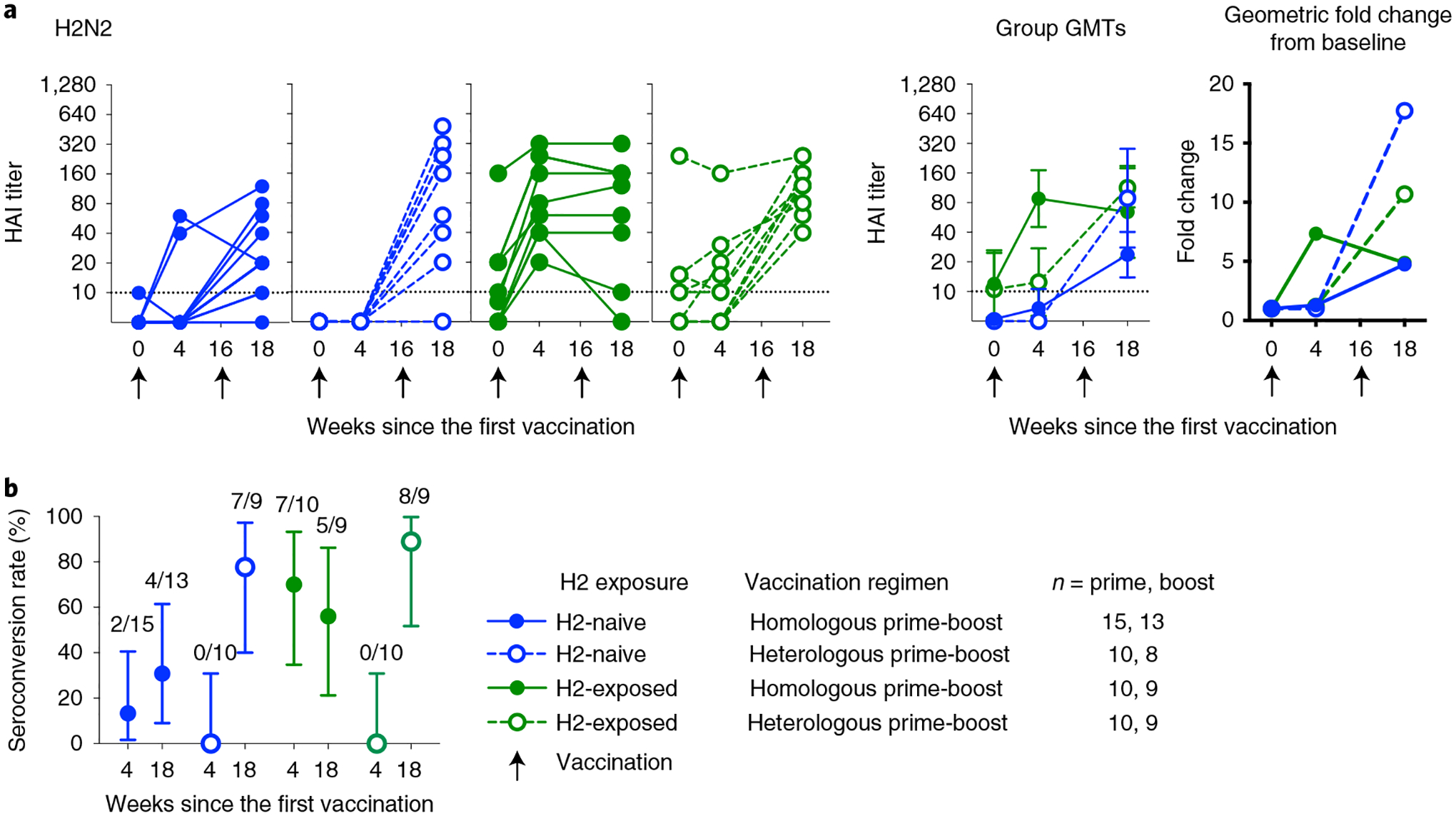
Vaccine-induced binding antibodies are observed by HAI assay after H2HA-Ferritin vaccination. Sera from H2-naive (blue circles) and H2-exposed (green circles) participants who received H2HA-Ferritin in either homologous (closed circles) or heterologous (open circles) prime-boost regimens were analyzed with an HAI assay with H2N2 A/Singapore/1/57. **a**, Individual results, group geometric means and geometric mean fold changes over baseline are shown. **b**, The seroconversion rate for each group is displayed as a percentage, with the number of participants per group seroconverting included above. Whiskers indicate 95% confidence intervals. Dotted lines indicate the lower limit of detection; arrows indicate vaccination time points. Negative samples were reported and calculated as half the limit of detection. The number of participant sera analyzed at each time point is summarized in Supplementary Table 7. GMT, geometric mean titer.

The secondary objective of the clinical trial examined the vaccine-induced antibody response after H2HA-Ferritin administration by hemagglutination inhibition (HAI) assay at baseline, 4 weeks after the prime vaccination and 2 weeks after the boost vaccination. As expected, we did not observe notable levels of baseline antibody responses against H2N2 influenza in H2-naive adults (born after 1969), whereas we detected baseline responses against H2N2 influenza in approximately half of participants representing those who lived through the H2 pandemic (H2-exposed, born before 1966) (Fig. 3a and Extended Data Fig. 3a). These antibodies were observed in the H2-exposed participants after the first dose of H2HA-Ferritin (Fig. 3a). In H2-naive adults, both doses were needed to detect responses by HAI assay in most participants. We observed seroconversion rates between 31% and 89% and at least a four-fold increase in geometric mean HAI assay titers over baseline at 2 weeks after the boost in all vaccination regimens (Fig. 3a,b).

### Vaccine-induced binding antibody responses against HA stem.

**Fig. 4 |:**
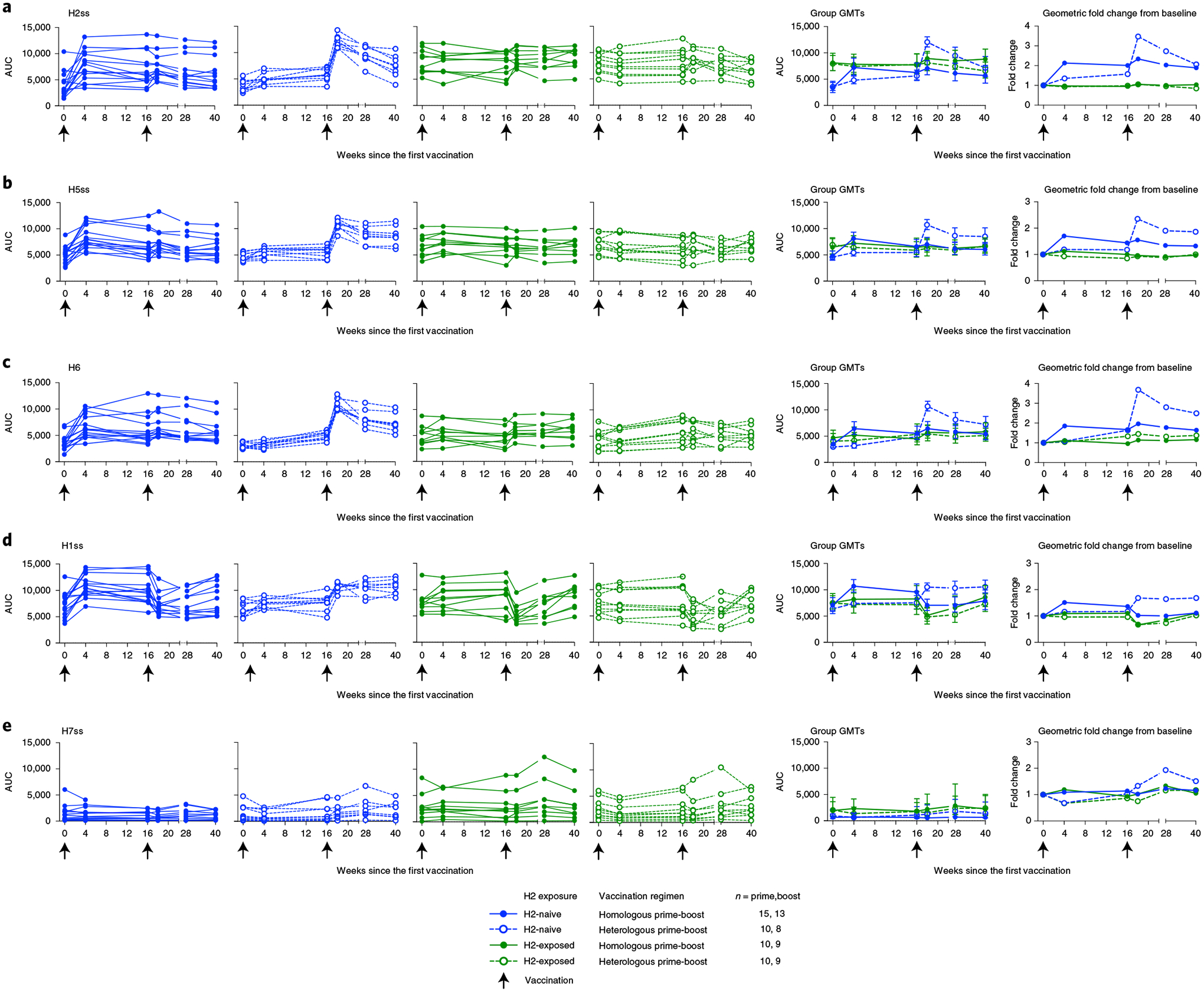
H2HA-Ferritin vaccine platform induces heterosubtypic group 1 HA stem-targeting antibodies in H2-naive adults. Sera from H2-naive (blue circles) and H2-exposed (green circles) participants who received H2HA-Ferritin in either homologous (closed circles) or heterologous (open circles) prime-boost regimens were analyzed for HA stem-binding antibodies against both group 1 and group 2 influenza HA antigens. Individual results, group geometric means and geometric mean fold changes over baseline are shown for group 1 HA stabilized stem (ss) or full-length (FL) antigens, including H2ss (**a**), H5ss (**b**), H6FL (**c**), H1ss (**d**) and the group 2 viruses represented by H7ss (**e**). Whiskers indicate 95% confidence intervals. Dotted lines indicate the lower limit of detection for each assay; arrows indicate vaccination time points. The number of participant sera analyzed at each time point is summarized in Supplementary Table 7. AUC, area under the curve; GMT, geometric mean titer.

To specifically investigate the vaccine-induced antibody response directed against the HA stem, HA stem antibody binding levels were measured by a Meso Scale Discovery electrochemiluminescence immuno assay (ECLIA) as an exploratory objective. We evaluated antibody responses to HA antigens from diverse group 1 influenza subtypes, including H1, H2, H5 and H6, as well as a group 2 subtype represented by H7. The H2 stem antigen was included to assess the response against the homologous virus; the H5 stem antigen was included as the subtype that is a relevant potential avian pandemic threat; the H6 ectodomain antigen was included as a more distantly related subtype within the viruses possessing group 1 HAs; the H1 stem antigen was included for comparison to a seasonal subtype; and the H7 stem antigen was included to look for potential cross-group, stem-specific antibody binding. Because H7 stem-specific responses are poorly elicited by seasonal H3 vaccinations, the background titers to H7 stem are low in the population^18^. In the H2-naive adults, we observed a vaccine-induced increase in binding to the group 1 stem antigens after the first vaccination with H2HA-Ferritin (Fig. 4a–d) that remained for at least 6 months after the boost vaccination (Supplementary Table 5). Across the group 1 subtypes, this geometric fold increase in stem-directed antibodies ranged from 1.5-fold to 2.1-fold after the homologous prime-boost regimen and 1.7-fold to 3.7-fold after the heterologous prime-boost regimen. Similar increases were not observed against the group 2 H7 stem (Fig. 4e). In the H2-exposed adults, no increase in HA-stem binding was observed against any of the proteins evaluated after vaccination, although levels of stem-directed antibodies at baseline trended higher for these participants compared to the H2-naive participants (Fig. 4 and Extended Data Fig. 3b–f).

### Vaccine-induced neutralizing antibody responses against H2.

**Extended Data Fig. 4 |:**
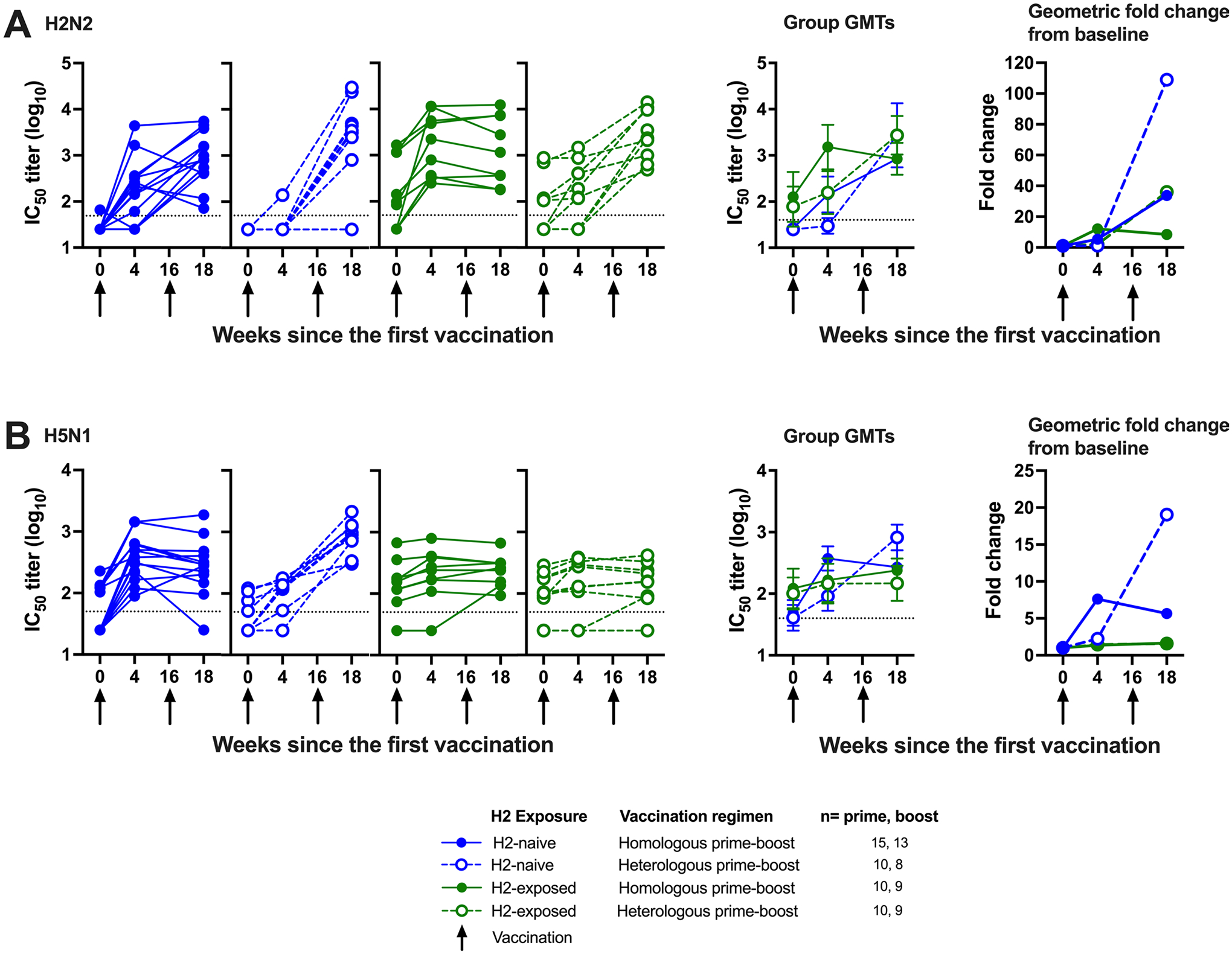
H2HA-Ferritin vaccination induces broadly neutralizing antibodies in H2-naïve adults. Sera from H2-naïve (blue circles) and H2-exposed (green circles) participants who received H2HA-Ferritin in either homologous (closed circles) or heterologous (open circles) prime-boost regimens were analyzed with a pseudotyped lentivirus neutralization assay. Individual results, group geometric means, and geometric mean fold increases over baseline are shown for A) H2N2 A/Singapore/1/57 and B) H5N1 A/Vietnam/1203/04. Whiskers indicate 95% confidence intervals. Dotted lines indicate the lower limit of detection, arrows indicate vaccination timepoints. Negative samples were reported and calculated as half the limit of detection. Number of participant sera analyzed at each time point is summarized in Supplementary Table 7.

**Fig. 5 |:**
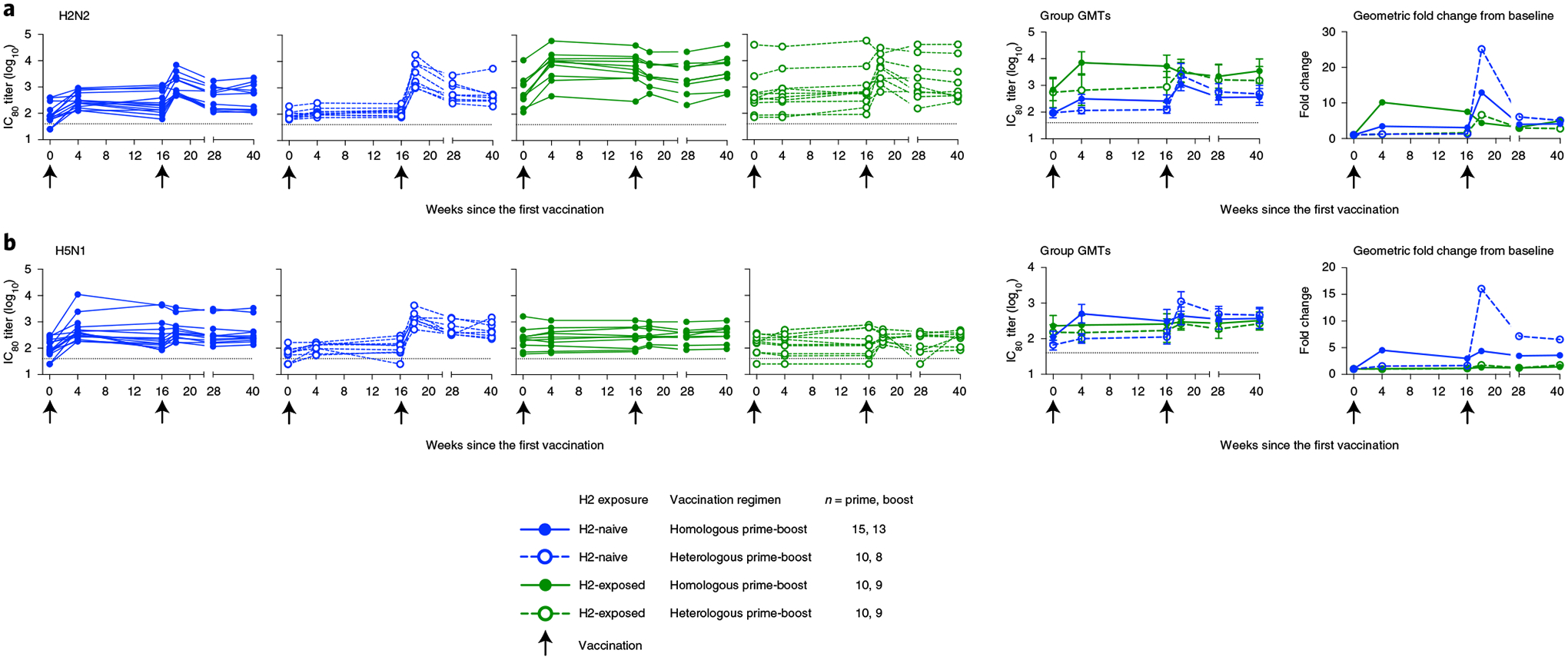
H2HA-Ferritin vaccination induces broadly neutralizing antibodies against group 1 viruses in H2-naive adults. Sera from H2-naive (blue circles) and H2-exposed (green circles) participants who received H2HA-Ferritin in either homologous (closed circles) or heterologous (open circles) prime-boost regimens were analyzed for neutralizing activity (IC_80_) by a reporter-based microneutralization assay. Individual values, group geometric means and geometric mean fold changes over baseline are shown for H2N2 A/Singapore/1/57 (**a**) and H5N1 A/Vietnam/1203/04 (**b**) viruses. Whiskers indicate 95% confidence intervals. Dotted lines indicate the lower limit of detection for each assay; arrows indicate vaccination time points. Negative samples were reported and calculated as half the limit of detection. The number of participant sera analyzed at each time point is summarized in Supplementary Table 7. GMT, geometric mean titer.

We also assessed the neutralizing activity of the vaccine-induced antibodies against the matched H2N2 virus by a reporter microneutralization assay as an exploratory objective. As expected, we observed higher baseline levels of neutralization activity in the H2-exposed adults (Fig. 5 and Extended Data Fig. 3g). We observed an increase in neutralizing activity in both H2-naive and H2-exposed adults after the first vaccination with H2HA-Ferritin (Fig. 5a). In the H2-naive adults who received two doses of H2HA-Ferritin, a further increase was observed after the boost, resulting in a 13-fold increase in geometric mean over baseline. In the H2-naive heterologous prime-boost recipients, a minimal increase in neutralizing activity was observed after the H2 DNA prime; however, a 25-fold increase in geometric mean over baseline was observed after the H2HA-Ferritin boost. For the H2-exposed adults, the homologous and heterologous prime-boost regimens resulted in 4.4-fold and 6.6-fold increases in geometric mean over baseline, respectively. The increase in neutralizing activity remained durable up to the last time point examined at 6 months after the boost vaccination (Supplementary Table 5). These trends were also corroborated with an exploratory pseudotyped lentiviral neutralization assay at key time points (Extended Data Figs. 3j and 4a).

### Effect of participant age on neutralizing antibody response.

**Extended Data Fig. 5 |:**
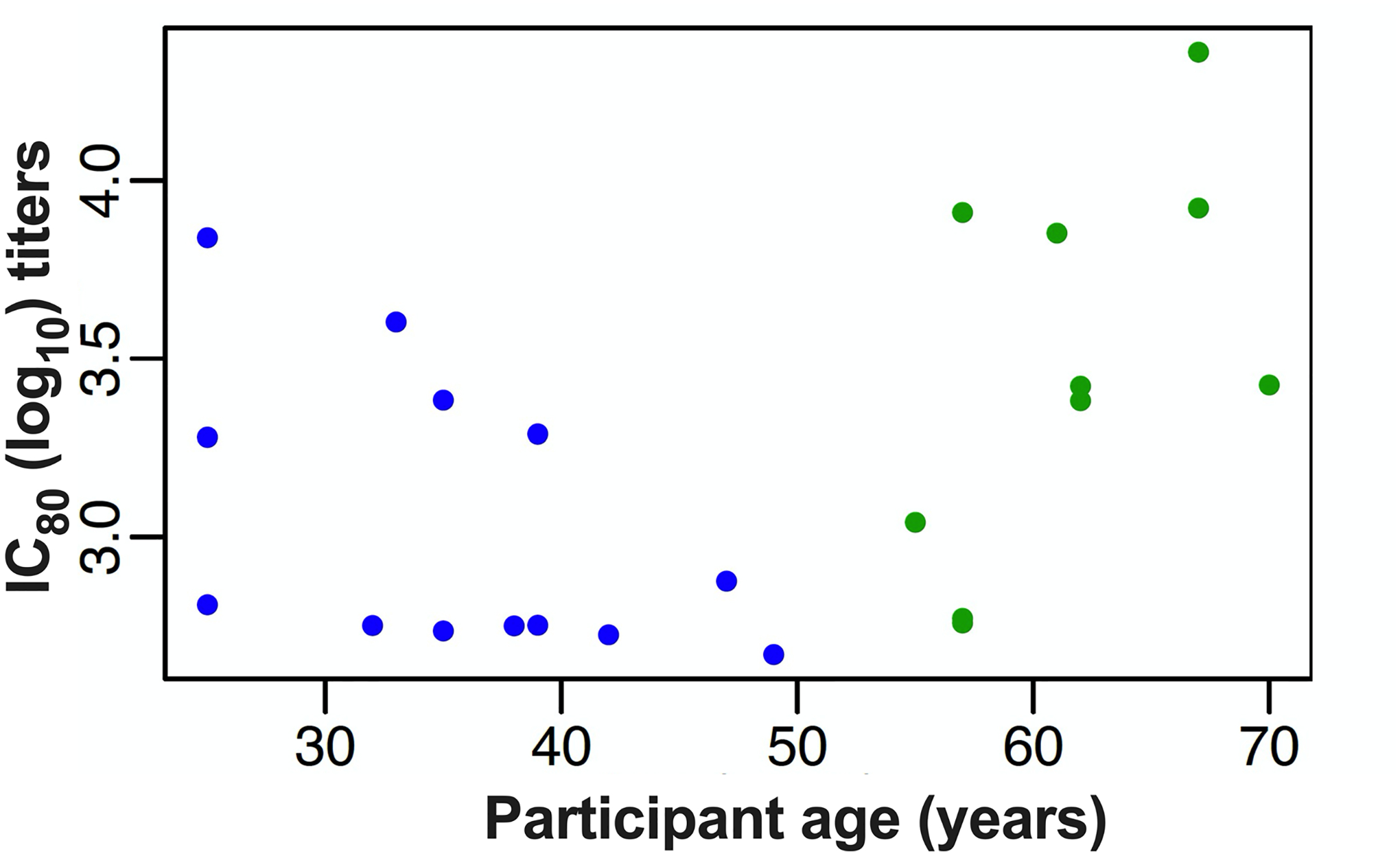
Preexisting and vaccine-induced antibody titers against H2N2 virus compared to age. The neutralizing antibody titers from the reporter microneutralization assay at two weeks after the boost (week 18) for H2-naïve (blue circles, n = 13) and H2-exposed (green circles, n = 9) participants that received two doses of H2HA-Ferritin were compared to the age of the participants at time of enrollment. Two-sided Spearman’s rank correlation coefficient (ρ) and p values for all age groups were ρ=0.39 and p = 0.064. For the H2-naïve adults only, the values were ρ=−0.52 and p = 0.072. For the H2-exposed adults only, the values were ρ=0.6 and p = 0.067.

Because we included such a wide participant age range in this trial and observed a differential vaccine-induced antibody response between the age groups based on H2 exposure status, we sought to rule out the possible effect of immunosenescence in our trial participants. To do this, we conducted a post hoc comparison between the vaccine-induced H2N2 neutralizing antibody titers at 2 weeks after the boost vaccination and the age of participants at the time of enrollment. We observed potential correlations between the neutralizing antibody titers and the age of participants in this small population, with a negative correlation in the H2-naive adults and a positive correlation in the H2-exposed adults (Extended Data Fig. 5). This pattern of increasing neutralizing titers with age in the H2-exposed adults indicates that immunosenescence is not affecting the antibody response in these participants.

### Breadth of vaccine-induced neutralizing antibody responses.

To assess whether the expanded breadth of stem-binding antibody responses after vaccination resulted in increased neutralizing breadth across group 1 viruses in an exploratory objective, we analyzed additional group 1 influenza subtypes with the reporter microneutralization assay, including H5N1 and H6N1. For the H2-naive participants, increases in neutralizing antibodies were observed against H5N1 after the first vaccination with H2HA-Ferritin (Fig. 5b and Extended Data Fig. 3h). After the boost vaccination, 4.4-fold and 16-fold increases in geometric means were observed in the homologous and heterologous prime-boost groups, respectively. The H2-exposed participants did not display an increase in neutralization activity compared to baseline, regardless of prime-boost regimen. These trends were substantiated at 2 weeks after the boost with the more antigenically distant subtype H6N1 (Supplementary Table 6 and Extended Data Fig. 3i) and by an exploratory pseudotyped lentivirus neutralization assay against H5N1 at key time points (Extended Data Figs. 3k and 4b).

**Extended Data Fig. 6 |:**
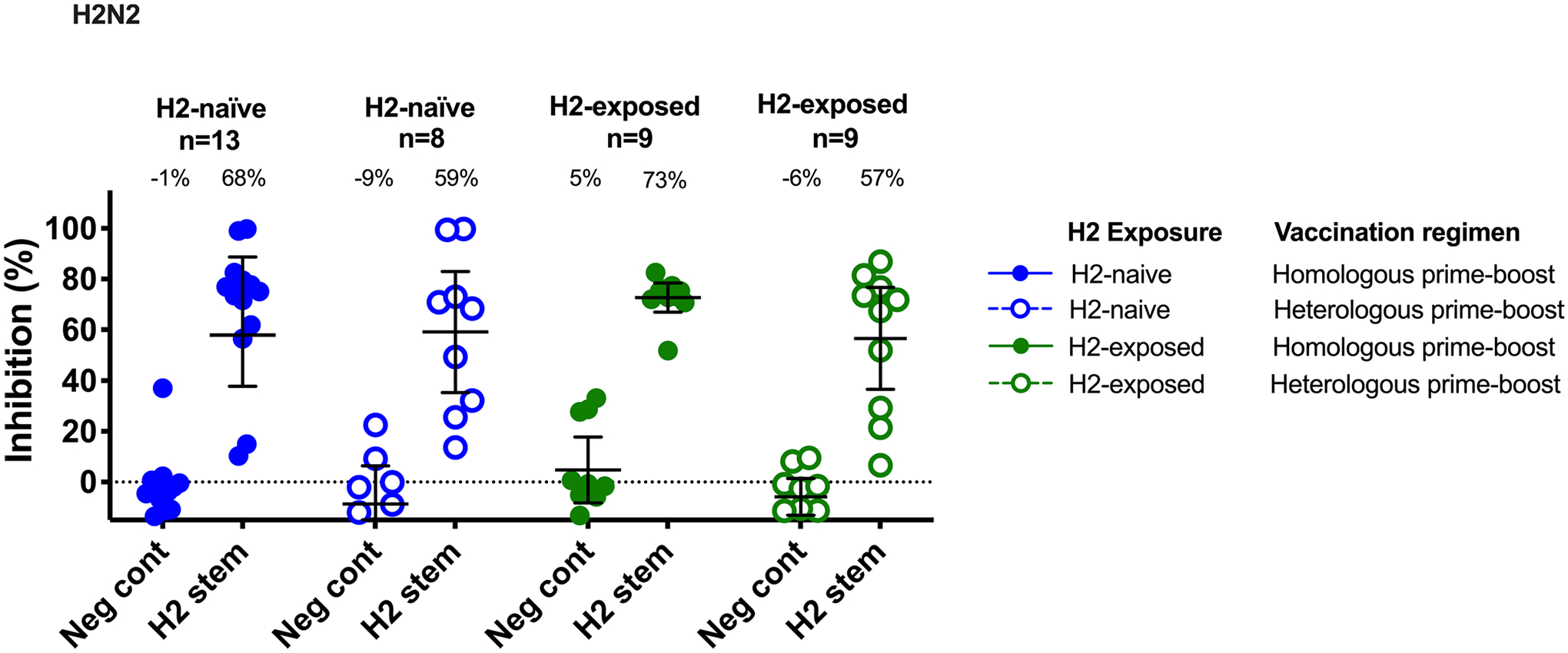
The neutralizing H2HA-Ferritin vaccine-induced antibodies are directed against the HA stem. Sera from H2-naïve (blue circles) and H2-exposed (green circles) participants who received H2HA-Ferritin in either homologous (closed circles) or heterologous (open circles) prime-boost regimens were evaluated by a competition microneutralization assay against homologous H2N2 A/Singapore/1/57 virus at two weeks after the boost vaccination (week 18). Competing antigens include the H2 HA stem and a negative control (DSCav-1). Lines represent group geometric means and whiskers indicate 95% confidence intervals. Dotted lines indicate the lower limit of detection for each assay.

**Fig. 6 |:**
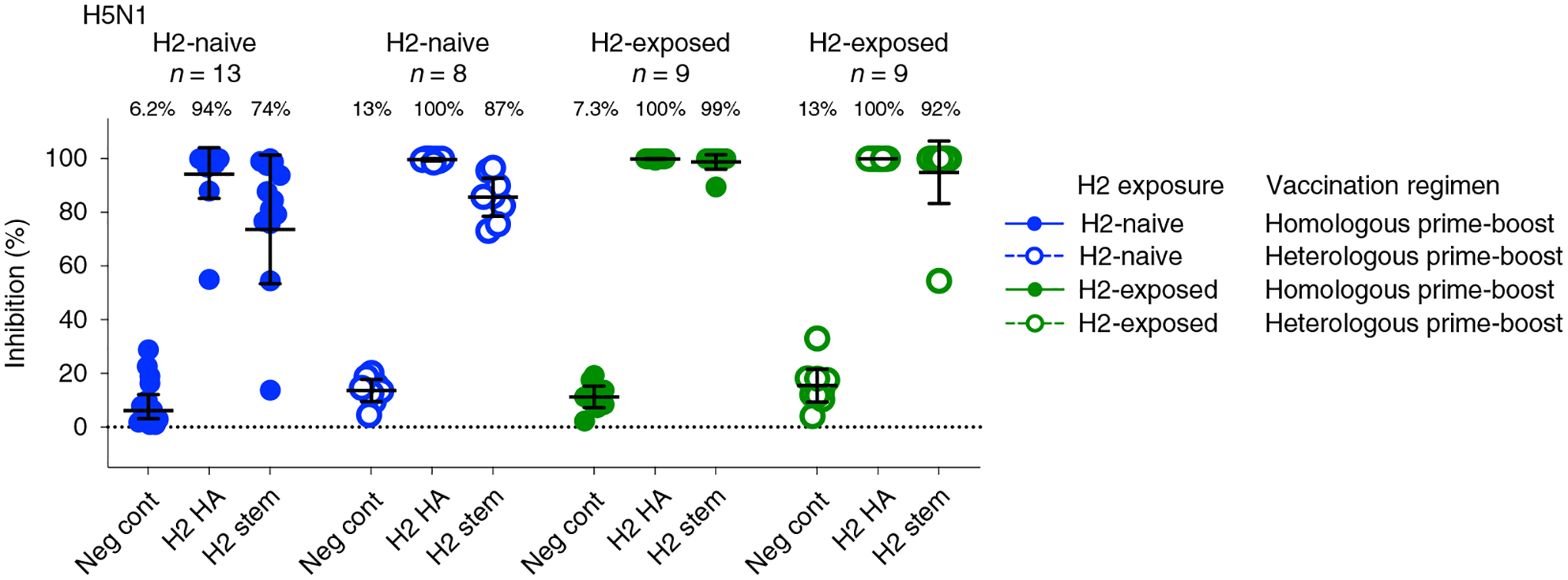
The neutralizing H2HA-Ferritin vaccine-induced antibodies are directed against the HA stem. Sera from H2-naive (blue circles) and H2-exposed (green circles) participants who received H2HA-Ferritin in either homologous (closed circles) or heterologous (open circles) prime-boost regimens were evaluated by a competition microneutralization assay against heterologous H5N1 A/Vietnam/1203/04 virus at 2 weeks after the boost vaccination (week 18). Competing antigens include the full-length H2 HA, H2 HA stem and a negative control (DSCav-1). Lines represent group geometric means, and whiskers indicate 95% confidence intervals. Dotted lines indicate the lower limit of detection for the assay.

To confirm that the broad neutralizing activity observed against the heterosubtypic viruses was targeting the HA stem specifically, we performed a neutralization assay in the presence of competitor antigens on sera at 2 weeks after the boost as an exploratory objective (Fig. 6). Competing antigens, including a full-length H2 HA and an H2 stem antigen, were added to participant sera before incubation with the H5N1 virus. If the antibodies present in the serum bind to the competing antigen, the neutralization of the H5N1 virus will be inhibited, allowing us to define the specificity of the antibody response. As expected, the full-length H2 HA antigen resulted in almost complete inhibition of neutralizing activity against the H5N1 virus in all groups, whereas a negative control antigen (DSCav-1) inhibited only between 6% and 13% of the neutralization activity against H5N1 (Fig. 6). These trends were confirmed through inhibition of the neutralization activity against an H2N2 virus (Extended Data Fig. 6). The H2 stem antigen was able to inhibit most neutralizing activity against both the H5N1 and H2N2 viruses, indicating that the HA stem is a major target of H2HA-Ferritin vaccine-induced broadly neutralizing antibodies.

### HA stem-specific Fc-mediated antibody activity.

**Extended Data Fig. 7 |:**
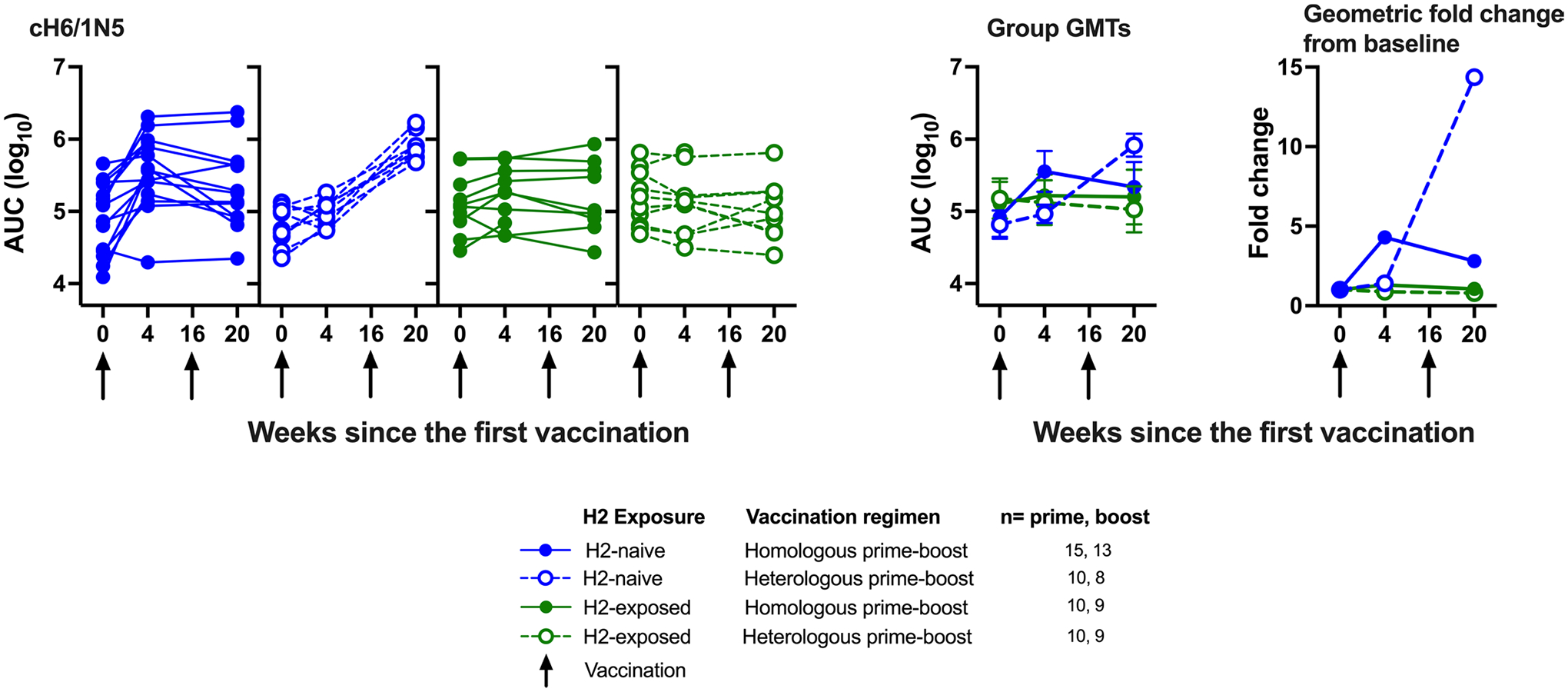
The vaccine-induced antibodies display Fc-mediated antibody-dependent cell-mediated cytotoxicity (ADCC) activity. Sera from H2-naïve (blue circles) and H2-exposed (green circles) participants who received H2HA-Ferritin in either homologous (closed circles) or heterologous (open circles) prime-boost regimens were analyzed for Fc-mediated antibody-dependent cell-mediated cytotoxicity (ADCC) activity. Group geometric means, geometric mean fold increases over baseline, and individual values are shown for against the recombinant H6/1N5 virus. Whiskers indicate 95% confidence intervals. Dotted lines indicate the lower limit of detection for each assay, arrows indicate vaccination time points. Number of participant sera analyzed at each time point is summarized in Supplementary Table 7.

In addition to neutralizing activity, stem-directed antibodies have been reported to exhibit Fc-mediated antibody-dependent cellular cytotoxicity (ADCC) activity^19^. To assess HA stem-specific Fc-mediated activity as an exploratory objective, we used an ADCC reporter assay with a chimeric HA containing a head region from an antigenically distant group 1 subtype (H6) and a stem region from a seasonal group 1 subtype (H1)^20^. Corresponding with our HA stem-directed neutralizing antibody results, we detected an increase in Fc-mediated ADCC activity in H2-naive adults after vaccination with H2HA-Ferritin (Extended Data Figs. 3l and 7). At 4 weeks after the boost vaccination, the H2-naive adults who received homologous and heterologous prime-boost regimens displayed 2.8-fold and 14.4-fold increases in geometric means over baseline, respectively (Extended Data Fig. 7). The baseline Fc-mediated ADCC activity in H2-exposed adults was unaffected by vaccination. Overall, these trial results indicate that the H2HA-Ferritin vaccine platform elicits broadly neutralizing responses in H2-naive adults through the production of antibodies against the conserved HA stem and that these antibodies have both neutralizing and Fc-mediated activity.

## Discussion

This phase 1 clinical trial of a novel ferritin nanoparticle-based influenza vaccine shows that H2HA-Ferritin is safe, well tolerated and immunogenic in healthy adults. This represents the first-in-human evaluation of this ferritin nanoparticle-based vaccine platform and is a proof of concept for a new generation of vaccines that display orderly arrays of antigens on self-assembling nanoparticles. We found the H2HA-Ferritin vaccine to be immunogenic, resulting in increased neutralizing activity in all participants. Notably, H2HA-Ferritin also induced broadly neutralizing antibodies directed against the conserved HA stem in H2-naive adults. Owing to the breadth of response induced, these results indicate a potential use for this ferritin nanoparticle-based antigen display platform in pandemic vaccine preparedness and for universal influenza vaccine development^10,21^.

Vaccination with H2HA-Ferritin led to increases in HAI assay activity in all participants in this trial. The HAI reciprocal titers observed in this trial were equivalent to or higher than previous clinical trials investigating H2-inactivated or live-attenuated vaccines in H2-naive adults^22–24^. Interestingly, we did not observe an increase in reciprocal HAI assay titers after the second dose of H2HA-Ferritin in the H2-exposed adults. Previous studies indicated that repeated vaccinations against the same influenza antigen can result in smaller increases in HAI assay titers or even decreased HAI titers^25^. Post-vaccination responses in individuals who receive multiple vaccinations can display smaller fold rises and similar final HAI assay titers compared to individuals who received a single vaccination^25^. These phenomena might explain the trends in HAI antibody levels that we observed in the H2-naive and H2-exposed adults in this trial. Thus, the prime-boost interval used, based on previous trials involving DNA primes^26^, might not be optimal for the ferritin nanoparticle platform in immunologically primed individuals. Furthermore, we cannot rule out the possibility that previously H2-exposed individuals with baseline responses are immunologically clearing vaccine particles before they can elicit an increase in HAI assay antibody levels.^27^

In comparing the two vaccination regimens evaluated in this trial, the heterologous prime-boost vaccine regimen consistently elicited improved heterologous responses compared to the homologous prime-boost regimen, consistent with previous DNA priming trials^16,28^. At 2 weeks after the boost, the H2-naive participants primed with DNA showed the highest HA stem-specific titers as well as the greatest geometric mean fold increases of neutralizing activity and ADCC activity. However, this improvement appeared temporary, as the group geometric mean neutralizing activity did not differ between regimens by 6 months after the boost. Together, these results show that the H2HA-Ferritin vaccine can be used in both heterologous and homologous prime-boost regimens, with a transient modest improvement in antibody responses with the addition of a DNA prime.

Notably, we observed the induction of neutralizing antibodies in all trial participants. Remarkably, this response was observed after the initial H2HA-Ferritin vaccination in both H2-naive and H2-exposed adults. Unadjuvanted protein-based influenza vaccines typically require multiple injections to result in a consistent response in seronegative individuals^29^. It is possible that the addition of an adjuvant could further improve the H2HA-Ferritin response and could be included in future evaluations of this novel platform^30^. The observed neutralizing antibody response in the trial participants was durable, and remained for at least 6 months after the boost vaccination (Supplementary Table 5).

A key aim for improved influenza vaccine development is to generate broadly neutralizing antibody responses against conserved viral epitopes^10,21,31^. Our unique trial design involving the age stratification of participants and the use of H2N2 influenza allowed us to assess a novel candidate vaccine using an influenza subtype with pandemic potential in two distinct immunological populations: those alive while H2 influenza circulated in humans (H2-exposed, born before 1966) versus those born after H2 influenza stopped circulating (H2-naive, born after 1969). Although vaccination with H2HA-Ferritin did induce neutralizing antibodies against the H2 subtype in all participants, we observed key differences in the antibody response between the H2-naive and H2-exposed participants. Notably, we elicited responses to conserved epitopes in the H2-naive participants in this trial and identified responses directed against the conserved HA stem. Such HA stem-directed antibodies have been shown to neutralize a wide range of influenza viruses in vitro and have been proven to provide protection in both animal model viral challenges and human infections^17,32,33^. The HA stem-directed antibodies elicited in this trial also exhibited Fc-mediated ADCC activity, suggesting that these antibodies could facilitate both neutralization and cell-mediated protection against a range of group 1 influenza viruses^10,31^. Although HA stem-directed antibodies were observed in the H2-exposed adults, the vaccine-induced increase in these participants was limited. These results are consistent with previous vaccine studies showing immunodominance of the head domain in recall responses, contrasting with stem-directed responses in individuals where an HA head-dominated antibody response has not been established^34–39^. Although the head-dominated responses were a limitation of the full-length HA antigen used in this trial, we have also designed stabilized stem formats of this ferritin-based nanoparticle vaccine platform^17^ that lack the immunodominant head domain of the HA protein. These stem-only antigens are currently being evaluated in phase 1 trials (ClinicalTrials.gov, NCT03814720 and NCT04579250) and might overcome the HA head immunodominance to provide additional opportunities for vaccine development.

Beyond annual epidemics with seasonal influenza strains, influenza pandemics continue to emerge in the human population at unpredictable intervals^3^. The H2N2 subtype evaluated with this vaccine circulated in humans between 1957 and 1968 and retains strong pandemic potential^14^. The pandemic potential of H2N2 influenza is likely higher than other non-endemic subtypes, because H2N2 viruses have previously transmitted efficiently among humans and still circulate in avian reservoirs, and a large segment of the population is currently immunologically H2-naive. As such, there remains an unmet public health need for vaccine development for this subtype^14^. Current research indicates that the H2 HA stem is more amenable to boosting cross-reactive HA stem-directed antibody responses compared to other group 1 subtypes, such as H5 (Andrews et al., accepted at *Nature Medicine*). The production of neutralizing antibodies induced by H2HA-Ferritin vaccination in the trial participants irrespective of their previous H2 exposure highlights the utility in the broad population of this platform for pandemic preparedness and response.

This phase 1 trial has limitations, and these aspects deserve greater consideration in future evaluations of this vaccine platform. In this trial, the immunological analysis performed was mainly descriptive. Future analyses of this platform should evaluate the protective capacity of the vaccine-induced antibodies. We observed a limited effect of the boost on HAI assay titers, and future trials should include additional prime-boost intervals with the goal of improving these responses. Ongoing trials evaluating nanoparticle vaccines expressing seasonal influenza HA subtypes will also help determine if these response patterns are specific to H2 influenza. We detected a strong response against the *H. pylori* ferritin backbone after vaccination in this trial. In preclinical models, a pre-existing response to *H. pylori* ferritin was not found to impede responses to boost vaccinations^11^. As with many phase 1 trials, the application of these results is limited by the small number of participants, but the results show that this novel vaccine platform warrants further clinical evaluation.

Remarkably, the H2HA-Ferritin vaccine evaluated in this trial elicited broad heterosubtypic neutralizing HA stem-directed antibodies in all H2-naive participants after a single dose. In addition to this platform’s favorable immunogenic properties, its design and construction can be rapidly initiated with HA sequence alone, does not require live virus or eggs in the manufacturing process and is compatible with most genetic delivery platforms, including mRNA^40^. The ferritin nanoparticle platform has been licensed non-exclusively for future clinical development. These trial results underscore the opportunity for this platform to improve seasonal influenza virus vaccines, to prepare and respond to pandemics and to advance the field toward a more universal influenza vaccine approach.

## Methods

### Study design.

VRC 316 was a phase 1, open-label, dose-escalation, randomized trial (ClinicalTrials.gov, NCT03186781) to examine the H2HA-Ferritin vaccine in both homologous and heterologous prime-boost regimens in healthy adults aged 18–70 years, excluding those born between 1966 and 1969 in an attempt to enroll two distinct cohorts based on potential historical H2N2 exposure. Inclusion criteria required general good health determined by laboratory tests, medical history and physical examination, with receipt of at least one licensed influenza vaccine since 2014. Exclusion criteria included previous receipt of a licensed influenza vaccine within 6 weeks, or H2N2 influenza vaccine at any time, before trial enrollment. A complete list of inclusion and exclusion criteria can be found in the trial protocol (https://clinicaltrials.gov/ProvidedDocs/81/NCT03186781/Prot_SAP_ICF_000.pdf). Volunteers were recruited from the greater Washington, DC, area by institutional review board (IRB)-approved written and electronic media. The study was conducted at the National Institutes of Health (NIH) Clinical Center by the Vaccine Research Center (VRC) Clinical Trials Program, National Institute of Allergy and Infectious Diseases (NIAID), NIH, in Bethesda, Maryland. The trial protocol was reviewed and approved by the NIAID IRB. Written informed consent was obtained from all participants before enrollment. The trial provided compensation for participant time and travel.

The H2HA-Ferritin (VRC-FLUNPF081–00-VP) and H2 DNA (VRC-FLUDNA082–00-VP) vaccines were manufactured under Good Manufacturing Practice at the VRC Pilot Plant operated under contract by Vaccine Clinical Materials Program, Leidos Biomedical Research. H2HA-Ferritin is composed of the A/Singapore/1/1957 HA genetically fused to the *H. pylori*-derived ferritin protein. The H2 HA construct in H2HA-Ferritin contains amino acids 1–514, with a Y98F mutation to abolish non-specific binding to sialic acid (based on H3 numbering). The H2HA-Ferritin vaccine displays eight HA trimers on the surface of each self-assembled ferritin particle. The H2 DNA vaccine is a single closed circular plasmid that encodes the matched full-length transmembrane-anchored A/Singapore/1/1957 HA. All study injections were administered intramuscularly in the deltoid muscle. The H2HA-Ferritin vaccine was administered via needle and syringe, whereas the H2 DNA vaccine was administered via the needle-free Stratis device (PharmaJet).

### Procedures.

This trial evaluated a dose escalation of H2HA-Ferritin for safety and evaluated H2HA-Ferritin in both homologous and heterologous prime-boost regimens (Fig. 1). Participants in the single-dose group received a 20-μg dose of H2HA-Ferritin at week 0. Participants in the homologous prime-boost regimen groups received a 60-μg dose of H2HA-Ferritin at weeks 0 and 16. Participants in the heterologous prime-boost regimen groups received a 4-mg dose of H2 DNA at week 0 and a 60-μg dose of H2HA-Ferritin at week 16. Enrollment opened for the dose-escalation and prime-boost regimens after protocol-specified interim safety reviews by the Protocol Safety Review Team. Participants were evaluated for 40 weeks after the first vaccination.

Laboratory tests were obtained before vaccine administration and throughout the study to assess safety. Hematological monitoring of participant sera included iron levels, hemoglobin levels, hematocrit, white blood cell counts and neutrophil counts. Participants also recorded solicited symptoms for 7 d after each vaccination, and a clinician assessed the site of vaccination on the day of administration, the next day and 1 week later. All unsolicited AEs were recorded for 28 d after each administration, whereas ILIs, SAEs and new chronic medical conditions were recorded throughout the duration of the study. Participant safety data were reviewed weekly by the Protocol Safety Review Team up to 4 weeks after the final vaccine administration and then monthly through the completion of the final study visit.

Serum samples were collected throughout the trial to evaluate antibody responses at baseline and after each vaccination. Serum samples were evaluated repeatedly and in duplicate or more for the HAI assays, HA stem-binding ECLIAs, reporter-based microneutralization assays, pseudotyped lentiviral neutralization assays and ADCC reporter assays and for antibodies against ferritin proteins as described in the supplementary appendix. Investigators were blinded to group allocation until sample testing was complete. All unique materials used in this study are available from the authors upon reasonable request.

### Randomization.

Participants were age-stratified and randomized 1:1 in the prime-boost regimens during the vaccine regimen evaluation portion of the trial. The randomization sequence was prepared in advance by the study statistician using block sizes of six or eight and was provided to the study site pharmacy and data management center. Study clinicians enrolled participants, and vaccinations were administered open-label.

### Outcomes and statistical analyses.

The primary outcome of this phase 1 trial examined the safety and tolerability of H2HA-Ferritin and H2 DNA in healthy adults. The secondary outcome evaluated the vaccine-induced antibody response of each vaccination regimen at baseline, 4 weeks after the prime vaccination and 2 weeks after the boost vaccination.

All participants who received at least one vaccination were analyzed for safety and reactogenicity, and all participants were included in the immunogenicity analysis until their vaccination schedule was discontinued or changed. Sample size for this trial was based on the primary endpoint of safety, and the calculations are detailed in the trial protocol. These calculations for safety were predetermined and expressed in terms of the ability to detect SAEs. Sample sizes were chosen so that, in the dose-escalation portion of the trial, there was a 90% chance to observe at least one SAE if the true rate was at least 0.369 and over a 90% chance to observe no SAE if the true rate was less than 0.021. In the vaccine regimen evaluation portion of the trial, there was over a 90% chance to observe at least one SAE if the true rate was at least 0.206 and over a 90% chance to observe no SAE if the true rate was no more than 0.010. The study was not designed to detect small immunogenic differences between groups but was designed to detect large immunogenic differences between groups. Adjustments for multiple comparisons were not performed. Groups 2 and 4A were combined for the immunological analysis because they received the same vaccination regimen with identical age ranges. Seroconversion rates for the HAI assay were determined per the US Food and Drug Administration definition of either a baseline HAI assay titer of <1:10 and a post-boost HAI assay titer of ≥1:40 or a baseline HAI assay titer of ≥1:10 and a minimum four-fold rise from baseline^41^. Comparisons between treatment groups were made using two-sample *t*-tests after log_10_ or log_2_ transformations of the raw titers (when applicable). Correlations were quantified using Spearman’s ρ. Fold change summaries were reported using raw titers. All statistical tests were two sided. For all assays, negative samples were reported, and geometric mean titers were calculated using half the limit of detection.

### Reporting Summary.

Further information on research design is available in the Nature Research Reporting Summary linked to this article.

## Extended Data

## Supplementary Material

Supplementary Materials

## Data Availability

Data generated in this study are available as de-identified data on ClinicalTrials.gov (NCT03186781). The study protocol, statistical analysis plan and informed consent form are available on ClinicalTrials.gov (https://clinicaltrials.gov/ProvidedDocs/81/NCT03186781/Prot_SAP_ICF_000.pdf). Individual participant data that underlie the results reported in this article are available, after de-identification, in the Supplementary Information immediately after publication with no end date. Additional data may be made available upon reasonable request to the corresponding author for investigators whose proposed use of the data has been approved by the National Institute of Allergy and Infectious Diseases institutional review board.

## References

[1] World Health Organization. Seasonal influenza. http://www.euro.who.int/en/health-topics/communicable-diseases/influenza/seasonal-influenza (2021).

[2] Iuliano AD Estimates of global seasonal influenza-associated respiratory mortality: a modelling study. Lancet 391, 1285–1300 (2018). 29248255 10.1016/S0140-6736(17)33293-2 PMC5935243

[3] Taubenberger JK, Kash JC & Morens DM The 1918 influenza pandemic: 100 years of questions answered and unanswered. Sci. Transl. Med 11, eaau5485 (2019). 10.1126/scitranslmed.aau5485 PMC11000447 31341062

[4] Wei CJ Next-generation influenza vaccines: opportunities and challenges. Nat. Rev. Drug Discov 19, 239–252 (2020). 32060419 10.1038/s41573-019-0056-x PMC7223957

[5] Joyce MG Vaccine-induced antibodies that neutralize group 1 and group 2 influenza A viruses. Cell 166, 609–623 (2016). 27453470 10.1016/j.cell.2016.06.043 PMC4978566

[6] Grohskopf LA Prevention and control of seasonal influenza with vaccines: recommendations of the advisory committee on immunization practices—United States, 2020–21 influenza season. MMWR Recomm. Rep 69, 1–24 (2020). 10.15585/mmwr.rr6908a1 PMC7439976 32820746

[7] Sui J Structural and functional bases for broad-spectrum neutralization of avian and human influenza A viruses. Nat. Struct. Mol. Biol 16, 265–273 (2009). 19234466 10.1038/nsmb.1566 PMC2692245

[8] Ekiert DC A highly conserved neutralizing epitope on group 2 influenza A viruses. Science 333, 843–850 (2011). 21737702 10.1126/science.1204839 PMC3210727

[9] Corti D A neutralizing antibody selected from plasma cells that binds to group 1 and group 2 influenza A hemagglutinins. Science 333, 850–856 (2011). 21798894 10.1126/science.1205669

[10] Nabel GJ & Fauci AS Induction of unnatural immunity: prospects for a broadly protective universal influenza vaccine. Nat. Med 16, 1389–1391 (2010). 21135852 10.1038/nm1210-1389

[11] Kanekiyo M Self-assembling influenza nanoparticle vaccines elicit broadly neutralizing H1N1 antibodies. Nature 499, 102–106 (2013). 23698367 10.1038/nature12202 PMC8312026

[12] Nachbagauer R A chimeric haemagglutinin-based influenza split virion vaccine adjuvanted with AS03 induces protective stalk-reactive antibodies in mice. NPJ Vaccines 1, 16015 (2016). 29250436 10.1038/npjvaccines.2016.15 PMC5707880

[13] Impagliazzo A A stable trimeric influenza hemagglutinin stem as a broadly protective immunogen. Science 349, 1301–1306 (2015). 26303961 10.1126/science.aac7263

[14] Nabel GJ, Wei CJ & Ledgerwood JE Vaccinate for the next H2N2 pandemic now. Nature 471, 157–158 (2011). 21390107 10.1038/471157a

[15] Jones JC Risk assessment of H2N2 influenza viruses from the avian reservoir. J. Virol 88, 1175–1188 (2014). 24227848 10.1128/JVI.02526-13 PMC3911670

[16] Ledgerwood JE DNA priming and influenza vaccine immunogenicity: two phase 1 open label randomised clinical trials. Lancet Infect. Dis 11, 916–924 (2011). 21975270 10.1016/S1473-3099(11)70240-7 PMC7185472

[17] Yassine HM Hemagglutinin-stem nanoparticles generate heterosubtypic influenza protection. Nat. Med 21, 1065–1070 (2015). 26301691 10.1038/nm.3927

[18] Krammer F H3 stalk-based chimeric hemagglutinin influenza virus constructs protect mice from H7N9 challenge. J. Virol 88, 2340–2343 (2014). 24307585 10.1128/JVI.03183-13 PMC3911549

[19] Vanderven HA, Jegaskanda S, Wheatley AK & Kent SJ Antibody-dependent cellular cytotoxicity and influenza virus. Curr. Opin. Virol 22, 89–96 (2017). 28088123 10.1016/j.coviro.2016.12.002

[20] DiLillo DJ, Tan GS, Palese P & Ravetch JV Broadly neutralizing hemagglutinin stalk-specific antibodies require FcγR interactions for protection against influenza virus in vivo. Nat. Med 20, 143–151 (2014). 24412922 10.1038/nm.3443 PMC3966466

[21] Sangesland M & Lingwood D Antibody focusing to conserved sites of vulnerability: the immunological pathways for ‘universal’ influenza vaccines. Vaccines (Basel) 9, 125 (2021). 33562627 10.3390/vaccines9020125 PMC7914524

[22] Hehme N, Engelmann H, Kunzel W, Neumeier E & Sanger R Pandemic preparedness: lessons learnt from H2N2 and H9N2 candidate vaccines. Med. Microbiol. Immunol 191, 203–208 (2002). 12458361 10.1007/s00430-002-0147-9

[23] Talaat KR An open-label phase I trial of a live attenuated H2N2 influenza virus vaccine in healthy adults. Influenza Other Respir. Viruses 7, 66–73 (2013). 22417012 10.1111/j.1750-2659.2012.00350.x PMC3527634

[24] Isakova-Sivak I H2N2 live attenuated influenza vaccine is safe and immunogenic for healthy adult volunteers. Hum. Vaccin. Immunother 11, 970–982 (2015). 25831405 10.1080/21645515.2015.1010859 PMC4514355

[25] Belongia EA Repeated annual influenza vaccination and vaccine effectiveness: review of evidence. Expert Rev. Vaccines 16, 1–14 (2017). 10.1080/14760584.2017.1334554 28562111

[26] Ledgerwood JE Prime-boost interval matters: a randomized phase 1 study to identify the minimum interval necessary to observe the H5 DNA influenza vaccine priming effect. J. Infect. Dis 208, 418–422 (2013). 23633407 10.1093/infdis/jit180 PMC3699006

[27] Zarnitsyna VI, Lavine J, Ellebedy A, Ahmed R & Antia R Multi-epitope models explain how pre-existing antibodies affect the generation of broadly protective responses to Influenza. PLoS Pathog 12, e1005692 (2016). 27336297 10.1371/journal.ppat.1005692 PMC4918916

[28] DeZure AD An avian influenza H7 DNA priming vaccine is safe and immunogenic in a randomized phase I clinical trial. NPJ Vaccines 2, 15 (2017). 29263871 10.1038/s41541-017-0016-6 PMC5627236

[29] Wong SS & Webby RJ Traditional and new influenza vaccines. Clin. Microbiol. Rev 26, 476–492 (2013). 23824369 10.1128/CMR.00097-12 PMC3719499

[30] Darricarrere N Broad neutralization of H1 and H3 viruses by adjuvanted influenza HA stem vaccines in nonhuman primates. Sci. Transl. Med 13, eabe5449 (2021). 10.1126/scitranslmed.abe5449 33658355

[31] Krammer F, Garcia-Sastre A & Palese P Is it possible to develop a ‘universal’ influenza virus vaccine? Potential target antigens and critical aspects for a universal influenza vaccine. Cold Spring Harb. Perspect. Biol 10, a028845 (2018). 28663209 10.1101/cshperspect.a028845 PMC6028071

[32] Ng S Novel correlates of protection against pandemic H1N1 influenza A virus infection. Nat. Med 25, 962–967 (2019). 31160818 10.1038/s41591-019-0463-x PMC6608747

[33] Wu NC & Wilson IA Structural insights into the design of novel anti-influenza therapies. Nat. Struct. Mol. Biol 25, 115–121 (2018). 29396418 10.1038/s41594-018-0025-9 PMC5930012

[34] Nachbagauer R A chimeric hemagglutinin-based universal influenza virus vaccine approach induces broad and long-lasting immunity in a randomized, placebo-controlled phase I trial. Nat. Med 26, 106–114 (2020). 10.1038/s41591-020-1118-7 33288923

[35] Bernstein DI Immunogenicity of chimeric haemagglutinin-based, universal influenza virus vaccine candidates: interim results of a randomised, placebo-controlled, phase 1 clinical trial. Lancet Infect. Dis 20, 80–91 (2020). 31630990 10.1016/S1473-3099(19)30393-7 PMC6928577

[36] Andrews SF, Graham BS, Mascola JR & McDermott AB Is it possible to develop a ‘universal’ influenza virus vaccine? Immunogenetic considerations underlying B-cell biology in the development of a pan-subtype influenza A vaccine targeting the hemagglutinin stem. Cold Spring Harb. Perspect. Biol 10, a029413 (2018). 28663207 10.1101/cshperspect.a029413 PMC6028068

[37] Stadlbauer D Vaccination with a recombinant H7 hemagglutinin-based influenza virus vaccine induces broadly reactive antibodies in humans. mSphere 2, e00502–e00517 (2017). 29242836 10.1128/mSphere.00502-17 PMC5729220

[38] Nachbagauer R Hemagglutinin stalk immunity reduces influenza virus replication and transmission in ferrets. J. Virol 90, 3268–3273 (2015). 26719251 10.1128/JVI.02481-15 PMC4810634

[39] Ellebedy AH Induction of broadly cross-reactive antibody responses to the influenza HA stem region following H5N1 vaccination in humans. Proc. Natl Acad. Sci. USA 111, 13133–13138 (2014). 25157133 10.1073/pnas.1414070111 PMC4246941

[40] Kanekiyo M & Graham BS Next-generation influenza vaccines. Cold Spring Harb. Perspect. Med 11, a038448 (2020). 10.1101/cshperspect.a038448 PMC8327825 32229612

