# Supplementary material for: Safety and immunogenicity of a ferritin nanoparticle H2 influenza vaccine in healthy adults: a phase 1 trial
Source: Nat Med. Author manuscript; Available in PMC 2023 Oct 20. (PMC10588819; doi:10.1038/s41591-021-01660-8)
Supplement: Supplementary Materials [file NIHMS1931196-supplement-Supplementary_Materials.pdf]

nature portfolio

https://doi.org/10.1038/s41591-021-01660-8

Supplementary information

Safety and immunogenicity of a ferritin nanoparticle H2 influenza vaccine in healthy adults: a phase 1 trial

In the format provided by the authors and unedited

Supplementary Appendix

This appendix has been provided by the authors to give readers additional information about their work.

Supplement to: Houser and Chen et al. Safety and immunogenicity of a ferritin nanoparticle H2 influenza vaccine in healthy adults: a phase 1 trial

Table of Contents

Supplemental Methods	3-9
Table S1	10
Table S2	11
Table S3	12
Table S4	13
Table S5	14
Table S6	15
Table S7	16
References	17

Supplemental Methods

Sex, Number, and Age of Analyzed Participants

Fifty healthy adults were enrolled into the trial. The 25 male and 25 female participants had median ages of 34 years (H2-naïve adults) and 59 years (H2-exposed adults). Participants who had altered or discontinued vaccination schedules were monitored for safety and were included in the immunogenicity analysis until their vaccination schedules were changed.

Details on the Complete Immunogenicity Analysis Set

For all experiments analyzing the single H2HA-Ferritin group we tested samples for 4 male and 1 female participants with a median age of 31 years. For all other groups at baseline and week four timepoints, we analyzed four 7 male and 8 female participants with a median age of 33 years (H2-naïve adults, homologous prime-boost), 5 male and 5 female participants with a median age of 35 years (H2-naïve adults, heterologous prime-boost), 3 male and 7 female participants with a median age of 59 years (H2-exposed adults, homologous prime-boost), and 6 male and 4 female participants with a median age of 59 years (H2-exposed adults, heterologous prime-boost). At week 18 we analyzed samples for 7 male and 6 female participants with a median age of 32 years (H2-naïve adults, homologous prime-boost), 4 male and 4 female participants with a median age of 36 years (H2-naïve adults, heterologous prime-boost), 3 male and 6 female participants with a median age of 59 years (H2-exposed adults, homologous prime-boost), and 5 male and 4 female participants with a median age of 58 years (H2-exposed adults, heterologous prime-boost). For HA ELISA and reporter microneutralization

assays, at week 16 we analyzed samples for 7 male and 7 female participants with a median age of 32 years (H2-naïve adults, homologous prime-boost), 4 male and 4 female participants with a median age of 36 years (H2-naïve adults, heterologous prime-boost), 3 male and 6 female participants with a median age of 59 years (H2-exposed adults, homologous prime-boost), and 5 male and 4 female participants with a median age of 58 years (H2-exposed adults, heterologous prime-boost). For the anti-ferritin response analysis, at week 20 we analyzed samples for 7 male and 6 female participants with a median age of 32 years (H2-naïve adults, homologous prime-boost), 4 male and 4 female participants with a median age of 36 years (H2-naïve adults, heterologous prime-boost), 3 male and 6 female participants with a median age of 59 years (H2-exposed adults, homologous prime-boost), and 5 male and 4 female participants with a median age of 58 years (H2-exposed adults, heterologous prime-boost). At weeks 28 and 40 we analyzed samples for 6 male and 6 female participants with a median age of 32 years (H2-naïve adults, homologous prime-boost), 4 male and 4 female participants with a median age of 36 years (H2-naïve adults, heterologous prime-boost), 3 male and 6 female participants with a median age of 59 years (H2-exposed adults, homologous prime-boost), and 5 male and 4 female participants with a median age of 58 years (H2-exposed adults, heterologous prime-boost). For a summary of the number of participants analyzed in all assays, see Table S7.

Immunological assays

Hemagglutination inhibition assays (HAI) against A/Singapore/1/1957 (H2N2) were performed at Southern Research, Inc. (Birmingham, AL) using validated methods as previously described.[1]

Briefly, the HAI assays were performed in duplicate in V-bottom plates using 0.5% horse red blood cells and four HA units of virus.

Serum samples were analyzed for binding to the HA stem antigens from both influenza group 1 and group 2 viruses by Meso Scale Discovery (MSD) Electro-ChemiLuminescence Immuno Assay (ECLIA). MSD 384 well Streptavidin coated SECTOR® Imager S 600 Reader Plates were blocked with 5% MSD Blocker A for 30-60 minutes, then washed three times with wash buffer (PBS and 0.05% Tween). The plates were then coated with 1 ug/mL biotinylated HA full-length (FL) or stabilized stem (ss) proteins (H2ss: A/Singapore/1/57, H5ss: A/Indonesia/5/05, H6 FL*: A/Taiwan/2/2013, H1ss: A/New Caledonia/20/99, or H7ss: A/Shanghai/2/2013) for one hour and washed. Serum was diluted 1:100 in 1% MSD Blocker A, serially diluted 3-fold for eight dilutions, and added to the coated plates. After a one hour incubation, plates were washed and incubated for an additional hour with 0.5ug/ml (1:2000 dilution) of anti-human IgG, IgM, IgA (H+L) secondary antibody (ThermoFisher Cat#31128, lot #0031128), which had been SULFO-TAG conjugated using the manufacturer's guidelines for MSD GOLD SULFO-TAG NHS-Ester Conjugation (MSD, cat #R91AO-1). After washing, the plates were read using 1X MSD Read Buffer (MSD, cat #R92TC-1) using an MSD SECTOR® Imager S 600. Binding curves were plotted, and geometric mean calculated using Prism (GraphPad, San Diego, CA). *The full-length antigen was used for the H6 virus since a stabilized stem version was unavailable. However, since the H6 subtype is antigenically distinct, the majority of binding should occur on the conserved regions, including the HA stem.

For the reporter-based microneutralization assay, replication-restricted reporter (R3) viruses where the viral genomic RNA encoding either the HA (R3ΔHA) (H2N2: A/Singapore/1/57, H5N1: A/Vietnam/1203/04), or PB1 (R3ΔPB1) (H6N1: A/Taiwan/2/2013) were replaced with a fluorescent gene TdKatushka2 as previously described.[2,3] The R3ΔHA viruses were rescued by reverse genetics and propagated in MDCK-SIAT1 cells expressing the corresponding HA protein and the R3ΔPB1 virus was rescued by reverse genetics and propagated in MDCK-SIAT1 cells expressing the PB1 protein. Viruses were titrated by counting fluorescent foci with a Celigo Image Cytometer (Celigo v4.1, Nexcelom) with a customized filter to detect TdKatushka2. Four-fold serial dilutions of human sera, starting at 1:40 with seven dilutions, were pretreated with receptor destroying enzyme II (RDE) (Denka Seiken, Japan) and heat inactivated at 56°C for 40 minutes, then mixed 1:1 with R3 viruses ($8x10^4$ foci/mL) for 1 hour at 37°C. The mixture was then added to cells in quadruplicate in a 384 well plate for 18-22 hours at 37°C. The percent neutralization was calculated by constraining the virus control at 0% and the cell-only control at 100% and plotting against serum concentration. A curve fit was generated by a four-parameter nonlinear fit model in Prism (GraphPad, San Diego, CA). The 80% (IC_{80}) inhibitory concentrations were obtained from the curve fit for each serum sample. Competition microneutralization assays were performed in similar fashion as described above, with the exception of a serum pre-incubation step with appropriate competitor proteins (50 µg/ml) for 1 hour prior to being mixed with virus. The assays were performed with a full dilution series (four fold dilutions starting at 1:40). Competitor proteins used for the assay were H2 (A/Singapore/1/1957) HA ectodomain trimer, H2 stabilized stem HA trimer,[4] and a control DS-Cav1 (respiratory syncytial virus stabilized fusion protein).[5]

For the pseudotyped lentivirus neutralization assay, influenza HA pseudotyped lentiviral vectors expressing a luciferase reporter gene were produced as previously described.[6] 293T cells were transfected with the following plasmids: pCMVΔR8.2, pHR'CMV-Luc, pCMV Sport/h TMPRSS2, and the HA and NA from H2N2 A/Singapore/1/1957 or H5N1 A/Vietnam/1203/2004. Serial three-fold dilutions of human sera, starting at 1:50 with seven dilution points, were pretreated with RDE were then mixed with pseudoviruses, incubated for 45 min at 37°C, and added to 96-well white/black isoplates (PerkinElmer, Walthan, MA) in triplicate. 293A cells were added at $1x10^4$ cells/well. After two hours, wells were replenished with fresh media. Cells were lysed 72 hours later with cell culture lysis buffer (Promega, Madison, WI) and luciferase assay reagent (Promega, Madison, WI) was added to each well. Luciferase activity was measured according to relative luciferase units by Microbeta luminescence counter (PerkinElmer, Waltham, MA). IC_{80} titers were generated using Prism (GraphPad, San Diego, CA), and are the average of two separate experiments.

An antibody dependent cell-mediated cytotoxicity (ADCC) reporter assay (Promega) was used to screen the serum samples for ADCC activity at four weeks after each vaccination. A549 cells were infected in 96 well plates at an MOI of 5 using a recombinant chimeric influenza virus, in the absence of TPCK-trypsin to prevent multi-cycle growth. The chimeric virus used contained a chimeric HA with the head from an H6 subtype (A/mallard/Sweden/81/2002) and the stalk from an H1 subtype (A/California/04/2009) with a neuraminidase from an N5 strain (A/mallard/Sweden/86/2003) on an A/Puerto Rico/8/1934 backbone. The exotic HA head and

NA proteins, to which adults are typically naïve, allow for the assay to predominantly measure responses against conserved epitopes. After 24 hours, serum samples were analyzed with a starting dilution of 1:50 and diluted 1:3 with a five point curve before being added to infected cells. Effector cells (Jurkat cells expressing human FcγRIIIa, Promega) were added at $5x10^4$ cells/well and plates were incubated for 6 hours. Bio-Glo Luciferase substrate (Promega) was added to each well and luciferase was measured using a Synergy H1 hybrid multimode microplate reader (BioTek). The value of each well was calculated as the percent positive compared to the assay positive control (mAb CR9114, cloned and produced at Mt. Sinai)[7] starting at 30 μg/mL. A nonlinear regression line was fit using Prism (GraphPad, San Diego, CA) to determine the EC_{50} values for the serum samples.

All serum samples were evaluated for the presence of antibodies against human ferritin: both heavy chain (VRC-VPP, Lot# LW519-189-01 2/13/17) and light chain (LifeSpan Biologicals Cat #LS-G3413, Lot #109413), and H. pylori ferritin (Master stock #189075 from VRC Lot KL596-14-04; VRC plasmid 3374) using validated methods. Four non-human primate (NHPs, cynomolgus macaques) sera were included in the H. pylori analysis as positive controls. These NHPs were vaccinated with 50 μg of an influenza ferritin vaccine at weeks 0, 4, and 10. Pre-vaccination and post vaccination (week 12) samples were included in the analysis. For the ELISA assay, purified recombinant ferritin was coated on 96-well Immulon2 plates (Thermo scientific, Cat #3655) and incubated overnight at 2-8°C. Anti-ferritin titers were determined using sequential incubation with biotin-labeled anti-human IgG, IgA, or IgM (1:12,800 dilution, KPL, Cat# 16-10-07) for the human heavy and light chains, or biotin-labeled mouse anti-pan-primate IgG (1:16,000 dilution,

Absolute Antibody Cat# Ab01271-1.1, clone 8F1) for *H. pylori.,* followed by the addition of Streptavidin conjugated with horseradish peroxidase (SeraCare Cat #5270-0029) and TMB (3, 5', 5,5'-tetra-methylbenzidine) substrate (KPL, Cat. No. 52-00-02). Color development was stopped by the addition of sulfuric acid (Sigma, Cat # 258105) and the plates were read using a Spectramax microplate spectrophotometer (Molecular Devices). The titers were presented as optical density at 450nm.

Table S1. Summary of Participant Demographic Data

	Single H2HA-Ferritin (n=5)	*H2-naïve Homologous Prime-Boost (n=15)*	*H2-naïve Heterologous Prime-Boost (n=10)*	**H2-exposed Homologous Prime-Boost** *(n=10)*	*H2-exposed Heterologous Prime-Boost (n=10)*	*Overall (n=50)*
Sex — no.(%)						
Male	4 (80%)	7 (47%)	5 (50%)	3 (30%)	6 (60%)	25 (50%)
Female	1 (20%)	8 (53%)	5 (50%)	7 (70%)	4 (40%)	25 (50%)
Age – median yr [range]	31 [22-37]	33 [22-46]	35 [19-47]	59 [52-67]	59 [52-67]	43 [19-67]
Race — no. (%)[a]						
Asian	1 (20%)	2 (13%)	1 (10%)	0 (0%)	0 (0%)	4 (8%)
Black or African American	0 (0%)	3 (20%)	0 (0%)	5 (50%)	1 (10%)	9 (18%)
Native Hawaiian or other Pacific Islander	0 (0%)	0 (0%)	0 (0%)	0 (0%)	1 (10%)	1 (2%)
White	4 (80%)	10 (67%)	7 (70%)	5 (50%)	7 (70%)	33 (66%)
Multiracial	0 (0%)	0 (0%)	2 (20%)	0 (0%)	1 (10%)	3 (6%)
Hispanic or Latino ethnic group — no. (%)[a]						
Non-Hispanic or Latino	5 (100%)	14 (93%)	8 (80%)	10 (100%)	9 (90%)	46 (92%)
Hispanic or Latino	0 (0%)	1 (7%)	2 (20%)	0 (0%)	1 (10%)	4 (8.0%)
Body-mass index — mean (SD)[b]	24 (2)	25 (5)	27 (3)	27 (3)	28 (5)	26 (4)
Educational level — no. (%)						
High school graduate/GED	0 (0%)	0 (0%)	0 (0%)	2 (20%)	1 (10%)	3 (6.0%)
College/University	2 (40%)	4 (27%)	4 (40%)	4 (40%)	4 (40%)	18 (36%)
Advanced degree	3 (60%)	11 (73%)	6 (60%)	4 (40%)	5 (50%)	29 (58%)

[a]Race and ethnic group were reported by the participants.

[b]The body-mass index is the weight in kilograms divided by the square of the height in meters. This calculation was performed on the basis of weight and height measured at the time of enrollment.

Table S2. Influenza history for participants prior to the clinical trial, and reports of influenza-like illness during the trial.

	Single H2HA-Ferritin (n=5)	*H2-naïve Homologous Prime-Boost (n=15)*	*H2-naïve Heterologous Prime-Boost (n=10)*	*H2-exposed Homologous Prime-Boost (n=10)*	*H2-exposed Heterologous Prime-Boost (n=10)*	*Overall (n=50)*
Frequency of influenza vaccination in the previous 5 years — no.(%)						
1-2 times	0 (0%)	3 (20%)	1 (10%)	2 (20%)	3 (30%)	9 (18%)
3-5 times	5 (100%)	10 (67%)	5 (50%)	5 (50%)	3 (30%)	28 (56%)
>5 times	0 (0%)	2 (13%)	4 (40%)	3 (30%)	4 (40%)	13 (26%)
Most recent influenza vaccine — no.(%)						
2014/2015	0 (0%)	0 (0%)	0 (0%)	0 (0%)	0 (0%)	0 (0%)
2015/2016	1 (20%)	1 (7%)	0 (0%)	1 (10%)	0 (0%)	3 (6%)
2016/2017	2 (40%)	2 (13%)	0 (0%)	2 (20%)	3 (30%)	9 (18%)
2017/2018	2 (40%)	12 (80%)	10 (100%)	7 (70%)	6 (60%)	37 (74%)
2018/2019	0 (0%)	0 (0%)	0 (0%)	0 (0%)	1 (10%)	1 (2%)
Influenza-like illness in the previous 12 months — no.(%)						
Yes	1 (20%)	3 (20%)	3 (30%)	2 (20%)	0 (0%)	9 (18%)
No	4 (80%)	12 (80%)	7 (70%)	8 (80%)	10 (100%)	41 (82%)
Received influenza vaccine while on study — no.(%)						
Yes	1 (20%)	6 (40%)	8 (80%)	8 (80%)	5 (50%)	28 (56%)
No	4 (80%)	9 (60%)	2 (20%)	2 (20%)	5 (50%)	22 (44%)
Influenza-like illnesses reported while on study — no.(%)						
Yes	0 (0%)	1 (7%)	1 (10%)	0 (0%)	0 (0%)	2 (4%)
No	5 (100%)	14 (93%)	9 (90%)	10 (100%)	10 (100%)	48 (96%)

Table S3. Local and systemic reactogenicity following both H2HA-Ferritin and H2 DNA vaccinations.

	Prime Vaccine					Boost Vaccine			
	H2HA-Ferritin			H2 DNA		H2HA-Ferritin			
Symptoms Intensity	*Single dose (n=5)*	*H2-naive Homologous prime-boost (n=15)*	*H2-exposed Homologous prime-boost (n=10)*	*H2-naïve Heterologous prime-boost (n=10)*	*H2-exposed Heterologous prime-boost (n=10)*	*H2-naive Homologous prime-boost (n=13)*	*H2-exposed Homologous prime-boost (n=9)*	*H2-naïve Heterologous prime-boost (n=8)*	*H2-exposed Heterologous prime-boost (n=9)*
**PAIN/TENDERNESS**									
**None**	4 (80%)	14 (93%)	9 (90%)	4 (40%)	4 (40%)	10 (77%)	9 (100%)	7 (88%)	9 (100%)
**Mild**	1 (20%)	1 (7%)	1 (10%)	6 (60%)	6 (60%)	3 (23%)	0 (0%)	1 (12%)	0 (0%)
**SWELLING**									
**None**	5 (100%)	15 (100%)	10 (100%)	10 (100%)	10 (100%)	13 (100%)	9 (100%)	8 (100%)	9 (100%)
**REDNESS**									
**None**	5 (100%)	15 (100%)	10 (100%)	10 (100%)	10 (100%)	13 (100%)	9 (100%)	8 (100%)	9 (100%)
**ANY LOCAL SYMPTOM**									
**None**	4 (80%)	14 (93%)	9 (90%)	4 (40%)	4 (40%)	10 (77%)	9 (100%)	7 (88%)	9 (100%)
**Mild**	1 (20%)	1 (7%)	1 (10%)	6 (60%)	6 (60%)	3 (23%)	0 (0%)	1 (12%)	0 (0%)
**MALAISE**									
**None**	4 (80%)	13 (87%)	9 (90%)	9 (90%)	8 (80%)	10 (77%)	8 (89%)	7 (88%)	7 (78%)
**Mild**	1 (20%)	2 (13%)	1 (10%)	1 (10%)	2 (20%)	3 (23%)	1 (11%)	1 (12%)	2 (22%)
**MYALGIA**									
**None**	5 (100%)	11 (73%)	8 (80%)	9 (90%)	8 (80%)	12 (92%)	9 (100%)	8 (100%)	7 (78%)
**Mild**	0 (0%)	3 (20%)	2 (20%)	1 (10%)	2 (20%)	1 (8%)	0 (0%)	0 (0%)	2 (22%)
**Moderate**	0 (0%)	1 (7%)	0 (0%)	0 (0%)	0 (0%)	0 (0%)	0 (0%)	0 (0%)	0 (0%)
**HEADACHE**									
**None**	4 (80%)	10 (67%)	8 (80%)	8 (80%)	7 (70%)	10 (77%)	8 (89%)	8 (100%)	6 (67%)
**Mild**	1 (20%)	5 (33%)	2 (20%)	2 (20%)	3 (30%)	3 (23%)	1 (11%)	0 (0%)	3 (33%)
**CHILLS**									
**None**	5 (100%)	14 (93%)	9 (90%)	10 (100%)	10 (100%)	13 (100%)	9 (100%)	8 (100%)	9 (100%)
**Mild**	0 (0%)	1 (7%)	1 (10%)	0 (0%)	0 (0%)	0 (0%)	0 (0%)	0 (0%)	0 (0%)
**NAUSEA**									
**None**	5 (100%)	15 (100%)	9 (90%)	10 (100%)	10 (100%)	15 (100%)	9 (100%)	8 (100%)	9 (100%)
**Mild**	0 (0%)	0 (0%)	1 (10%)	0 (0%)	0 (0%)	0 (0%)	0 (0%)	0 (0%)	0 (0%)
**JOINT PAIN**									
**None**	5 (100%)	14 (93%)	9 (90%)	10 (100%)	10 (100%)	15 (100%)	9 (100%)	8 (100%)	9 (100%)
**Mild**	0 (0%)	1 (7%)	1 (10%)	0 (0%)	0 (0%)	0 (0%)	0 (0%)	0 (0%)	0 (0%)
**FEVER[a]**									
**None**	5 (100%)	14 (93%)	10 (100%)	10 (100%)	10 (100%)	15 (100%)	9 (100%)	8 (100%)	9 (100%)
**Mild**	0 (0%)	0 (0%)	0 (0%	0 (0%)	0 (0%)	0 (0%)	0 (0%)	0 (0%)	0 (0%)
**Moderate**	0 (0%)	0 (0%)	0 (0%)	0 (0%)	0 (0%)	0 (0%)	0 (0%)	0 (0%)	0 (0%)
**Severe**	0 (0%)	1 (4%)	0 (0%)	0 (0%)	0 (0%)	0 (0%)	0 (0%)	0 (0%)	0 (0%)
**ANY SYSTEMIC SYMPTOM**									
**None**	3 (60%)	9 (60%)	7 (70%)	7 (70%)	6 (60%)	7 (54%)	8 (89%)	7 (88%)	5 (56%)
**Mild**	2 (40%)	4 (26%)	3 (30%)	3 (30%)	4 (40%)	6 (46%)	1 (11%)	1 (12%)	4 (44%)
**Moderate**	0 (0%)	1 (7%)	0 (0%)	0 (0%)	0 (0%)	0 (0%)	0 (0%)	0 (0%)	0 (0%)
**Severe**	0 (0%)	1 (7%)	0 (0%)	0 (0%)	0 (0%)	0 (0%)	0 (0%)	0 (0%)	0 (0%)

For participants reporting a symptom on multiple days, the symptom is counted once at the maximum severity.

N= number of participants per group

[a]Severe fever is defined as a temperature between 39-40°C (102.1-104°F)

Table S4. Related Adverse Events (AEs) in participants following vaccination.

Participant	Vaccination Group	Related AE	Severity[a]	Vaccine	Vaccination	Days Post Vaccination	Time to resolution
5	Single H2HA-Ferritin	Leukocytosis	Mild	20 µg H2HA-Ferritin	Prime	16	12 days
13	H2-naïve Homologous Prime-Boost	Leukopenia	Mild	60 µg H2HA-Ferritin	Prime	16	50 days
23	H2-naïve Heterologous Prime-boost	Neutropenia	Moderate	H2 DNA	Prime	14	16 days
34	H2-exposed Homologous Prime-Boost	Alkaline Phosphatase Elevation	Mild	60 µg H2HA-Ferritin	Prime	14	15 days
41	H2-exposed Heterologous Prime-Boost	Leukopenia	Mild	H2 DNA	Prime	28	36 days
45	H2-exposed Heterologous Prime-Boost	Neutropenia	Mild	H2 DNA	Prime	14	15 days

[a]Mild severity indicates no effect on activities of daily living, and moderate severity indicates some interference with activity not requiring medical intervention.

Table S5. Durability of vaccine-induced antibody responses determined by significant increases in antibody levels at six months post boost compared to baseline levels, reported by p values.

Vaccination Regimen	HA Stem-Binding ECLIA (Figure 4)					Reporter Microneutralization Assay (Figure 5)	
	**H2ss**	**H5ss**	**H6FL**	**H1ss**	**H7ss**	**H2N2**	**H5N1**
**H2-naïve Homologous Prime Boost (n=12)**	**0.005**	**0.02**	**0.004**	0.32	0.09	**<0.001**	**0.001**
**H2-naïve Heterologous Prime Boost (n=8)**	**<0.001**	**<0.001**	**<0.001**	**<0.001**	0.05	**<0.001**	**<0.001**
**H2-exposed Homologous Prime Boost (n=9)**	0.64	0.52	0.06	0.2	0.67	**<0.001**	**0.02**
**H2-exposed Heterologous Prime Boost (n=9)**	**0.03**	0.97	**0.004**	0.76	0.16	**0.002**	**0.006**

Comparisons between treatment groups were made using two-sided t-tests with no adjustments for multiple comparisons, after $\log_{10}$ transformations of the raw titers (for the neutralization assays).

Table S6.H2HA-Ferritin vaccination induces broadly neutralizing antibodies against H6N1 in H2-naïve adults.

Vaccine Regimen	A/Taiwan/2/2013 (H6N1)		
	Micro-neutralization IC_{80} GMT (95% CI)		Geometric mean fold increase from baseline
	Baseline	Week 18	
**H2-naïve**			
**Homologous Prime-Boost (n=13)**	1.76 (1.58-1.95)	1.94 (1.64-2.24)	1.41
**Heterologous Prime-Boost (n=8)**	1.64 (1.57-1.72)	2.02 (1.77-2.27)	2.38
**H2-exposed**			
**Homologous Prime-Boost (n=9)**	1.63 (1.56-1.70)	1.39 (NA)[a]	0.93
**Heterologous Prime-Boost (n=9)**	1.64 (1.57-1.73)	1.67 (1.52-1.83)	1.05

[a]NA= neutralizing titer in serum of all group participants was below the limit of detection for the assay.

Table S7. Number of participant samples in each vaccination group analyzed at each timepoint, for all immunological assays

Vaccination Group	Weeks after first vaccination						
	**0[a]**	**4**	**16[a]**	**18**	**20**	**28**	**40**
**Single H2HA-Ferritin vaccination**	5	5	5	5	5	5	5
**H2-naïve homologous prime-boost**	15	15	14	13	13	12	12
**H2-naïve heterolgous prime-boost**	10	10	8	8	8	8	8
**H2-exposed homologous prime-boost**	10	10	9	9	9	9	9
**H2-exposed heterologous prime-boost**	10	10	9	9	9	9	9

[a]Weeks 0 and 16 represent the vaccination time points for the trial.

References

1. Houser, K.V., *et al.* DNA vaccine priming for seasonal influenza vaccine in children and adolescents 6 to 17 years of age: A phase 1 randomized clinical trial. *PLoS One* **13**, e0206837 (2018).
2. Creanga, A., *et al.* A comprehensive influenza reporter virus panel for high-throughput deep profiling of neutralizing antibodies. *Nat Commun* **12**, 1722 (2021).
3. Shcherbo, D., *et al.* Far-red fluorescent tags for protein imaging in living tissues. *Biochem J* **418**, 567-574 (2009).
4. Yassine, H.M., *et al.* Use of Hemagglutinin Stem Probes Demonstrate Prevalence of Broadly Reactive Group 1 Influenza Antibodies in Human Sera. *Sci Rep* **8**, 8628 (2018).
5. McLellan, J.S., *et al.* Structure of RSV fusion glycoprotein trimer bound to a prefusion-specific neutralizing antibody. *Science* **340**, 1113-1117 (2013).
6. Yang, Z.Y., *et al.* Selective modification of variable loops alters tropism and enhances immunogenicity of human immunodeficiency virus type 1 envelope. *J Virol* **78**, 4029-4036 (2004).
7. Chromikova, V., Zaragoza, M.A. & Krammer, F. Generation of a serum free CHO DG44 cell line stably producing a broadly protective anti-influenza virus monoclonal antibody. *PLoS One* **12**, e0183315 (2017).

Official Title: A Phase I Open-Label Clinical Trial to Evaluate Dose, Safety, Tolerability, and Immunogenicity of an Influenza HA Ferritin Vaccine Alone or in Prime-Boost Regimen with an Influenza DNA Vaccine in Healthy Adults

ClinicalTrials.gov Identifier: NCT03186781

Document Type:

- Redacted Final Version of the Study Protocol (Statistical Analysis Considerations located in Section 6 of the Protocol)
- Redacted Final Version of IRB-approved Informed Consents

Document/Date:

- Protocol Version 6.0: March 11, 2019
- Version 5.0 IRB-approved Informed Consents – Main and Supplemental: November 13, 2018

Version 6.0
March 11, 2019

VACCINE RESEARCH CENTER

Protocol VRC 316

(NIH 17-I-0110)

A PHASE I OPEN-LABEL CLINICAL TRIAL TO EVALUATE DOSE, SAFETY, TOLERABILITY, AND IMMUNOGENICITY OF AN INFLUENZA HA FERRITIN VACCINE, ALONE OR IN PRIME-BOOST REGIMENS WITH AN INFLUENZA DNA VACCINE IN HEALTHY ADULTS

Vaccine Provided by
National Institute of Allergy and Infectious Diseases (NIAID)
Vaccine Research Center (VRC)
Bethesda, Maryland

Clinical Trial Sponsored by:
National Institute of Allergy and Infectious Diseases (NIAID)
Vaccine Research Center (VRC)
Bethesda, Maryland

BB-IND 17399 – held by VRC, NIAID, NIH

Principal Investigator:
Grace Chen, M.D., M.P.H
Vaccine Research Center, National Institute of Allergy and Infectious Diseases (NIAID)
National Institutes of Health (NIH)
Bethesda, MD 20892

IRB Initial Review Date: March 13, 2016

TABLE OF CONTENTS

TABLE OF ABBREVIATIONS **6**

PRÉCIS **8**

1. INTRODUCTION AND RATIONALE **10**

1.1 BACKGROUND 10

1.2 Rationale For Development Of Influenza HA-F A/Sing Vaccine, VRC-FLUNPF081-00-VP 11

1.3 Rationale For Development of Influenza Plasmid DNA A/Sing Vaccine, VRC-FLUDNA082-00-VP and its use with VRC-FLUNPF081-00-VP in Prime-Boost Regimens 11

1.3.1 DNA vaccines developed by VRC, NIAID, NIH *12*

1.4 Rationale for VRC-FLUDNA082-00-VP Vaccine Administration Method 12

1.5 Rationale For Study Population Proposed In The Clinical Trial 13

1.6 Assessment of Vaccine Immunogenicity 13

2. STUDY AGENTS **14**

2.1 VRC-FLUNPF081-00-VP 14

2.2 VRC-FLUDNA082-00-VP 14

2.3 VRC-PBSPLA043-00-VP, PHOSPHATE BUFFERED SALINE (DILUENT) 14

2.4 Preclinical Studies with VRC-FLUNPF081-00-VP 14

2.5 Preclinical Studies with VRC-FLUDNA082-00-VP 15

3. STUDY OBJECTIVES **15**

3.1 Primary Objectives 15

3.2 Secondary Objectives 15

3.3 Exploratory Objectives 16

4. STUDY DESIGN AND METHODS **16**

4.1 Study Population 16

4.1.1 Inclusion Criteria *16*

4.1.2 Exclusion Criteria *17*

4.2 Clinical Procedures and Evaluations 19

4.2.1 Screening *19*

4.2.2 Study Schedule *19*

4.2.3 Enrollment and Randomization *20*

4.2.4 Administration of Study Vaccines *20*

4.2.5 7-Day Solicited Reactogenicity and Follow-up *21*

4.2.6 Follow-Up through End of Study *21*

4.2.7 Blood Sample Collection *21*

4.2.8 Apheresis *21*

4.2.9 Concomitant Medications ... *22*
4.3 Criteria for Dose Escalation and Dose Continuation ... 22
4.4 Criteria for Discontinuing Study Injections or Protocol Participation ... 23
4.4.1 Discontinuation of Study Injections ... *23*
4.4.2 Discontinuation of Protocol Participation ... *24*
4.5 Criteria for Pausing and Resuming the Study ... 24
4.5.1 Plan for Pausing the Study ... *24*
4.5.2 Plan for Review of Pauses and Resuming the Study ... *24*
5. SAFETY AND ADVERSE EVENT REPORTING ... 24
5.1 Adverse Events ... 24
5.2 Serious Adverse Events ... 25
5.3 Adverse Event Reporting to the IND Sponsor ... 25
5.3.1 IND Sponsor Reporting to the FDA ... *26*
5.4 Reporting to the Institutional Review Board ... 26
5.4.1 Unanticipated Problem Definition ... *26*
5.4.2 Protocol Deviation Definition ... *27*
5.4.3 Non-Compliance Definition ... *27*
5.4.4 Expedited Reporting to the NIAID IRB ... *27*
5.4.5 Annual Reporting to the NIAID IRB ... *28*
5.5 Serious Adverse Event Reporting to the Institutional Biosafety Committee (IBC) ... 28
6. STATISTICAL CONSIDERATIONS ... 28
6.1 Overview ... 28
6.2 Objectives ... 28
6.3 Endpoints ... 29
6.3.1 Primary Endpoints: Safety ... *29*
6.3.2 Secondary Endpoints: Immunogenicity ... *29*
6.3.3 Exploratory Endpoints: Immunogenicity ... *29*
6.4 Sample Size and Accrual ... 29
6.4.1 Power Calculations for Safety ... *29*
6.4.2 Power Calculations for Immunogenicity ... *31*
6.4.3 Power for Comparison ... *31*
6.5 Statistical Analysis ... 32
6.5.1 Analysis Variables ... *32*
6.5.2 Baseline Demographics ... *32*
6.5.3 Safety Analysis ... *33*

6.5.4 Immunogenicity Analysis *33*

6.5.5 Missing Data *33*

6.5.6 Interim Analyses *34*

6.6 Intervention Assignments 34

7. PHARMACY AND VACCINE ADMINISTRATION PROCEDURES 34

7.1 Study Product 34

7.2 Study Product Presentation and Storage 35

7.2.1 Study Product Labels *35*

7.2.2 Study Product Storage *35*

7.2.3 Temperature excursions *35*

7.3 Preparation of Study Products for Injection 35

7.3.1 Preparation of VRC-FLUNPF081-00-VP to be administered via Needle and Syringe Injection *36*

7.3.2 Preparation of VRC-FLUDNA082-00-VP to be administered via PharmaJet Injection .. *36*

7.4 Study Product Accountability 36

7.4.1 Documentation *36*

7.4.2 Disposition *36*

8. HUMAN SUBJECT PROTECTIONS AND ETHICAL OBLIGATIONS 36

8.1 Institutional Review Board 37

8.2 Subject Identification and Enrollment of Study Participants 37

8.2.1 Participation of Children *37*

8.2.2 Participation of NIH Employees *37*

8.3 Informed Consent 37

8.4 Subject Confidentiality 38

8.5 Risks and Benefits 38

8.5.1 Risks of the VRC-FLUNPF081-00-VP vaccine *38*

8.5.2 Risks of the VRC-FLUDNA082-00-VP Vaccine *38*

8.5.3 Other Risks *39*

8.5.4 Study Benefits *40*

8.6 Plan for Use and Storage of Biological Samples 40

8.6.1 Use of Samples, Specimens and Data *40*

8.6.2 Storage and Tracking of Blood Samples and Other Specimens *40*

8.6.3 Disposition of Samples, Specimens and Data at Completion of the Protocol *41*

8.6.4 Loss or Destruction of Samples, Specimens or Data *41*

8.7 Compensation 41

8.8 Safety Monitoring ... 42

8.8.1 Protocol Safety Review Team ... 42

9. ADMINISTRATIVE AND LEGAL OBLIGATIONS ... 42

9.1 Protocol Amendments and Study Termination ... 42

9.2 Study Documentation and Storage ... 42

9.3 Data Collection, Data Sharing and Protocol Monitoring ... 43

9.3.1 Data Collection ... 43

9.3.2 Data Sharing Plan ... 43

9.3.3 Source Documents ... 43

9.3.4 Protocol Monitoring Plan ... 43

9.4 Language ... 43

9.5 Policy Regarding Research-Related Injuries ... 44

10. REFERENCES ... 45

APPENDIX I: CONTACT INFORMATION ... 48

APPENDIX II: SCHEDULE OF EVALUATIONS ... 51

APPENDIX III: ASSESSMENT OF RELATIONSHIP TO VACCINE AND GRADING SEVERITY OF ADVERSE EVENTS ... 55

TABLE OF ABBREVIATIONS

Abbreviation	Term
AE	adverse event
AdEDC	Advantage Electronic Data Capture
ALT	alanine aminotransferase
AoU	assessment of understanding
BMI	body mass index
CBC	complete blood count
cGMP	current Good Manufacturing Practices
dbGAP	Database of Genotypes and Phenotypes
DNA	deoxyribonucleic acid
DTM	Department of Transfusion Medicine
FDA	Food and Drug Administration
GCP	Good Clinical Practices
GLP	Good Laboratory Practices
HA	influenza hemagglutinin protein
HAI	hemagglutination inhibition
HIV	human immunodeficiency virus
HLA	human leukocyte antigen
HRPP	Human Research Protection Program
IB	Investigator's Brochure
ICS	intracellular cytokine staining
IM	Intramuscular
IND	investigational new drug application
IRB	Institutional Review Board
LIMS	Laboratory Information Management System
MedDRA	Medical Dictionary for Regulatory Activities
MIV	Monovalent Inactivated Vaccine
NA	neuraminidase
NAb	Neutralizing antibody
NIAID	National Institute of Allergy and Infectious Diseases
NIH	National Institutes of Health
NIH CC	NIH Clinical Center
NSAID	nonsteroidal anti-inflammatory drug
PBMC	peripheral blood mononuclear cells
PI	Principal Investigator
PSRT	Protocol Safety Review Team
RNA	ribonucleic acid
SAE	serious adverse event
SARS	Severe acute respiratory syndrome
SUSAR	serious and unexpected suspected adverse reaction
TNF	tumor necrosis factors
ULN	upper limit of normal
UP	Unanticipated Problem
VCMP	Vaccine Clinical Materials Program

Abbreviation	Term
VITL	Vaccine Immunology Testing Laboratory
VRC	Vaccine Research Center
WBC	white blood cell
WHO	World Health Organization

PRÉCIS

VRC 316: A Phase I Open-Label Clinical Trial to Evaluate Dose, Safety, Tolerability, and Immunogenicity of an Influenza HA Ferritin Vaccine Alone or in Prime-Boost Regimen with an Influenza DNA Vaccine in Healthy Adults.

Study Design: This is a Phase I open-label, dose escalation study to evaluate the dose, safety, tolerability, and immunogenicity of VRC-FLUNPF081-00-VP (HA-F A/Sing) vaccine alone or in prime-boost regimens with VRC-FLUDNA082-00-VP (DNA A/Sing) vaccine. The hypotheses are that VRC-FLUNPF081-00-VP and VRC-FLUDNA082-00-VP vaccines are safe, well-tolerated, and induce an immune response to the H2 antigen. The primary objectives are to evaluate the safety and tolerability of two different doses of the HA-F A/Sing vaccine alone and in prime-boost regimens in healthy adults. Secondary objectives are related to the evaluation of the immunogenicity of the HA-F A/Sing and DNA A/Sing vaccines in prime-boost regimens.

Study Products: The investigational HA-F A/Sing vaccine, developed by the Vaccine Research Center (VRC), National Institute of Allergy and Infectious Diseases (NIAID), is composed of *Helicobacter pylori* non-haem ferritin with an influenza virus H2 hemagglutinin (HA) insert to form a nanoparticle displaying eight HA trimers from A/Singapore/1/57 (H2N2) influenza.

The investigational DNA A/Sing vaccine, developed by the VRC, NIAID, is composed of a single closed-circular DNA plasmid that encodes the H2 protein of A/Singapore/1/57 influenza.

Subjects: Up to 80 healthy adults ages 18-70 will be enrolled; adults born between 1966 and 1969 will be excluded from the trial.

Study Plan: Vaccines will be administered intramuscularly (IM) in the deltoid muscle. This study has two parts:

Part I will evaluate the safety, tolerability, and immunogenicity of 1 or 2 doses of the HA-F A/Sing vaccine in a dose-escalation design. In Group 1, five subjects will receive a low dose of the HA-F A/Sing vaccine via needle and syringe on Day 0. If this low dose is assessed as safe and well tolerated, enrollment will begin for Group 2. In Group 2, five subjects will receive the higher dose of the HA-F A/Sing vaccine via needle and syringe on Day 0 and Week 16. If this higher dose is assessed as safe, enrollment will begin for Part II.

Part II will evaluate the safety, tolerability, and immunogenicity of HA-F A/Sing vaccine in prime-boost regimens. Subjects will be stratified by age and randomized equally into Groups 3 and Group 4 as shown in the vaccination schema. In Group 3, subjects will receive DNA A/Sing vaccine via PharmaJet Needle-Free Injector on Day 0 and HA-F A/Sing vaccine via needle and syringe on Week 16. In Group 4, subjects will receive HA-F A/Sing vaccine via needle and syringe on Day 0 and Week 16.

For Group 1, the protocol requires about 8 clinic visits and 1 telephone follow-

up contact after the injection.
For Group 2, Group 3 and Group 4, the protocol requires about 10 clinic visits and 2 telephone follow-up contacts after each injection.
For all Groups, solicited reactogenicity will be evaluated using a 7-day diary card. Assessment of vaccine safety will include clinical observation and monitoring of hematological and chemical parameters at clinical visits throughout the study.

**VRC 316 Vaccination Schema**					
**PART I: Dose Escalation of HA-F A/Sing**					
**Group**	**Dose Level**	**Age Cohorts**	**Subjects**	**Day 0 Prime**	**Week 16 (±4 weeks) Boost**
1	Low dose	Ages 18-47	**5**	HA-F A/Sing, 20 mcg	
2	High dose	Ages 18-47	**5**	HA-F A/Sing, 60 mcg	HA-F A/Sing, 60 mcg
**Total**			**10***	*Enrollment of up to 15 subjects is permitted if additional subjects are needed for safety or immunogenicity evaluations.	
				HA-F A/Sing injections are administered by needle and syringe.	
**PART II: Evaluation of HA-F A/Sing and DNA A/Sing in Prime-Boost Regimens**					
**Group**	**Regimen**	**Age Cohorts**	**Subjects**	**Day 0 Prime**	**Week 16 (±4 weeks) Boost**
3	DNA A/Sing<br>HA-F A/Sing	Group 3A; 18-47	**10**	DNA A/Sing, 4 mg	HA-F A/Sing, 60 mcg
		Group 3B; 52-70	**10**		
4	HA-F A/Sing<br>HA-F A/Sing	Group 4A; 18-47	**10**	HA-F A/Sing, 60 mcg	HA-F A/Sing, 60 mcg
		Group 4B; 52-70	**10**		
**Total**			**40***	*Enrollment of up to 65 subjects is permitted if additional subjects are needed for safety or immunogenicity evaluations.	
				DNA A/Sing injections are administered by PharmaJet.<br>HA-F A/Sing injections are administered by needle and syringe.	

Study Duration: Subjects will be evaluated for 40 weeks following the first vaccine administration.

1. INTRODUCTION AND RATIONALE

1.1 BACKGROUND

Influenza is an enveloped, negative single-stranded (-ss) ribonucleic acid (RNA) virus that belongs to the family *Orthomyxoviridae*. Of the five genera of influenza circulating in nature, only influenza A and B are known to cause epidemics in humans [1]. Influenza A viruses consist of 8 RNA gene segments and are classified on the basis of the antigenicity of their surface glycoproteins: hemagglutinin (HA) and neuraminidase (NA). There are 18 different HA subtypes (H1 through H18) and 11 NA subtypes known to exist, but only three HA subtypes (H1, H2, and H3) and two NA subtypes (N1 and N2) have caused significant human epidemics [2]. The HA is the predominant viral antigen target for antibody neutralization [3].

Influenza virus causes seasonal epidemics and pandemics at irregular intervals that result in significant morbidity and mortality. According to the World Health Organization, the annual global attack rate of influenza is estimated to be 5%–10% in adults and 20%–30% in children; worldwide these annual epidemics result in about 3 to 5 million cases of severe illness and about 250,000 to 500,000 deaths [4]. The US Centers for Disease Control and Prevention estimates that a pandemic Influenza outbreak costs the United States between $71 billion and $167 billion without counting the financial impact on commerce and society [5].

Over a million human deaths worldwide were attributed to the H2N2 influenza that occurred from 1957 until 1968. H2 influenza remains a potential public health threat because H2 influenza viruses continue to circulate in avian and swine reservoirs worldwide. Many humans would likely be susceptible to infection as H2 influenza has not circulated in humans since 1968 and herd immunity has therefore diminished [6].

The emergence of new influenza strains, continuous mutations and reassortment of circulating virus diminish the effectiveness of annual influenza vaccines [7, 8]. Furthermore, in the United States, the current manufacturing process in egg-based systems can lead to lower yields and significant lag times due to virus strain identification and vaccine production, availability, and distribution [9, 10]. These limitations have raised the need for developing a universal influenza vaccine that can provide durable, cross-strain protection against different influenza viruses, with a rapid manufacturing process in which large vaccine quantities could be produced under well-controlled conditions. A universal influenza vaccine would eliminate the need for annual reformulation and revaccination, and improve pandemic preparedness [11].

With the goal of developing a universal influenza vaccine, the Vaccine Research Center (VRC), National Institute of Allergy and Infectious Diseases (NIAID), National Institutes of Health (NIH) has engineered a ferritin vaccine (VRC-FLUNPF081-00-VP) that enhances the presentation of the H2 glycoprotein to the immune system, improving neutralization potency and breadth against influenza virus subtype H2. VRC-FLUNPF081-00-VP (HA-F A/Sing) is an investigational vaccine that has not been administered to humans before this study. The HA-F A/Sing vaccine could play an important role in the planning and preparation for future influenza pandemics.

1.2 Rationale For Development Of Influenza HA-F A/Sing Vaccine, VRC-FLUNPF081-00-VP

New vaccine platforms and production technologies directed toward the goal of a universal influenza vaccine include cell-culture-based manufacturing processes, novel live attenuated vaccines, recombinant proteins, recombinant DNA-based vaccines, and nanoparticles [9, 10].

Ferritin is a ubiquitous iron storage protein, highly conserved, and found in various species from Archaea, Eukarya, and Bacteria domains [12], that can be used in vaccines for antigen presentation of influenza HA [13]. Ferritin self-assembles into a nearly-spherical nanoparticle, composed of 24 subunits organized in an octahedral symmetry with a hollow interior, that mimics the structure of the viral antigen and mediates the interaction with the immune system [13, 14]. The advantages of using ferritin as a vaccine platform to improve antigen presentation and immune stimulation against different strains of influenza viruses rely on the ability to obtain higher levels of protein quaternary structures and the capacity to display heterologous antigens on their surface [15] [13]. Furthermore, as the self-assembly process requires no energy and can be manufactured from simple expression vectors (without relying on egg-based systems), vaccine manufacturing timelines would be potentially shortened which would improve the response to an influenza pandemic [13].

Investigators at the VRC, NIAID, NIH, Bethesda, MD have identified a non-haem ferritin from *Helicobacter pylori* (*H. pylori)* as a protein able to display eight trimeric influenza HA spikes that mimics the physiological HA structure of A/Singapore/1/1957 influenza. [13]. Kanekiyo, *et al.* genetically fused the ectodomain of A/New Caledonia/20/1999 (H1N1) HA to *H. pylori* ferritin, creating a synthetic ferritin that antigenically resembles the native HA viral spikes on the surface of the ferritin spherical core [13]. In preclinical immunogenicity studies, this HA ferritin vaccine has shown to elicit two types of broadly neutralizing antibodies against the highly conserved HA stem and to the conserved receptor binding site (RBS) of the viral HA, which are structures of major interest for the development of a universal vaccine against influenza [16]. Also, this HA ferritin vaccine possesses the desired structural properties, and has been shown to have the capacity to enhance the potency and breadth of neutralizing antibodies in pre-clinical studies when it is compared to the current commercial trivalent inactivated vaccine (TIV) containing the same 1999 H1N1 HA [13].

Based on data reported with the HA ferritin vaccine in animal studies (Section 2.3), it is expected that the HA -F A/Sing vaccine will be safe and immunogenic in this Phase I study in humans. H2 was selected for evaluation of this vaccine concept both because of the low seroprevalence in large segments of the human population and the potential for future outbreaks in humans [17].

This is the first study to test the VRC-FLUNPF081-00-VP vaccine, and there is no previous human experience with this product.

1.3 Rationale For Development Of Influenza Plasmid DNA A/Sing Vaccine, VRC-FLUDNA082-00-VP and its use with VRC-FLUNPF081-00-VP in Prime-Boost Regimens

VRC-FLUDNA082-00-VP was developed by the VRC and is a plasmid DNA vaccine that encodes the H2 protein of Influenza A/Singapore/1/1957. This is the first study to test the VRC-FLUDNA082-00-VP vaccine in a clinical trial, and there is no previous human experience with this product.

In this clinical trial, we will evaluate a prime-boost regimen based on data generated by the VRC in assessing candidate H5 DNA and H7 DNA vaccines [18-23]. DNA A/Sing-prime and HA-F A/Sing-boost will be evaluated and compared to HA-F A/Sing-prime and HA-F A/Sing-boost in a 16-week interval. In previous studies using a boost interval of 12-24 weeks for a DNA vaccine-prime and Monovalent Inactivated Vaccine (MIV) boost regimen, the VRC has found that antibody responses were higher in the DNA-MIV regimen compared to the MIV-MIV vaccination regimen[24] [23]. Based on these studies, priming with a DNA A/Sing or HA-F A/Sing vaccine followed by a boost with HA-F A/Sing vaccine in a 16 week interval is being evaluated in this trial.

1.3.1 DNA vaccines developed by VRC, NIAID, NIH

DNA vaccines have been shown to be an efficient platform technology to respond to emerging infectious diseases [25]- they have provided a new alternative to induce humoral and cellular immune responses, they are cost-effective to produce and store, and they are more stable than traditional vaccines and have an excellent safety profile [26]. VRC plasmid DNA vaccines have demonstrated protective immune responses against influenza in preclinical studies [27, 28]. VRC DNA vaccines have been evaluated for safety and immunogenicity in human clinical trials, and shown to be safe, well tolerated, and capable of eliciting immune responses to targeted influenza antigens [24].

Cumulatively, since the first clinical trial using the VRC DNA vaccine platform in October 2001, more than 3,000 study subjects, 18-70 years of age, have received one or more injections of VRC DNA vaccines, which have all been evaluated as safe and immunogenic [18, 29-38].

Although a human efficacy study has not yet been completed with any DNA vaccine given alone, both VRC clinical trials of West Nile Virus (WNV) vaccines [29, 30], a severe acute respiratory syndrome (SARS) virus DNA vaccine (VRC-SRSDNA015-00-VP) [32], an H5 influenza DNA vaccine (VRC-AVIDNA036-00-VP) [18, 19], and an H7 influenza DNA vaccine (VRC-FLUDNA071-00-VP) [23], provided evidence that DNA vaccines can induce neutralizing antibodies (NAbs) against targeted pathogens.

1.4 Rationale for VRC-FLUDNA082-00-VP Vaccine Administration Method

VRC DNA vaccines have been administered at dosages of up to 8 mg, however the majority of injections have been given at 4 mg. Data from two dose-escalation studies (VRC 004 and VRC 204) indicate that a 4 mg dosage offers the combination of a good safety profile, greater ease of administration than an 8 mg dosage, and reliable immunogenicity as indicated by laboratory measures of immune response. Preclinical and clinical evaluations to date of plasmid DNA vaccines support the safety and immunogenicity of DNA vaccines at 4 mg.

In previous studies, DNA vaccines have been administered by intramuscular (IM), intradermal or subcutaneous routes using either standard needle and syringe or Biojector (Biojector 2000® needle-free injection system, Bioject Medical Technologies, Tigard, OR). It was demonstrated that the use of a needle-free injection system is superior to needle and syringe by offering a significant contribution to the elicited immunogenicity [39].

In March 2016, Biojector became unavailable. Consequently, another needle-free injection system from PharmaJet Stratis® (Golden, CO) is available. The PharmaJet Stratis® 0.5 mL Needle-Free Jet Injector (PharmaJet) is FDA approved for use in children and adults. PharmaJet holds a General Use 510k marketing clearance from FDA and received the Performance, Quality

and Safety (PQS) pre-qualified certification from the WHO (#E008/050). PharmaJet provides an opportunity to deliver the vaccine by a needle-free device, to simplify clinical operations and potentially increase elicited immunogenicity. Immunogenicity studies have been conducted in rhesus macaques in which animals received doses of the Zika virus (ZIKVwt) DNA vaccine via PharmaJet showed an increase in NAb production following immunization in both groups [40].

In this Phase I study in healthy adults, study subjects enrolled only in Group 3 will receive 1 IM vaccination with the VRC-FLUDNA082-00-VP vaccine via PharmaJet on Day 0 as per the Schedule of Evaluations (Appendix II).

1.5 Rationale For Study Population Proposed In The Clinical Trial

To assess safety, tolerability, and immunogenicity of the novel HA-F A/Sing vaccine platform, two age cohorts will be evaluated in part II of this study. These age ranges are based on the likelihood of prior H2 exposure; human H2 influenza last circulated as a seasonal strain between 1957 and 1968. Therefore, the younger age cohort, age 18 through adults born after 1969, should have no pre-existing immunity to H2. The older age cohort, adults born in or before 1965 through age 70, may have some immunity. The age gap in which subjects are ineligible, adults born between January 1, 1966 and December 31, 1969, excludes subjects with unknown exposure. H2 immunity will not be evaluated as an eligibility criterion.

1.6 Assessment of Vaccine Immunogenicity

In this study, specimens to evaluate immunogenicity will be collected at baseline and at specified time points. The primary immunogenicity time point is two weeks after the boost vaccination. HA-specific antibody as measured by HAI assay is the traditional benchmark measure of immune response to influenza vaccines and will be conducted on stored samples obtained throughout the study. Additional measurements of human ferritin antibody, *H. pylori* ferritin antibody, and T cell responses may also be assessed from stored samples for time points throughout the study as exploratory evaluations.

The detection of antibody by HAI or neutralization assay is based on validated laboratory methods. Exploratory T cell assays may include the intracellular cytokine staining (ICS) assay which is based on previously published methods [41] and quantitates of the frequency of $CD4^+$ and $CD8^+$ cells that produce interleukin-2, interferon-gamma or tumor necrosis factor (TNF)-alpha in response to pools of overlapping peptides representing HA antigens.

Research samples for immunogenicity assays will be processed by the Vaccine Immunology Testing Laboratory (VITL) in Gaithersburg, MD, where many of the immunogenicity assays will also be performed. Some immunogenicity assays may be performed by VRC laboratories in Bethesda, MD, approved contract laboratories, or approved research collaborators.

Results from this study are expected to provide a foundation for building a universal influenza vaccine candidate as well as show proof-of-concept for engineered HA-ferritin as a vaccine platform.

2. STUDY AGENTS

The two study agents (VRC-FLUNPF081-00-VP and VRC-FLUDNA082-00-VP) are manufactured under current Good Manufacturing Practice (cGMP) by the Vaccine Clinical Materials Program (VCMP) operated under contract by Leidos Biomedical Research, Inc., Frederick, MD.

2.1 VRC-FLUNPF081-00-VP

VRC-FLUNPF081-00-VP (HA-F A/Sing) is a vaccine composed of HA protein from Influenza A/Singapore/1/1957 genetically fused to ferritin protein from *H. pylori*.

The purified HA-F A/Sing displays eight well-formed HA trimers that antigenically resembles the native H2 viral spikes.

VRC-FLUNPF081-00-VP vaccine is a sterile, aqueous buffered solution. Vials are aseptically filled to a volume of 0.7 mL into a single dose vials at 180 mcg/mL. The buffer consists of 20mM NaPhosphate, 100mM NaCl, and 5% Sucrose, at pH 7.2.

More details related to vaccine production, preparation, quality control and preclinical studies performed with HA-F A/Sing vaccine can be found in the Investigator's Brochure (IB).

2.2 VRC-FLUDNA082-00-VP

VRC-FLUDNA082-00-VP (DNA A/Sing) is a DNA vaccine that encodes the hemagglutinin (H2) protein of Influenza A/Singapore/1/1957.

VRC-FLUDNA082-00-VP is a single closed circular plasmid DNA (plasmid VRC 9265) that contains a human cytomegalovirus regulatory R region (CMV/R) as a promoter. The CMV/R promoter consists of a translational enhancer region of the CMV immediate-early region one enhancer (CMV-IE) substituted with the 5 '-untranslated human T-cell leukemia virus type 1 (HTLV-1) R-U5 region (HTLV-1 R-U5) long terminal repeat (LTR). This promoter has been shown to optimize gene expression [42] and it has been evaluated in preclinical safety studies [43], as well as several clinical trials of DNA plasmid vaccines for Human Immunodeficiency Virus (HIV) (BB-IND 11750), WNV (BB-IND 12933), Ebola virus (BB-IND 11294), SARS (BB-IND 11995), avian influenza virus (BB-IND 13197), and Zika virus (BB-IND 17080).

VRC-FLUDNA082-00-VP vaccine is formulated in phosphate buffered saline (PBS) solution. Vials are aseptically filled to a volume of 1.2 mL with 4 mg/mL of the plasmid.

More details related to vaccine production, preparation and quality control of VRC-FLUDNA082-00-VP can be found in the IB.

2.3 VRC-PBSPLA043-00-VP, PHOSPHATE BUFFERED SALINE (DILUENT)

Diluent consists of sterile phosphate buffered saline (PBS) at pH 7.2, aseptically filled at 1.2 mL in a 3 mL glass vial for single use. Diluent vials will be stored until use at -45°C to -10°C.

2.4 PRECLINICAL STUDIES WITH VRC-FLUNPF081-00-VP

Non-clinical immunogenicity studies in mice confirmed that the VRC-FLUNPF081-00-VP Drug Product administered IM is immunogenic as detected by hemagglutinin inhibition (HAI) and neutralization assay.

A repeat dose toxicity study was performed in New Zealand white rabbits in accordance with the Good Laboratory Practices (GLP) Regulations for nonclinical laboratory studies. IM administration of either the monovalent influenza vaccine (60 µg of VRC-FLUNPF081-00-VP) or the trivalent influenza vaccine (60 µg VRC-FLUNPF081-00-VP; 60 µg VRC-FLUNPF088-00-VP; 60 µg VRC-FLUNPF089-00-VP) once every three weeks for three injections (Study Days 1, 22, and 43) was well tolerated as there were no adverse or limiting toxicity seen throughout the study and any findings noted were considered to be of minimal toxicological significance (e.g., noted in only one sex, reversible, transient, no alteration in organ function, etc.).

More details on pre-clinical studies with VRC-FLUNPF081-00-VP can be found in the IB.

2.5 Preclinical Studies with VRC-FLUDNA082-00-VP

No preclinical pharmacology, toxicology, pharmacokinetic, or metabolism studies were conducted for VRC-FLUDNA082-00-VP. VRC DNA products have been extensively tested in animals and humans, alone and as a prime for various vaccine boosts, and have shown a robust safety profile. Early biodistribution studies showed complete DNA clearance by three months [43].

More details on pre-clinical studies with DNA vaccines can be found in the IB.

3. STUDY OBJECTIVES

3.1 Primary Objectives

- To evaluate the safety and tolerability of the HA-F A/Sing vaccine, administered as a single dose at 20 mcg IM via needle and syringe at Day 0 to healthy adults.

- To evaluate the safety and tolerability of the HA-F A/Sing vaccine, administered at 60 mcg IM via needle and syringe to healthy adults by repeat dosing at Day 0 and Week 16 for a total of 2 injections.

- To evaluate the safety and tolerability of the DNA A/Sing vaccine, administered as a single dose at 4 mg IM via needle-free injection device at Day 0 to healthy adults in the prime-boost regimen.

3.2 Secondary Objectives

- To evaluate the antibody response to two prime-boost vaccine regimens in healthy adults:

  1) DNA A/Sing vaccine administered at 4 mg IM via needle-free injection device at Day 0 followed by HA-F A/Sing vaccine administered at 60 mcg IM via needle and syringe at Week 16.
  2) HA-F A/Sing vaccine administered at 60 mcg IM via needle and syringe by repeat dosing at Day 0 and Week 16.

- To compare the magnitude and the frequency of H2-specific antibody response as measured by HAI at four weeks after the prime vaccine and two weeks after the boost vaccine for each treatment and age group.

3.3 Exploratory Objectives

- To evaluate the magnitude, specificity and frequency of the H2-specific T-cell, B-cell responses and antibody responses at indicated time points throughout the study.

4. STUDY DESIGN AND METHODS

This is a Phase I, open-label, dose escalation study to evaluate the dose, safety, tolerability, and immunogenicity of the VRC-FLUNPF081-00-VP vaccine alone or in prime-boost regimens with the VRC-FLUDNA082-00-VP vaccine in healthy adults. The study schema is shown in **Table 2**. The hypotheses are that the vaccines are safe, well-tolerated, and will induce an antibody response to influenza virus subtype H2. The study will be conducted by the VRC Clinical Trials Program at a single site at the NIH Clinical Center (NIH CC).

**Table 2: VRC 316 Vaccination Schema**					
**PART I: Dose Escalation of HA-F A/Sing**					
**Group**	**Dose Level**	**Age Cohorts**	**Subjects**	**Day 0 Prime**	**Week 16 (±4 weeks) Boost**
1	Low dose	Ages 18-47	**5**	HA-F A/Sing, 20 mcg	
2	High dose	Ages 18-47	**5**	HA-F A/Sing, 60 mcg	HA-F A/Sing, 60 mcg
**Total**			**10***	*Enrollment of up to 15 subjects is permitted if additional subjects are needed for safety or immunogenicity evaluations.	
				HA-F A/Sing injections are administered by needle and syringe.	
**PART II: Evaluation of HA-F A/Sing and DNA A/Sing in Prime-Boost Regimens**					
**Group**	**Regimen**	**Age Cohorts**	**Subjects**	**Day 0 Prime**	**Week 16 (±4 weeks) Boost**
3	DNA A/Sing<br>HA-F A/Sing	Group 3A; 18-47	**10**	DNA A/Sing, 4 mg	HA-F A/Sing, 60 mcg
		Group 3B; 52-70	**10**		
4	HA-F A/Sing<br>HA-F A/Sing	Group 4A; 18-47	**10**	HA-F A/Sing, 60 mcg	HA-F A/Sing, 60 mcg
		Group 4B; 52-70	**10**		
**Total**			**40***	*Enrollment of up to 65 subjects is permitted if additional subjects are needed for safety or immunogenicity evaluations.	
				DNA A/Sing injections are administered by PharmaJet. HA-F A/Sing injections are administered by needle and syringe.	

4.1 Study Population

All inclusion and exclusion criteria must be evaluated for eligibility.

4.1.1 Inclusion Criteria

A subject must meet all of the following criteria:

1. Healthy subjects aged 18-70 (excluding subjects born between 1966-1969)
2. Based on history and examination, must be in good general health and without history of any of the conditions listed in the exclusion criteria
3. Received at least one licensed influenza vaccine from 2014 to the present
4. Able and willing to complete the informed consent process
5. Available for clinic visits for 40 weeks after enrollment
6. Willing to have blood samples collected, stored indefinitely, and used for research purposes
7. Able to provide proof of identity to the satisfaction of the study clinician completing the enrollment process
8. Physical examination and laboratory results without clinically significant findings and a Body Mass Index (BMI) ≤ 40 within the 84 days before enrollment

Laboratory Criteria within 84 days before enrollment

9. White blood cells (WBC) and differential either within institutional normal range or accompanied by the site Principal Investigator (PI) or designee approval
10. Total lymphocyte count ≥ 800 cells/mm^3
11. Platelets = 125,000 – 500,000/mm^3
12. Hemoglobin within institutional normal range
13. Serum iron either within institutional normal range or accompanied by the site PI or designee approval
14. Alanine aminotransferase (ALT) ≤ 1.25 x institutional upper limit of normal (ULN)
15. Aspartate aminotransferase (AST) ≤ 1.25 x institutional ULN
16. Alkaline phosphatase (ALP) ≤ 1.1 x institutional ULN
17. Total bilirubin within institutional upper limit of normal (ULN)
18. Serum creatinine ≤ 1.1 x institutional ULN
19. Negative for HIV infection by an FDA approved method of detection

Criteria applicable to women of childbearing potential:

20. Negative beta-human chorionic gonadotropin (β-HCG) pregnancy test (urine or serum) on the day of enrollment
21. Agrees to use an effective means of birth control from at least 21 days prior to enrollment through the end of the study

4.1.2 Exclusion Criteria

A subject will be excluded if one or more of the following conditions apply:

1. Breast-feeding or planning to become pregnant during the study.

Subject has received any of the following substances:

2. More than 10 days of systemic immunosuppressive medications or cytotoxic medications within the 4 weeks prior to enrollment or any within the 14 days prior to enrollment
3. Blood products within 16 weeks prior to enrollment
4. Live attenuated vaccines within 4 weeks prior to enrollment
5. Inactivated vaccines within 2 weeks prior to enrollment.
6. Investigational research agents within 4 weeks prior to enrollment or planning to receive investigational products while on the study
7. Current allergy treatment with allergen immunotherapy with antigen injections, unless on maintenance schedule
8. Current anti-TB prophylaxis or therapy
9. Previous H2 influenza investigational vaccine
10. Receipt of a licensed influenza vaccine within 6 weeks before trial enrollment

Subject has a history of any of the following clinically significant conditions:

11. Serious reactions to vaccines that preclude receipt of study vaccinations as determined by the investigator
12. Hereditary angioedema, acquired angioedema, or idiopathic forms of angioedema
13. Asthma that is not well controlled
14. Diabetes mellitus (type I or II), with the exception of gestational diabetes
15. Thyroid disease that is not well controlled
16. Idiopathic urticaria within the past year
17. Evidence of autoimmune disease or immunodeficiency
18. Hypertension that is not well controlled
19. Bleeding disorder diagnosed by a doctor (e.g. factor deficiency, coagulopathy, or platelet disorder requiring special precautions) or significant bruising or bleeding difficulties with IM injections or blood draws
20. Malignancy that is active or history of malignancy that is likely to recur during the period of the study.
21. Seizure disorder other than 1) febrile seizures, 2) seizures secondary to alcohol withdrawal more than 3 years ago, or 3) seizures that have not required treatment within the last 3 years
22. Asplenia, functional asplenia or any condition resulting in the absence or removal of the spleen
23. Guillain-Barré Syndrome
24. Psychiatric condition that precludes compliance with the protocol; past or present psychoses; or within 5 years prior to enrollment, a history of suicide plan or attempt

25. Any medical, psychiatric, social condition, occupational reason or other responsibility that, in the judgment of the investigator, is a contraindication to protocol participation or impairs a subject's ability to give informed consent.

4.2 Clinical Procedures and Evaluations

Evaluation of this vaccine will include laboratory tests, medical history, physical assessment by clinicians, and subject self-assessment recorded on a diary card for 7 days after each injection. Potential adverse reactions will be further evaluated prior to continuing the vaccination schedule. The schedule of study evaluations is described in this section and shown in table format in Appendix II. Total blood volume drawn from each subject will not exceed the NIH CC Guidelines.

4.2.1 Screening

Screening for this study will be completed through the VRC's screening protocol, VRC 500 (NIH 11-I-0164). Subjects will be recruited through Institutional Review Board (IRB)-approved advertising. The evaluations and sample collection that will be included in screening are a medical history, physical exam, any laboratory tests needed to confirm eligibility, and pregnancy test for females of reproductive potential.

If screening evaluations suggest a current concerning health condition or infection, such as hepatitis, then appropriate laboratory tests may be conducted to evaluate for these conditions. Additional assessments of health may be conducted at screening based on clinical judgment. Screening evaluations for specific eligibility criteria (Section 4.1) must be completed within the time interval specified prior to enrollment for the given parameter and may be repeated, as needed, to confirm eligibility. Research blood samples will be collected during screening; although generally collected in the 84 days prior to enrollment; a particular interval of time prior to enrollment for collection of these samples is not specified.

The informed consent form (ICF) will be reviewed and counseling related to pregnancy prevention will be provided.

Subjects who are not up to date on standard vaccinations may receive these, if available, during their participation in the screening protocol or at a later date during study participation. As part of the informed consent process, an Assessment of Understanding (AoU) is completed on the day the subject is scheduled to enroll prior to signing the VRC 316 protocol informed consent document. Incorrect answers will be explained to the volunteer, who will sign the ICF only after the study clinician is satisfied with the volunteer's understanding of the study.

4.2.2 Study Schedule

The Schedule of Evaluations in Appendix II provides details on the study schedule and the permitted visit windows. A clinician will discuss the target dates and timing of the study vaccination(s) and sample collections before completing an enrollment to help ensure that the subject can comply with the projected schedule.

The schedule of study visits, permitted windows for completing the visits, and evaluations performed at each visit are shown in Appendix II. After enrollment, deviations from the visit windows in completing study visits and study injections are discouraged and will be recorded as protocol deviations but are permitted at the discretion of the PI (or designee) in the interest of completing the vaccination schedule and obtaining subject safety and immunogenicity evaluations.

4.2.3 Enrollment and Randomization

Day 0 is defined as the day of protocol enrollment and first injection for all groups. Protocol-specific eligibility is reviewed on Day 0 as part of the enrollment process, but eligibility evaluations conducted during a screening visit are routinely used for eligibility if evaluations are within the specified window prior to Day 0. However, if clinical assessment on Day 0 suggests significant changes may have occurred since the screening visit, then evaluations done on Day 0 are used for eligibility. Day 0 evaluations and medical history prior to the first injection are the baseline for subsequent safety assessments.

Part I includes staged enrollment with required interim safety reviews before proceeding with the next dose group. In Group 1, five subjects will be enrolled with no more than one subject per day. Following dose escalation review, five subjects will be enrolled in Group 2 with no more than one subject per day. An internal safety review will determine whether enrollment can begin in Part II.

In Part II subjects will be stratified by age and randomized equally into Group 3 and Group 4. Neither the clinic staff nor the subjects will know in advance which group will be assigned to the enrollees in the study. The Protocol Statistician will prepare the randomization plan in advance and provide it to the site Pharmacy and Data Management Center.

The pharmacy database and study randomization will be set up prior to opening the study to accrual. The group assignment will become known to the staff and subject immediately after completing the electronic enrollment into the study on Day 0. The visit schedule is based on intervals of time after each study injection (Appendix II).

4.2.4 Administration of Study Vaccines

All study injections will be completed according to the assigned group and vaccines will be administered IM in the deltoid muscle. Blood draws must be completed before vaccinations.

On the injection day (prior to injection), study subjects will be clinically evaluated and samples will be collected as per Schedule of Evaluations (Appendix II). A subject who arrives at the clinic with fever or evidence of an acute illness that precludes administration of the vaccine may be rescheduled within the allowed study visit window.

Pregnancy test results for women of reproductive potential must be obtained on each injection day prior to the study injection and the results must be negative to proceed.

When choosing an arm for injection, clinicians should consider whether there is an arm injury, local skin problem or significant tattoo that precludes administering the injection or will interfere with evaluating the arm after injection.

Subjects enrolled in Group 1, Group 2, and Group 3 will be observed for a minimum of 1 hour. Vital signs (temperature, blood pressure, pulse and respiratory rate) will be collected at least 1 hour after the injection, prior to subject departure from the clinic. Subjects enrolled in Group 4 will be observed for a minimum of 30 minutes; vital signs will be collected after at least 30 minutes. The injection site will be inspected for evidence of local reaction.

Subjects randomized in Group 3 will receive the DNA A/Sing prime vaccine with the needle-free injection device, PharmaJet. The PharmaJet device requires that the DNA A/Sing product be administered as two 0.5 mL injections. When the PharmaJet device is used, subjects and clinicians will be asked to wear safety glasses during product administration. (See Section 8.5.2)

In keeping with the NIH CC policy and good medical practice, acute medical care will be provided to subjects for any immediate allergic reactions or other injury resulting from participation in this research study.

4.2.5 7-Day Solicited Reactogenicity and Follow-up

Subjects will be given a "Diary Card" to use as a memory aid, on which to record temperature and symptoms daily for 7 days after each injection. Subjects will be trained and encouraged to use the secure electronic database but will have the option to complete a paper diary card. When the diary card parameters are recorded directly by the subject in the electronic database, the subject's electronic record will be the source for these data. When collected on paper, the paper diary card will be used as a source document. When neither paper nor electronic diary is available from the subject, the study clinician will document the source of reactogenicity information recorded in the study database.

The solicited signs and symptoms on the diary card will include the following parameters: unusually tired/feeling unwell, muscles aches (other than at injection site), headache, chills, nausea, joint pain, and pain/tenderness at injection site. Subjects will also record the day's highest measured temperature and measurement of largest diameter for redness and swelling at injection site.

Follow-up on subject well-being will be performed by telephone on the first day following all injections and by clinic visits as shown in the Schedule of Evaluations (Appendix II)

Events following any study injection that may require clinical evaluation include rash, urticaria, fever of 38.5˚C (Grade 2) or higher lasting greater than 24 hours, or significant impairment in the activities of daily living. Additionally, other clinical concerns may prompt a study visit based on the judgment of a study clinician.

4.2.6 Follow-Up through End of Study

Study follow-up will continue via clinic visits through 40 weeks following the first vaccine administration. Refer to Section 4.4 which describes the criteria for discontinuing product administration and/or study participation.

4.2.7 Blood Sample Collection

At intervals throughout the study, blood will be drawn for safety and immunologic assays. Blood will be drawn from the arm veins of subjects by standard phlebotomy procedures. Total blood volume drawn from each subject will not exceed the NIH CC Guidelines.

4.2.8 Apheresis

Apheresis will be offered as an optional procedure at Visit 08 (except for Group 1). Each apheresis procedure will be carried out by trained Department of Transfusion Medicine (DTM) medical staff using automated cell separator devices.

All apheresis procedures performed under this protocol will be performed solely for research purposes. Prior to the scheduling apheresis, the subject must have a venous assessment performed by the DTM staff.

In order to undergo apheresis procedures, a subject must meet the apheresis eligibility criteria and have no medical contraindications, as determined by the DTM staff. A VRC study clinician will complete a checklist for apheresis eligibility before referring a subject for the procedure.

4.2.8.1 Apheresis Procedures

All study subjects will be treated according to standard whole blood and apheresis donation policies and procedures operative in the DTM.

For women of reproductive potential, a pregnancy test by blood or urine will be performed by a VRC study clinician within 72 hours prior to the apheresis procedure. Results must be negative to proceed with apheresis.

Prior to beginning the apheresis procedure, a study clinician may request in advance that other laboratory tests be collected as needed to monitor the well-being of the subject or samples as needed by a research laboratory.

The Dowling Apheresis Clinic staff at the NIH Clinical Center routinely performs a hemoglobin test prior to initiating apheresis, per DTM Apheresis Clinic standard policies. If a subject is found to have a hemoglobin value less than permitted by the Apheresis Clinic, then the apheresis will not be initiated and the ordering provider will be notified. The VRC Study Coordinator will provide the Apheresis center with a request for numbers and types of tubes of blood to be collected prior to beginning the apheresis.

In this study, the procedure will require two antecubital venous access sites and will involve processing 1 to 4 liters of whole blood. The expected mononuclear cell yield is approximately 0.5 to 1.0 x 10^9 cells per liter processed, and the apheresis device can process about 2-3 liters per hour. Thus, 1 to 2 hours are required to process 1 to 4 liters of blood and obtain about 1-4 x 10^9 leukocytes. The packed red cell loss during the procedure is the equivalent of a 6 mL blood draw; this is the volume that will be used for the purposes of calculating cumulative blood draw when apheresis is performed.

During or following an apheresis visit, if there is any concern about the well-being of the subject, the DTM clinic may conduct appropriate medical evaluations by history-taking, physical examination, laboratory tests, and/or other testing.

Research blood samples will be processed and stored at VITL or a collaborating research laboratory. Stored samples may be used later to further evaluate immune responses and to elucidate genetic factors associated with immune response.

4.2.9 Concomitant Medications

Only routine prescription medications will be entered in the database at the time of enrollment. Subsequently, concomitant medications associated with an AE requiring expedited reporting or the development of a new chronic condition requiring ongoing medical management will be recorded or updated in the database. Antiviral medications taken for influenza or influenza-like illnesses will be recorded in the database. Receipt of a FDA-approved vaccine at any time during the study will be recorded in the database (clinicians should work with subjects regarding the timing of licensed vaccines relative to study injection). Otherwise, concomitant medications taken throughout the study will be recorded in the subject's chart as needed for general medical documentation but will not be recorded in the database.

4.3 Criteria for Dose Escalation and Dose Continuation

There will be one dose escalation in this study. The Protocol Safety Review Team (PSRT, Section 8.8) will conduct an interim safety data review before dose escalation and repeat dosing

may occur. The PSRT must assess the data as showing no significant safety concerns before enrollment of the next dose level and repeat dosing at the same level may proceed.

Enrollment will begin in Group 1 (20 mcg of VRC-FLUNPF081-00-VP) with no more than one subject enrolled per day. Two weeks after vaccination of the fifth volunteer, there will be an internal safety review on all five subjects to determine whether to proceed to the next dose level. If the 20 mcg dose of VRC-FLUNPF081-00-VP vaccine is assessed as safe, enrollment will begin for Group 2.

In Group 2 (60 mcg of VRC-FLUNPF081-00-VP), enrollment continues with no more than one subject enrolled per day. Two weeks after vaccination of the fifth volunteer, there will be an internal safety review on all five subjects to determine whether to proceed to Part II. If the 60 mcg dose of VRC-FLUNPF081-00-VP vaccine is assessed as safe, subjects in Group 2 will proceed to receive a second vaccination at week 16 and enrollment can begin for all groups in Part II.

If a first vaccination is not completed or there are discontinuations from the study before there are sufficient data to conduct the dose escalation review for a group, then extra subjects may be enrolled into that group in order to have the requisite data on at least 5 subjects.

The IRB will be provided with documentation of the safety review process and notification of the dose escalation. Consultation with the IRB and FDA, if needed, as per study pause criteria (Section 4.5) will occur if indicated by the review. One outcome of a dose escalation review may be to recommend evaluation of additional subjects at the current dose level and reassess for safety before proceeding to a higher dose level and repeat dosing at the same dose level.

4.4 Criteria for Discontinuing Study Injections or Protocol Participation

Decisions on discontinuation of study injections or protocol participation for a subject will be made by the site PI or designee.

4.4.1 Discontinuation of Study Injections

Subjects who receive at least one injection will continue follow-up according to the Schedule of Evaluations., except that the research sample collections will be discontinued for pregnant women or others in which it is contraindicated A subject may be discontinued from receiving study product for the following reasons:

1. Pregnancy.
2. Grade 3 AE assessed as related to study product (with the exception that self-limited Grade 3 solicited reactogenicity does not require discontinuation).
3. Grade 4 AE assessed as related to study product.
4. Immediate hypersensitivity reaction associated with study product.
5. Intercurrent illness that is not expected to resolve prior to the next scheduled study injection.
6. Treatment with systemic glucocorticoids (e.g., prednisone or other glucocorticoid) or other immunomodulators (other than nonsteroidal anti-inflammatory drugs [NSAIDs]), with the exception that study injections may continue per investigator discretion if the next dose occurs at least 2 weeks following completion of glucocorticoid treatment.

7. The study PI assesses that it is not in the best interest of the subject to continue on the vaccination schedule.

4.4.2 Discontinuation of Protocol Participation

A subject may be discontinued from protocol participation for the following reasons:

1. Subject decides to discontinue participation;
2. Subject develops a medical condition that is a contraindication to continuing study participation;
3. The IND sponsor or regulatory authority stops the protocol;
4. The PI assesses that is it not in the best interest of the subject to continue participation in the study or that the subject's compliance with the study is not sufficient.

4.5 Criteria for Pausing and Resuming the Study

4.5.1 Plan for Pausing the Study

The PI and Protocol Safety Review Team (PSRT) will closely monitor and analyze study data as they become available and will make determinations regarding the presence and severity of AEs. The administration of study injections and new enrollments will be paused and the IND Sponsor will be promptly notified according to the following criteria:

- **One** (or more) subject experiences a **SAE** assessed as related to study product.
- **Two** (or more) subjects experience the same **Grade 3 or higher unsolicited AE** assessed as related to study product.
- T**hree** (or more) subjects experience the same **Grade 2 or higher laboratory abnormality** assessed as related to study product.

4.5.2 Plan for Review of Pauses and Resuming the Study

The IND Sponsor, with participation by the PI and PSRT, will conduct the review and make the decision to resume, amend or close the study. As part of the pause review, the reviewers will also advise on whether the study needs to be paused again for any subsequent AEs of the same type. The pause criterion for the SAE will continue to apply.

The administration of study injections and new enrollments would resume only if review of the AEs that caused the pause resulted in a recommendation to permit further study injections and enrollments. Safety data reports and changes in study status will be submitted to relevant regulatory authorities in accordance with Section 5 and institutional policy.

5. SAFETY AND ADVERSE EVENT REPORTING

5.1 Adverse Events

An adverse event (AE) is any untoward or unfavorable medical occurrence in a human subject, including any abnormal sign (e.g., abnormal physical exam or laboratory finding), symptom, or disease temporally associated with the use of study treatment, whether or not considered related to the study treatment.

Each AE will be graded according to the Table for Grading Severity of Adverse Events (Appendix III). The following guidelines will be used to determine whether or not an AE is recorded in the study database:

- Solicited AEs (i.e., reactogenicity parameters) will be recorded by the subject in paper or electronic diary cards for 7 days after each injection. If paper diary card is completed by subject, data will be transcribed by clinician into the study database without attribution assessments. Clinicians will follow and collect resolution information for any reactogenicity symptoms that are not resolved within 7 days.
- All unsolicited AEs will be recorded in the study database from receipt of first study injection through the "Week 4" visit after each study injection with attribution assessments. At other time periods between injections and when greater than 4 weeks after the booster injection, only SAEs (Section 5.2), new chronic medical conditions, and influenza or influenza-like illness (ILI) will be recorded through the last study visit.
- Cases of influenza or influenza-like illness will be evaluated using clinical judgment and current CDC definition of influenza symptoms (https://www.cdc.gov/flu/about/disease/complications.htm) and recorded on a case report form rather than on an AE form. ILI is defined as fever (temperature of 100F [37.8C] or greater) and a cough and/or sore throat in the absence of a known cause other than influenza.

5.2 Serious Adverse Events

The term "Serious Adverse Event" (SAE) is defined in 21 CFR 312.32 as follows: "An adverse event or suspected adverse reaction is considered serious if, in the view of either the investigator or the sponsor, it results in any of the following outcomes: Death, a life-threatening adverse event, inpatient hospitalization or prolongation of existing hospitalization, a persistent or significant incapacity or substantial disruption of the ability to conduct normal life functions, or a congenital anomaly/birth defect. Important medical events that may not result in death, be life-threatening, or require hospitalization may be considered serious when, based upon appropriate medical judgment, they may jeopardize the patient or subject and may require medical or surgical intervention to prevent one of the outcomes listed in this definition. Examples of such medical events include allergic bronchospasm requiring intensive treatment in an emergency room or at home, blood dyscrasias or convulsions that do not result in inpatient hospitalization, or the development of drug dependency or drug abuse."

"Life-threatening" refers to an AE that at occurrence represents an immediate risk of death to the subject. An event that may cause death if it occurs in a more severe form is not considered life-threatening. Similarly, a hospital admission for an elective procedure is not considered a SAE.

5.3 Adverse Event Reporting to the IND Sponsor

AEs that meet SAE Reporting Requirements must be reported and submitted by the clinical site on an expedited basis to the IND Sponsor, VRC/NIAID/NIH, according to sponsor guidelines as follows:

- results in death
- is life threatening

- results in persistent or significant disability/incapacity
- requires unplanned inpatient hospitalization or prolongation of existing hospitalization
- is a congenital anomaly/birth defect in the offspring of a study subject
- is an important medical event that may jeopardize the subject or may require intervention to prevent one of the other outcomes listed above.

In addition, any event, regardless of severity, which in the judgment of the PI represents a SAE, may be reported on an expedited basis.

An investigator will communicate an initial SAE report within 24 hours of site awareness of occurrence to the IND Sponsor by email to the VRC Protocol Operations Office (Appendix I).

A written report by PI should be submitted to the IND Sponsor within 3 working days. In order for the IND Sponsor to comply with regulations mandating sponsor notification of specified SAEs to the FDA within 7 or 15 calendar days, the investigator must submit additional information as soon as it is available.

5.3.1 IND Sponsor Reporting to the FDA

It is the responsibility of the IND Sponsor to make the determination of which SAEs are "serious and unexpected suspected adverse reactions" (SUSARs) as defined in 21 CFR 312.32.

- *Suspected adverse reaction* means any AE for which there is a reasonable possibility that the drug caused the AE.
- *Unexpected Adverse Event* means an AE that is not listed in the Investigator's Brochure or is not listed at the specificity or severity that has been observed.

All SUSARs, as determined by the IND Sponsor, will be reported to the FDA as IND Safety Reports and IND Safety Reports will be provided to the IRB.

The IND Sponsor will also submit an IND Annual Report of the progress of the investigation to the FDA as defined in 21 CFR 312.33.

5.4 Reporting to the Institutional Review Board

5.4.1 Unanticipated Problem Definition

An unanticipated problem (UP) is defined as any incident, experience, or outcome that meets all three of the following criteria:

- unexpected in nature, severity, or frequency in relation to the research risks that are described in the protocol, informed consent, Investigator's Brochure, other study documents or in consideration of the characteristics of the subject population being studied; **and**
- related to participation in the research; **and**
- suggests that the research places subjects or others at a greater risk of harm (including physical, psychological, economic, or social harm) than was previously known or recognized.

Serious UP: An UP that meets the definition of a SAE or compromises the safety, welfare or rights of subjects or others.

An UP that is not an Adverse Event (UPnonAE) is an unanticipated problem that does not fit the definition of an AE, but which may, in the opinion of the investigator, involve risk to the subject, affect others in the research study, or significantly impact the integrity of research data. Such events would be considered a non-serious UP. For example, we will report occurrences of breaches of confidentiality, accidental destruction of study records or samples, or unaccounted-for study drug.

5.4.2 Protocol Deviation Definition

A protocol deviation is defined as any change, divergence, or departure from the IRB-approved study procedures in a research protocol. Protocol deviations are designated as serious or non-serious and further characterized as follows:

- Those that occur because a member of the research team deviates from the protocol.
- Those that are identified before they occur, but cannot be prevented.
- Those that are discovered after they occur.

Serious Protocol Deviation: A deviation that meets the definition of a SAE or compromises the safety, integrity of the data, welfare or rights of subjects or others.

5.4.3 Non-Compliance Definition

Non-compliance is the failure to comply with applicable NIH Human Research Protections Program (HRPP) policies, IRB requirements, or regulatory requirements for the protection of human subjects. Non-compliance is further characterized as serious, continuing or minor.

"Serious non-compliance" is defined as non-compliance that:

- Increases risks, or causes harm, to participants
- Decreases potential benefits to participants
- Compromises the integrity of the NIH-HRPP
- Invalidates the study data

"Continuing non-compliance" is non-compliance that is recurring.

"Minor non-compliance" is non-compliance that is neither serious nor continuing.

5.4.4 Expedited Reporting to the NIAID IRB

The following will be reported within 7 calendar days of investigator awareness:

- Serious and non-serious UP
- Deaths
- Serious protocol deviations
- Serious or continuing non-compliance
- SAEs that are possibly, probably, or definitely related to the research regardless of expectedness

The following waiver applies to reporting anticipated protocol deviations and expected UPnonAEs: Anticipated deviations in the conduct of the protocol will not be reported to the IRB unless they occur at a rate greater than anticipated by the study team. Expected AEs will not be

reported to the IRB unless they occur at a rate greater than that known to occur in healthy adults. If the rate of these events exceeds the rate expected by the study team, the events will be classified and reported as though they are unanticipated problems.

5.4.5 Annual Reporting to the NIAID IRB

The following will be reported to the NIAID IRB in summary at the time of Continuing Review:

- Serious and non-serious UPs
- Expected SAEs that are possibly, probably, or definitely related to the research
- SAEs that are not related to the research
- All AEs, except expected AEs granted a waiver of reporting
- Serious and non-serious Protocol Deviations
- Serious, continuing, and minor non-compliance
- Any trends or events which in the opinion of the investigator should be reported

5.5 Serious Adverse Event Reporting to the Institutional Biosafety Committee (IBC)

The NIH Institutional Biosafety Committee (IBC) (Bethesda, MD) has the responsibility to review research using recombinant DNA for compliance with NIH Guidelines. In keeping with IBC requirements, any SAE reports sent to the IRB will be subsequently provided to the IBC by the investigators.

6. STATISTICAL CONSIDERATIONS

6.1 Overview

This is a Phase I, open-label, dose escalation study to evaluate the dose, safety, tolerability, and immunogenicity of VRC-FLUNPF081-00-VP vaccine alone or in prime-boost regimens with VRC-FLUDNA082-00-VP vaccine.

6.2 Objectives

The primary objectives are to evaluate the safety and tolerability of VRC-FLUNPF081-00-VP vaccine when administered as a single dose at 20 mcg, and to evaluate the safety and tolerability of two prime-boost vaccine regimens:

- VRC-FLUNPF081-00-VP administered at 60 mcg by repeat dosing at Day 0 and Week 16.
- VRC-FLUDNA082-00-VP vaccine administered at 4 mg at Day 0 and VRC-FLUNPF081-00-VP administered at 60 mcg at Week 16.

Secondary objectives include the evaluation of antibody responses to two prime-boost vaccine regimens in healthy adults at two weeks after the boost vaccine for each treatment and age group.

Exploratory objectives include the evaluation of the magnitude, the specificity, and the frequency of H2-specific T-cell, B-cell responses and antibody responses at indicated time points throughout the 40 weeks of the study.

6.3 Endpoints

6.3.1 Primary Endpoints: Safety

Assessment of product safety will include clinical observation and monitoring of hematological and chemical parameters. Reactogenicity will be closely monitored for 7 days after each injection and safety evaluated by clinical visits throughout 40 weeks. See Section 4.2 and Appendix II for details and specified time points.

The following parameters will be assessed for all study groups:

- Occurrence of solicited local reactogenicity symptoms for 7 days following each injection
- Occurrence of solicited systemic reactogenicity symptoms for 7 days following each injection
- Change from baseline in safety laboratory measures
- Occurrence of AEs of all severities through the "Week 4" visit after each injection
- Occurrence at any time throughout the study of SAEs or new chronic medical conditions that require ongoing medical management.

6.3.2 Secondary Endpoints: Immunogenicity

The principal immunogenicity endpoints are magnitude and frequency of antibody responses measured by neutralization assay and/or HAI assay at Week 0, at 4 weeks (W4) after the prime vaccination, and at 2 weeks (W18) after the boost vaccination.

6.3.3 Exploratory Endpoints: Immunogenicity

The magnitude, the specificity, and the frequency of H2-specific neutralizing antibodies that are four-fold greater than baseline at 2 weeks after the boost for each group will be evaluated.

Evaluation of antibody and T cell responses at time points throughout the study will be completed as an exploratory evaluation.

For the H2 neutralization assay, a positive response for a subject is defined as the subject achieving a four-fold rise in the H2-specific neutralization antibody titer from baseline.

6.4 Sample Size and Accrual

Recruitment will target 50 healthy adult participants 18 to 70 years of age. Adults born between 1966 and 1969 will be excluded from the trial.

6.4.1 Power Calculations for Safety

The goal of the safety evaluation for this study is to identify safety concerns associated with injections of the investigational vaccines. Primary sample size calculations for safety are expressed in terms of the ability to detect serious adverse experiences. Other sample size calculations for comparing the two vaccination groups on adverse experiences are similar to the calculations for immunogenicity (Section 6.4.3).

The ability of the study to identify SAEs will be expressed in terms of the probability of observing a certain number of SAEs. Useful values are the minimum true rate such that the probability of observing at least one event is at least 90%, and the maximum true rate such that the probability of not observing any event is at least 90%.

For Part I (*dose-escalation*) within each group (n=5), there is a 90% chance to observe at least 1 SAE if the true rate is at least 0.369 and over 90% chance to observe no SAE if the true rate is less than 0.021.

For Part II (*prime-boost*) within each group (n=10), there is over a 90% chance to observe at least 1 SAE if the true rate is at least 0.206 and over a 90% chance to observe no SAE if the true rate is no more than 0.010.

Probabilities of observing 0 or more than 1 SAE within each group are presented in **Table 6-1** for a range of possible true event rates. These calculations provide a complete picture of the sensitivity of this study design to identify potential safety problems with the vaccine.

Table 6-2 gives the upper and lower bounds for 95% exact binomial confidence intervals of the true SAE rate at all possible numbers of events within each group (n=5 and n=10).

For Part I (n=5): If none of the 5 participants receiving the vaccines experience SAEs, the 95% exact 2-sided upper confidence bound for the SAE would range from 0 to 0.52.

For Part II (n=10): If none of the 10 participants receiving the vaccines experience SAEs, the 95% exact 2-sided upper confidence bound for the SAE rate is 0.308.

Table 6-1: Probability of Events for Different Safety and Immunogenicity Scenarios

	Part I (*dose-escalation*): N=5		Part II (*prime-boost*): N=10	
True Event Rate	Pr (observing 0 event)	Pr (observing more than 1 event)	Pr (observing 0 event)	Pr (observing more than 1 event)
0.01	0.951	0.001	0.904	0.004
0.02	0.904	0.004	0.817	0.016
0.05	0.774	0.023	0.599	0.086
0.1	0.590	0.081	0.349	0.264
0.15	0.444	0.165	0.197	0.456
0.2	0.328	0.263	0.107	0.624
0.3	0.168	0.472	0.028	0.851
0.4	0.078	0.663	0.006	0.954
0.5	0.031	0.813	0.001	0.989

Table 6-2: 95% Confidence Intervals for the True Rate at All Possible Observed Rates within a Group (n=5 and n=10)

	Part I: N=5<br>95% Confidence Interval				Part II: N=10<br>95% Confidence Interval	
Observed Rate	Lower Bound	Upper Bound		Observed Rate	Lower Bound	Upper Bound
0/5	0	0.522		0/10	0	0.308
1/5	0.005	0.716		1/10	0.003	0.445
2/5	0.053	0.853		2/10	0.025	0.556
3/5	0.147	0.947		3/10	0.067	0.652
4/5	0.284	0.995		4/10	0.122	0.738
5/5	0.478	1		5/10	0.187	0.813
				6/10	0.262	0.878
				7/10	0.348	0.933
				8/10	0.444	0.975
				9/10	0.555	0.997
				10/10	0.692	1

6.4.2 Power Calculations for Immunogenicity

Table 6-2 is applicable to the immunogenic response rates, and gives the exact 95% confidence interval of the true response rate over possible number of responses out of the 5 and 10 subjects.

Table 6-1 gives the probabilities of observing 0 or more than 1 response over a range of underlying response rates.

6.4.3 Power for Comparison

As exploratory analyses, we will compare the four groups in Part II of the study for possible differences in immunogenicity; a simple comparison on the positive response rate will be used. **Table 6-3** gives the power of Fisher's exact test to compare any two schedules over a range of possible response rates. It indicates that a comparison between two groups is not powered by the study design with 10 samples per group. **Table 6-3** also gives the power of Fisher's exact test when the age cohorts within each group are combined, resulting in a comparison between two groups with 20 samples per group. It indicates that with age cohorts combined, the comparison would be powered for larger differences between groups.

Table 6-3: Power to Detect Difference in Response Rates between the groups in Part II by Fisher's Exact Test, Where Both Groups are of Size n=10 or n=20

	Group 4A (N=10)						
Group 3A (N=10)		0.1	0.2	0.3	0.4	0.5	0.6
	0.1		1	6	15	30	47
	0.2	1		2	6	13	25
	0.3	6	2		2	5	12
	0.4	15	6	2		2	5
	0.5	30	13	5	2		2
	0.6	47	25	12	5	2	
	Group 4B (N=20)						
Group 3B (N=20)		0.1	0.2	0.3	0.4	0.5	0.6
	0.1		7	24	49	74	91
	0.2	7		6	19	41	65
	0.3	24	6		6	16	35
	0.4	49	19	6		5	14
	0.5	74	41	16	5		5
	0.6	91	65	35	14	5	

6.5 Statistical Analysis

All statistical analyses will be performed using Statistical Analysis System (SAS), R, or S-Plus statistical software. No formal multiple comparison adjustments will be employed for safety endpoints or secondary endpoints.

6.5.1 Analysis Variables

The analysis variables consist of baseline variables and safety variables for primary and secondary objective analyses.

6.5.2 Baseline Demographics

Baseline characteristics including demographics and laboratory measurements will be summarized using descriptive statistics.

6.5.3 Safety Analysis

6.5.3.1 Reactogenicity

The number and percentage of participants experiencing each type of reactogenicity sign or symptom will be tabulated by severity. For a given sign or symptom, each participant's reactogenicity will be counted once under the maximum severity for all assessments.

6.5.3.2 Adverse Experiences

Adverse experiences are coded into Medical Dictionary for Regulatory Activities (MedDRA) preferred terms. The number and percentages of participants experiencing each specific AE will be tabulated by severity and relationship to treatment. For the calculations in these tables, each participant's adverse experience will be counted once under the maximum severity or strongest recorded causal relationship to treatment.

A complete listing of adverse experiences for each participant will provide details including severity, relationship to treatment, onset, duration and outcome.

6.5.3.3 Local Laboratory Values

Boxplots of local laboratory values will be generated for baseline values and for values measured during the course of the study. Each boxplot will show the 1st quartile, the median, and the 3rd quartile. Outliers, or values outside the boxplot, will also be plotted. If appropriate, horizontal lines representing boundaries for abnormal values will be plotted.

6.5.4 Immunogenicity Analysis

The statistical analysis for immunogenicity will employ the intent-to-treat principle whereby all data from enrolled subjects will be analyzed according to the group assignment. However, if during immune assessment on stored samples, a subject is found to have a positive antibody response at baseline, the vaccine immune responses assessment for these subjects will not be included in the final immunogenicity analysis. If needed, a per-protocol analysis will be performed as secondary analysis where subjects will be analyzed according to their actual vaccination scheme if it is different from the assigned or up to the last visit in the study if there are early dropouts. The study is not designed to power the comparison in immune responses between vaccine dosages.

If assay data are qualitative (i.e., positive or negative) then analyses will be performed by tabulating the frequency of positive response for each assay at each time point that an assessment is performed. Binomial response rates will be presented with their corresponding exact 95% confidence interval estimates.

Some immunologic assays have underlying continuous or count-type readout that is often dichotomized into responder/non-responder categories. For these assays, graphical and tabular summaries of the underlying distributions will be made. These summaries may be performed on transformed data (e.g., log transformation) to better satisfy assumptions of symmetry and homoscedasticity.

6.5.5 Missing Data

Missing responses will be assumed to be missing completely at random. Analyses will include all samples available at each study time point.

6.5.6 Interim Analyses

Safety Reviews: The PSRT will review safety data routinely throughout the study. The study will utilize both electronic database features and reviews by designated safety review personnel to identify in a timely manner if any of the safety pause rules of the study are met.

Immunogenicity Review: The analyses of immunogenicity may be performed when the HAI assays of samples are collected at 4 weeks after the prime vaccination and at 2 weeks after the boost vaccine. This may occur prior to completion of safety follow-up visits or collection of data for secondary and exploratory immunogenicity endpoints. Such an analysis would constitute the final analysis for the primary immunogenicity endpoint, so sample size adjustments are not required. Reports providing results by study group may be provided to VRC solely for the purpose of informing decisions related to future trials in a timely manner. The results should in no way influence the conduct of the VRC 316 trial in terms of early termination or later safety or immunogenicity endpoint assessments. Analyses of secondary and exploratory immunogenicity assays may also be performed as data become available.

6.6 Intervention Assignments

For Part I (*dose-escalation*): Subjects between 18-47 years will be assigned to the dose group that is open to accrual at the time of enrollment. When a subject is enrolled into a group but does not begin study injections or if a subject withdraws, a new eligible subject may be enrolled into the same group in order to complete the safety data set as planned.

For Part II (*prime-boost*): Subjects will be stratified by age and randomized equally into Group 3 or Group 4.

The subject and the study clinicians will be informed on the subject's randomization assignment upon completing of enrollment in Advantage Electronic Data Collection (AdEDC) system.

To decrease the potential for participant dropouts during the period between randomization and initial vaccination, randomization will occur on Day 0 after the study consent is signed and eligibility is confirmed. If subjects accrued to a study group do not complete the number of vaccinations and follow-up duration specified, then additional subjects may be accrued in that group.

7. PHARMACY AND VACCINE ADMINISTRATION PROCEDURES

The study groups and study agent dosing schedule are shown in **Table 2** in **Section 4**. Refer to the IB for further information about the investigational study products.

7.1 Study Product

The study includes two investigational vaccines described as follows:

- VRC-FLUNPF081-00-VP at 180 mcg/mL. Vials contain 0.7 mL of a clear, colorless, isotonic buffered (pH=7.2) sterile solution. The formulation buffer consists of 20mM Sodium Phosphate, 100mM Sodium Chloride and 5% Sucrose. Vials are intended for single use only and do not contain a preservative.
- VRC-FLUDNA082-00-VP at 4 mg/mL. DNA A/Sing vaccine is supplied as a 3 mL glass vial containing a clear, colorless, isotonic, PBS-buffered (pH=7.2), sterile solution. Vials are intended for single use only and do not contain a preservative. Each vial (4 mg/mL) contains a volume of 1.2 mL (4.8 mg).

- VRC-PBSPLA043-00-VP (PBS), diluent, is a clear, colorless, sterile solution at pH 7.2, aseptically filled to a volume of 1.2 mL in a 3 mL glass vial. Vials are intended for single use only and do not contain preservative. Vials must not be refrozen or reused after thawing.

7.2 Study Product Presentation and Storage

7.2.1 Study Product Labels

VRC-FLUNPF081-00-VP and VRC-FLUDNA082-00-VP will have specific product information (e.g., part number, lot number, fill volume, storage temperature) included on the product vial labels. The label will contain an Investigational Use Statement ("Limited by Federal Law to Investigational Use"), and manufacturer information.

7.2.2 Study Product Storage

VRC-FLUNPF081-00-VP and VRC-FLUDNA082-00-VP vials will be shipped within the recommended temperature range using appropriate shipping configurations, to the study pharmacist or designee.

VRC-FLUNPF081-00-VP (HA-F A/Sing): Vials of vaccine are stored until use at -35°C to -15°C in a qualified, continuously monitored, temperature-controlled freezer. As freezer temperatures may fluctuate, a temperature range of -45°C to -15°C is acceptable.

VRC-FLUDNA082-00-VP (DNA A/Sing): Vials of vaccine are stored until use at -35°C to -15°C in a qualified, continuously monitored, temperature-controlled freezer. As freezer temperatures may fluctuate, a temperature range of -45°C to -10°C is acceptable. Storage below -45ºC is not permitted because of the stopper limitation.

VRC-PBSPLA043-00-VP (PBS): Vials of PBS are stored until use at the target temperature of -35°C to -15°C in a qualified, continuously monitored, temperature-controlled freezer. As freezer temperatures may fluctuate, a temperature range of -45°C to -10°C is acceptable based upon historic stability data from studies of similar products.

7.2.3 Temperature excursions

If deviations in storage temperature occur from the normal allowance for the pharmacy freezer, the site pharmacist or designee must report the storage temperature excursion promptly to the PI and IND Sponsor. The excursion must be evaluated and investigated and action must be taken to restore and maintain the desired temperature limits. Pending the outcome of the investigation, the IND Sponsor will notify the pharmacist if continued clinical use of the product is acceptable.

7.3 Preparation of Study Products for Injection

This section describes how the site pharmacist will prepare study injections. Clinician instructions on how to select an arm and administer an injection are in Section 4.2.4**.** Refer to the group assignment for the study subject to select the proper vial type.

For all study groups, the prepared syringe will be labeled with the subject identifier and the date and time after which the preparation may not be used. Allow the vial to equilibrate to room temperature (15 to 30º C). Swirl the contents gently. Filled syringes should be kept at room temperature and out of direct sunlight until the product is administered. All injections must be administered within 8 hours after removing the vaccine vial from the freezer.

7.3.1 Preparation of VRC-FLUNPF081-00-VP to be administered via Needle and Syringe Injection

Subjects will receive the VRC-FLUNPF081-00-VP vaccine via needle and syringe injection. The following instructions apply for VRC-FLUNPF081-00-VP vaccine preparation:

Group 1 (*20 mcg*):

1. Thaw 1 vial of HA-F A/Sing at room temperature until all ice crystals have melted. Swirl gently to mix.
2. Thaw 1 vial of PBS diluent at room temperature until all ice crystals have melted. Swirl gently to mix.
3. Withdraw 0.11 mL of HA-F A/Sing into the syringe.
4. Withdraw 0.2 mL of PBS diluent directly into the syringe.
5. Invert syringe 5x to mix.

Group 2 and **Groups 4A/B** (*60 mcg, prime and boost*); **Groups 3A/B** (*60 mcg,* ***ONLY boost***):

1. Thaw 1 vial of HA-F A/Sing at room temperature until all ice crystals have melted. Swirl gently to mix.
2. Withdraw 0.33 mL of HA-F A/Sing into the syringe.

Please refer to the IB for more information on vaccine preparation for injection.

7.3.2 Preparation of VRC-FLUDNA082-00-VP to be administered via PharmaJet Injection

For subjects randomized to **Group 3A/B** (PharmaJet), remove a single dose vial of the DNA vaccine 4 mg/mL from the freezer. Using aseptic technique, withdraw 0.5 mL of the DNA vaccine from the vial into the PharmaJet 0.5 mL needle-free syringe and remove all air bubbles. Two 0.5 mL injections of the 4 mg/mL preparation will be administered for each 4mg dose with the PharmaJet.

7.4 STUDY PRODUCT ACCOUNTABILITY

7.4.1 Documentation

The study pharmacist or designee will be responsible for maintaining an accurate record of the codes, inventory, and an accountability record of vaccine supplies for this study. Electronic documentation as well as paper copies will be used.

7.4.2 Disposition

The empty vials and the unused portion of a vial will be discarded in a biohazard containment bag and incinerated or autoclaved following the injection. Any unopened vials that remain at the end of the study will be discarded at the discretion of the VRC in accordance with policies that apply to investigational products. Partially used vials or expired prepared doses cannot be administered to other subjects nor used for *in vitro* experimental studies and will be discarded as indicated above.

8. HUMAN SUBJECT PROTECTIONS AND ETHICAL OBLIGATIONS

This research study will be conducted in compliance with the protocol, International Council for Harmonisation Good Clinical Practices (ICH-GCP) guidance, and all applicable regulatory requirements.

8.1 Institutional Review Board

A copy of the protocol, ICF, other written subject information, and any advertising material will be submitted to the NIAID IRB for written approval prior to use.

The PI must submit and, where necessary, obtain approval from the IRB for all subsequent protocol amendments and changes to the ICF. The PI will notify the IRB of unanticipated problems, non-compliance, deviations from the protocol, and serious SAEs as described in Section 5.4.

The investigator will be responsible for obtaining IRB approval of the annual Continuing Review throughout the duration of the study.

8.2 Subject Identification and Enrollment of Study Participants

Study subjects will be recruited through on-site and off-site advertising done for the screening protocol, VRC 500 (11-I-0164). All study activities will be carried out at the NIH CC. Effort will be made to include women and minorities in proportions similar to that of the community from which they are recruited.

8.2.1 Participation of Children

Children are not eligible to participate in this clinical trial because the investigational vaccine regimens have not been previously evaluated in adults. If the product is assessed as safe and immunogenic, other protocols designed for children may be conducted in the future.

8.2.2 Participation of NIH Employees

NIH employees and members of their immediate families may participate in this protocol. NIH staff may be a vulnerable class of subjects. The VRC will follow the Guidelines for the Inclusion of Employees in NIH Research Studies and will give each employee a copy of the "NIH Information Sheet on Employee Research Participation" and a copy of the "Leave Policy for NIH Employees Participating in NIH Medical Research studies."

Neither participation nor refusal to participate will have an effect, either beneficial or adverse, on the participant's employment or work situation. The NIH information sheet regarding NIH employee research participation will be distributed to all potential subjects who are NIH employees. The employee subject's privacy and confidentiality will be preserved in accordance with NIH CC and NIAID policies. For NIH employee subjects, consent will be obtained by an individual who is independent of the employee's team. If the individual obtaining consent is a co-worker to the subject, independent monitoring of the consent process will be included through the Bioethics Consultation Service. Protocol study staff will be trained on obtaining potentially sensitive and private information from co-workers or subordinates.

8.3 Informed Consent

The study ICF, , describes the investigational products to be used and all aspects involved in protocol participation.

Before a subject's participation in the study, it is the investigator's responsibility to obtain written informed consent from the subject, after adequate explanation of the aims, methods, anticipated benefits, and potential hazards of the study and before any protocol-specific procedures or study products are administered.

An IRB-approved AoU quiz, intended to assist in the evaluation of the subject's understanding of this study, is administered as part of the consent process.

The acquisition of informed consent will be documented in the subject's medical records, as required by 21 CFR 312.62, and the ICF will be signed and personally dated by the subject and by the person who conducted the informed consent discussion. The original signed ICF will be placed in the medical record and a copy of the signed ICF will be provided to the subject.

8.4 Subject Confidentiality

The investigator must ensure that the subject's anonymity is maintained and will ensure that no information identifying the subject will be released to any unauthorized party. Subjects will not be identified in any reports on this study. All records will be kept confidential to the extent provided by federal, state and local law. Medical records are made available for review when required by the FDA or other authorized users, such as the vaccine manufacturer, only under the guidelines set by the Federal Privacy Act. Direct access includes examining, analyzing, verifying, and reproducing any records and reports that are important to the evaluation of the study. The investigator is obligated to inform the subjects that the above-named representatives will review their study-related records without violating the confidentiality of the subjects. Stored study research samples are labeled by a code (such as a number) that only the study team can link to the subject. The requirement to maintain subject confidentiality is included in the study ICF.

8.5 Risks and Benefits

8.5.1 Risks of the VRC-FLUNPF081-00-VP vaccine

This is the first study in humans of this investigational VRC-FLUNPF081-00-VP vaccine. This product is derived from *H. pylori* ferritin and not human ferritin, for this reason it is not expected to interfere with iron storage, transport or iron serum levels [13].

Potential side effects resulting from IM injection include stinging, arm discomfort, redness of the skin or mild bruising at vaccine injection sites.

Subjects may exhibit general signs and symptoms associated with administration of a vaccine, including fever, chills, rash, aches and pains, nausea, headache, dizziness and fatigue. These side effects will be monitored, but are generally short term, mild to moderate severity and usually do not require treatment.

There may be other unknown side effects.

8.5.2 Risks of the VRC-FLUDNA082-00-VP Vaccine

This is the first study in humans of the investigational DNA vaccine, VRC-FLUDNA082-00-VP. The risks noted are based on risks of vaccines in general, and results of previous studies with other investigational DNA vaccines.

Potential side effects resulting from IM injection with PharmaJet include stinging, arm discomfort, redness of the skin, mild bruising or a small laceration at vaccine injection sites.

There is also a small chance that the needle-free device could not work properly. As of June 26, 2017, there have been 8 out of 426 (1.9%) injections that involved device malfunction in VRC 705 Part A. This includes the syringe breaking and study product spraying during administration. No harm to subjects has occurred when this happened. Investigations are ongoing

to determine the factors leading to malfunction. As a result, Subjects in Group 3A/B and clinicians administering the vaccine will be asked to wear safety glasses when using PharmaJet.

Subjects may exhibit general signs and symptoms associated with administration of a vaccine injection, including fever, chills, rash, aches and pains, nausea, headache, dizziness and fatigue.

These side effects will be monitored, but are generally short-term, mild to moderate severity and usually do not require treatment.

In previous VRC DNA vaccine studies, placebo and vaccine recipients were noted to have occasional asymptomatic and self-limited changes in laboratory tests such as temporary drop in white blood cell count. Urticaria has been reported as possibly related to DNA vaccines in rare cases.

Investigational DNA vaccines administered via needleless injection device have been associated with mild skin lesions (0.5-1.0 cm diameter) at the vaccination site. In these cases a small scab formed within 1-2 weeks after immunization and came off after a few days. The skin healed without treatment within a few weeks. We do not know if the side effects from PharmaJet will be the same as Biojector. There may be other unknown side effects.

8.5.3 Other Risks

Risks of Specimen Collections:

- *Blood drawing:* The risks of blood sample collection are minimal and consist of mild discomfort at the sample collection site. The procedure may cause pain, bruising, fainting, and, rarely, infection at the site where the blood is taken.

- *Apheresis*: donations may cause pain, bruising, and discomfort in the arms where the needles are placed. It may also cause chills, nausea, heartburn, mild muscle cramps and tingling sensation around the mouth or in the fingers, however this can usually be relieved by slowing or temporarily interrupting the apheresis or taking a calcium containing antacid, such as Tums®. Other possible side effects are anxiety, vomiting and lightheadedness. Temporary lowering of the blood pressure may develop. There is the rare possibility of infection, fainting or seizure. Very rarely a nerve problem at the needle placement site may occur. Also, very rarely, a machine malfunction may occur, resulting in the loss of about one unit of blood. There may be additional risks of apheresis that are unknown at this time.

Risks of study vaccines on the fetus or nursing infant:

We do not know the possible effects of the study vaccines on the fetus or nursing infant. Women of reproductive potential will be required to agree to use birth control beginning 21 days prior to enrollment and continuing through end of study.

Because this is a research study, women of reproductive potential will be tested for pregnancy prior to administration of each study injection and asked to notify the site immediately upon learning of a pregnancy during this study. In the case of pregnancy, subjects will no longer receive additional vaccine product and continue to be followed for safety. Research sample collections will be discontinued for pregnant women. The subject will be contacted to ask about the outcome of a pregnancy that begins during the study.

Risks of new diagnoses:

It is possible that the standard medical tests performed as part of this research protocol will result in new diagnoses. Depending upon the medical findings and consequences of being provided with the new medical information about health status, the study subject may view this aspect of study participation as either a risk or a benefit. Any such information will be shared and discussed with the subject and, if requested by the subject, will be forwarded to the subject's primary health care provider for further workup and management.

8.5.4 Study Benefits

Study subjects will not receive direct health benefit from study participation. This protocol is not designed to provide treatment for any condition. Others may benefit from knowledge gained in this study that may aid in the development of an H2 (or universal) influenza virus vaccine. Neither of the two investigational vaccines are expected to provide protection from influenza.

8.6 Plan for Use and Storage of Biological Samples

The plan for use and storage of biological samples from this protocol is as outlined in the following sections.

8.6.1 Use of Samples, Specimens and Data

Samples, specimens and data collected under this protocol may be used to conduct protocol related safety and immune response evaluations, exploratory laboratory evaluations related to the type of infection the study product was designed to prevent, exploratory laboratory evaluations related to vaccine or infectious disease research in general and for research assay validation.

Genetic testing may be performed in accordance with the genetic testing information that was included in the study ICF. HLA testing may be done in association with identifying factors linked with the immune response development or progression of infections.

Additional optional genetic testing, including transcriptome sequencing may be done on collected specimens in an effort to assess the expression of genes involved in the immune response to vaccination.

Results of genetic testing may have psychological implications for patients such as revelations regarding future health risks, incurable conditions, and/or information contradictory to stated biological relationships. Genetics counseling and advice will be available from the NIH to help subjects at all study sites with the implications of findings, where appropriate.

Following genetic testing, the data will be shared in a controlled-access public database for other investigators to benefit from it (e.g. the Database of Genotypes and Phenotypes dbGAP). However no personal, identifiable information will be shared in this process as the results will only be shared with a code.

Other optional analysis, including proteome, lipidome, metabolome, and exosome may be done on collected specimens to evaluate some proteins, lipids, metabolites, and low molecular weight molecules involved in the immune response to vaccination.

8.6.2 Storage and Tracking of Blood Samples and Other Specimens

All of the stored study research samples are labeled by a code that only the site can link to the subject. Samples are stored at the VITL, Gaithersburg, MD or VRC Laboratories in Building 40, Bethesda, MD, which are both secure facilities with limited access. Data will be kept in

password-protected computers. Only investigators or their designees will have access to the samples and data. Samples will be tracked in the Laboratory Information Management System (LIMS) database or using another software designed for this purpose (e.g., Freezerworks).

8.6.3 Disposition of Samples, Specimens and Data at Completion of the Protocol

In the future, other investigators (both at NIH and outside) may wish to study these samples and/or data. IRB approval must be sought prior to any sharing of samples. Any clinical information shared about those samples would similarly require prior IRB approval. The research use of stored, unlinked or unidentified samples may be exempt from the need for prospective IRB review and approval. Exemption requests will be submitted in writing to the NIH Office of Human Subjects Research, which is authorized to determine whether a research activity is exempt.

At the time of protocol termination, samples will remain in the VITL facility or VRC laboratories or, after IRB approval, transferred to another repository. Regulatory oversight of the stored samples and data may be transferred to a stored samples protocol as part of the IRB-approved termination plan. Data will be archived by the VRC in compliance with requirements for retention of research records, or after IRB and study sponsor approval, it may be either destroyed or transferred to another repository.

8.6.4 Loss or Destruction of Samples, Specimens or Data

The NIH Intramural Protocol Deviation definition related to loss of or destruction of samples or data will be followed. Any loss or unanticipated destruction of samples (for example, due to freezer malfunction) or data (for example, misplacing a printout of data with identifiers) that compromises the scientific integrity of the study will be reported to the IRB in accordance with institutional policies. The PI will also notify the IRB if the decision is made to destroy the remaining samples.

8.7 Compensation

Subjects will be compensated for time and inconvenience in accordance with the standards for compensation of the Clinical Research Volunteer Program. The compensation per visit will be $275 for visits that include injections and $175 for clinic visits which include a blood draw. The compensation for any clinic visit that does not include a blood draw or procedure will be $75 and compensation for timely completion of electronic diary card will be $25. Compensation will be $250 for apheresis if performed. The total compensation for the subject is based on the number of study clinic visits, injections completed and if optional research blood collections are performed.

Subjects will receive compensation by direct deposit within approximately 1 or 2 weeks after each completed visit.
Compensation may need to be reported to the Internal Revenue Service (IRS) as taxable income.

The approximate total compensation is as follows:

Group	Without Apheresis	With Apheresis
Group 1	$1,525	NA
Group 2<br>Group 3A-B<br>Group 4A-B	$2,000	$2,075

8.8 Safety Monitoring

8.8.1 Protocol Safety Review Team

Close cooperation between the designated members of the Protocol Team will occur to evaluate and respond to individual AEs in a timely manner. The VRC designated Safety Officer for the day conducts a daily safety review of clinical data per VRC Standard Operating Procedures. The PSRT, comprised of the PI, Associate Investigators, Study Coordinator, Protocol Specialists, and other Study Clinicians, will review the summary study safety data reports on a weekly basis through 4 weeks after the last subject receives the final study injection. After this time, the PSRT will monitor the safety data reports on a monthly basis through completion of the last study visit.

9. ADMINISTRATIVE AND LEGAL OBLIGATIONS

9.1 Protocol Amendments and Study Termination

Protocol amendments may be made only with the prior approval of the IND sponsor representative, VRC, NIAID. Agreement from the PI must be obtained for all amendments to the protocol and the informed consent document. All study amendments will be submitted to the IRB for approval.

The VRC, NIAID IRB, Office of Human Research Protections, study PI, and FDA reserves the right to terminate the study. The PI will notify the IRB in writing of the study's completion or early termination.

9.2 Study Documentation and Storage

The PI will maintain a list of appropriately qualified persons to whom trial duties have been delegated.

Source documents are original documents, data, and records from which the subject's data are obtained. These include but are not limited to hospital records, clinical and office charts, laboratory and pharmacy records, microfiches, radiographs, and correspondence.

The PI and staff are responsible for maintaining a comprehensive and centralized filing system of all study-related (essential) documentation, suitable for inspection at any time by representatives from the VRC, IRB, FDA, and/or applicable regulatory authorities. Elements include:

- Subject files containing completed informed consent forms, and supporting copies of source documentation (if kept)
- Study files containing the protocol with all amendments, Investigator Brochures, copies of all correspondence with the IRB and VRC

In addition, all original source documentation must be maintained and be readily available.

All essential documentation should be retained by the institution for the same period of time required for medical records retention. The FDA requires study records to be retained for up to two years after marketing approval or refusal (21 CFR 312.62). No study document should be destroyed without prior written agreement between the VRC and the PI. Should the PI wish to assign the study records to another party or move them to another location, VRC must be notified in writing of the new responsible person and/or the new location.

9.3 Data Collection, Data Sharing and Protocol Monitoring

9.3.1 Data Collection

Clinical research data will be collected in a secure electronic web-based clinical data management system (CDMS) through a contract research organization, EMMES (Rockville, MD). Extracted data without patient identifiers will be sent to the Protocol Statistician for statistical analysis.

9.3.2 Data Sharing Plan

Data generated in this study will be shared as de-identified data in the government-funded public repository, www.ClinicalTrials.gov. Data may be shared prior to publication at approved public presentations or for collaborative development and will be shared at the time of publication or within 1 year of the primary completion date.

9.3.3 Source Documents

The site will maintain appropriate medical and research records for this trial, in compliance with ICH-GCP, regulatory and institutional requirements for the protection of confidentiality of subjects.

Source data are all information, original records of clinical findings, observations, or other activities in a clinical trial necessary for the reconstruction and evaluation of the trial. Examples of these original documents and data records include, but are not limited to, medical records, laboratory reports, pharmacy records and other research records maintained for the clinical trial.

9.3.4 Protocol Monitoring Plan

The NIAID and VRC or their authorized representatives are responsible for contacting and visiting the PI for the purpose of inspecting the facilities and, upon request, inspecting the various records of the trial, provided that subject confidentiality is respected.

Site investigators will allow the study monitors, the NIAID IRB, and the FDA to inspect study documents (e.g., consent forms, drug distribution forms, and case report forms) and pertinent hospital or clinic records for confirmation of the study data.

Site visits by study monitors will be made in accordance with the study monitoring plan to monitor the following: study operations, the quality of data collected in the research records, the accuracy and timeliness of data entered in the database, and to determine that all process and regulatory requirements are met. Study monitoring visits will occur as defined by the IND Sponsor approved monitoring plan.

9.4 Language

All written information and other material to be used by subjects and investigative staff must use vocabulary and language that are clearly understood.

9.5 POLICY REGARDING RESEARCH-RELATED INJURIES

The NIH CC will provide short-term medical care for any injury resulting from participation in this research. In general, the NIH, the NIH CC, or the U.S. Federal Government will provide no long-term medical care or financial compensation for research-related injuries.

10. REFERENCES

1. Subbarao, K., B.R. Murphy, and A.S. Fauci, *Development of effective vaccines against pandemic influenza.* Immunity, 2006. **24**(1): p. 5-9.
2. CDC. *Types of Influenza Viruses*. 2014 4/15/2014]; Available from: http://www.cdc.gov/flu/about/viruses/types.htm.
3. Taubenberger, J.K. and J.C. Kash, *Influenza virus evolution, host adaptation, and pandemic formation.* Cell Host Microbe, 2010. **7**(6): p. 440-51.
4. WHO. *Influenza (Seasonal) Fact Sheet*. 2009; Available from: http://www.who.int/mediacentre/factsheets/fs211/en/index.html.
5. Meltzer, M.I., N.J. Cox, and K. Fukuda, *The economic impact of pandemic influenza in the United States: priorities for intervention.* Emerg Infect Dis, 1999. **5**(5): p. 659-71.
6. Krause, J.C., et al., *Human monoclonal antibodies to pandemic 1957 H2N2 and pandemic 1968 H3N2 influenza viruses.* J Virol, 2012. **86**(11): p. 6334-40.
7. Tripp, R.A. and S.M. Tompkins, *Virus-vectored influenza virus vaccines.* Viruses, 2014. **6**(8): p. 3055-79.
8. CDC. *Vaccine Effectiveness - How Well Does the Flu Vaccine Work?* 2016 11/14/2016; Available from: https://www.cdc.gov/flu/about/qa/vaccineeffect.htm.
9. Lambert, L.C. and A.S. Fauci, *Influenza vaccines for the future.* N Engl J Med, 2010. **363**(21): p. 2036-44.
10. Krammer, F. and P. Palese, *Advances in the development of influenza virus vaccines.* Nat Rev Drug Discov, 2015. **14**(3): p. 167-82.
11. Neuzil, K.M. and R.A. Bright, *Influenza vaccine manufacture: keeping up with change.* J Infect Dis, 2009. **200**(6): p. 835-7.
12. Bai, L., et al., *Genome-wide comparison of ferritin family from Archaea, Bacteria, Eukarya, and Viruses: its distribution, characteristic motif, and phylogenetic relationship.* Naturwissenschaften, 2015. **102**(9-10): p. 64.
13. Kanekiyo, M., et al., *Self-assembling influenza nanoparticle vaccines elicit broadly neutralizing H1N1 antibodies.* Nature, 2013. **499**(7456): p. 102-6.
14. Yamashita, I., K. Iwahori, and S. Kumagai, *Ferritin in the field of nanodevices.* Biochim Biophys Acta, 2010. **1800**(8): p. 846-57.
15. Zhao, L., et al., *Nanoparticle vaccines.* Vaccine, 2014. **32**(3): p. 327-37.
16. Krammer, F., P. Palese, and J. Steel, *Advances in universal influenza virus vaccine design and antibody mediated therapies based on conserved regions of the hemagglutinin.* Curr Top Microbiol Immunol, 2015. **386**: p. 301-21.
17. Nabel, G.J., C.-J. Wei, and J.E. Ledgerwood, *Vaccinate for the next H2N2 pandemic now.* Nature, 2011. **471**(7337): p. 157-158.
18. Ledgerwood, J.E., et al., *DNA priming and influenza vaccine immunogenicity: two phase 1 open label randomised clinical trials.* Lancet Infect Dis, 2011. **11**(12): p. 916-24.

19. Ledgerwood, J.E., et al., *Prime-Boost Interval Matters: A Randomized Phase 1 Study to Identify the Minimum Interval Necessary to Observe the H5 DNA Influenza Vaccine Priming Effect.* J Infect Dis, 2013. **208**(3): p. 418-22.

20. Ledgerwood, J.E., et al., *DNA priming for seasonal influenza vaccine: a phase 1b double-blind randomized clinical trial.* PLoS One, 2015. **10**(5): p. e0125914.

21. Khurana, S., et al., *DNA Priming Prior to Inactivated Influenza A(H5N1) Vaccination Expands the Antibody Epitope Repertoire and Increases Affinity Maturation in a Boost-Interval-Dependent Manner in Adults.* J Infect Dis, 2013. **208**(3): p. 413-7.

22. Ledgerwood, J.E., et al., *Phase I Clinical Evaluation of Seasonal Influenza Hemagglutinin (HA) DNA Vaccine Prime Followed by Trivalent Influenza Inactivated Vaccine (IIV3) Boost.* Contemp Clin Trials, 2015.

23. Adam D. DeZure, et al., *Safety and Immunogenicity of H7 Influenza Prime-Boost Regimens in Healthy Adults*, in *National Foundation for Infectious Diseases*. 2016: Baltimore, MD.

24. Ledgerwood, J.E., et al., *Influenza virus H5 DNA vaccination is immunogenic by intramuscular and intradermal routes in humans.* Clinical and Vaccine Immunology, 2012. **19**(11): p. 1792-7.

25. Ledgerwood, J.E. and B.S. Graham, *DNA vaccines: a safe and efficient platform technology for responding to emerging infectious diseases.* Hum Vaccin, 2009. **5**(9): p. 623-6.

26. Cui, Z., *DNA vaccine.* Adv Genet, 2005. **54**: p. 257-89.

27. Wei, C.J., et al., *Induction of broadly neutralizing H1N1 influenza antibodies by vaccination.* Science, 2010. **329**(5995): p. 1060-4.

28. Wei, C.J., et al., *Elicitation of broadly neutralizing influenza antibodies in animals with previous influenza exposure.* Science Transl Med, 2012. **4**(147): p. 147ra114.

29. Ledgerwood, J.E., et al., *A West Nile virus DNA vaccine utilizing a modified promoter induces neutralizing antibody in younger and older healthy adults in a phase I clinical trial.* J Infect Dis, 2011. **203**(10): p. 1396-404.

30. Martin, J.E., et al., *A West Nile virus DNA vaccine induces neutralizing antibody in healthy adults during a phase 1 clinical trial.* J Infect Dis, 2007. **196**(12): p. 1732-40.

31. Graham, B.S., et al., *Phase 1 safety and immunogenicity evaluation of a multiclade HIV-1 DNA candidate vaccine.* J Infect Dis, 2006. **194**(12): p. 1650-1660.

32. Martin, J.E., et al., *A SARS DNA vaccine induces neutralizing antibody and cellular immune responses in healthy adults in a Phase I clinical trial.* Vaccine, 2008. **26**(50): p. 6338-43.

33. Martin, J.E., et al., *A DNA vaccine for Ebola virus is safe and immunogenic in a phase I clinical trial.* Clin Vaccine Immunol, 2006. **13**(11): p. 1267-77.

34. Tavel, J.A., et al., *Safety and immunogenicity of a Gag-Pol candidate HIV-1 DNA vaccine administered by a needle-free device in HIV-1-seronegative subjects.* J Acquir Immune Defic Syndr, 2007. **44**(5): p. 601-5.

35. Kibuuka, H., et al., *A phase 1/2 study of a multiclade HIV-1 DNA plasmid prime and recombinant adenovirus serotype 5 boost vaccine in HIV-Uninfected East Africans (RV 172).* J Infect Dis, 2010. **201**(4): p. 600-7.

36. Jaoko, W., et al., *Safety and immunogenicity study of Multiclade HIV-1 adenoviral vector vaccine alone or as boost following a multiclade HIV-1 DNA vaccine in Africa.* PLoS One, 2010. **5**(9): p. e12873.

37. Koup, R.A., et al., *Priming Immunization with DNA Augments Immunogenicity of Recombinant Adenoviral Vectors for Both HIV-1 Specific Antibody and T-Cell Responses.* PLoS One, 2010. **5**(2): p. e9015.

38. Churchyard, G.J., et al., *A Phase IIA Randomized Clinical Trial of a Multiclade HIV-1 DNA Prime Followed by a Multiclade rAd5 HIV-1 Vaccine Boost in Healthy Adults (HVTN204).* PLoS One, 2011. **6**(8): p. e21225.

39. Graham, B.S., et al., *DNA vaccine delivered by a needle-free injection device improves potency of priming for antibody and CD8+ T-cell responses after rAd5 boost in a randomized clinical trial.* PLoS One, 2013. **8**(4): p. e59340.

40. Dowd, K.A., et al., *Rapid development of a DNA vaccine for Zika virus.* Science, 2016.

41. Betts, M.R., J.P. Casazza, and R.A. Koup, *Monitoring HIV-specific CD8+ T cell responses by intracellular cytokine production.* Immunol Lett, 2001. **79**(1-2): p. 117-25.

42. Barouch, D.H., et al., *A human T-cell leukemia virus type 1 regulatory element enhances the immunogenicity of human immunodeficiency virus type 1 DNA vaccines in mice and nonhuman primates.* J Virol, 2005. **79**(14): p. 8828-34.

43. Sheets, R.L., et al., *Biodistribution of DNA Plasmid Vaccines against HIV-1, Ebola, Severe Acute Respiratory Syndrome, or West Nile Virus Is Similar, without Integration, despite Differing Plasmid Backbones or Gene Inserts.* Toxicol Sci, 2006. **91**(2): p. 610-619.

APPENDIX I: CONTACT INFORMATION

[redacted]<br><br>[redacted]<br><br>[redacted]<br><br>[redacted]<br><br>[redacted]	[redacted]<br><br>[redacted]<br><br>[redacted]<br><br>[redacted]<br><br>[redacted]

[redacted]<br><br>[redacted]<br><br>[redacted]<br><br>[redacted]	[redacted]<br><br>[redacted]<br><br>[redacted]<br><br>[redacted]<br><br>[redacted]

APPENDIX II: SCHEDULE OF EVALUATIONS

		VRC 500	VRC 316 Schedule of Evaluations PART I: Group 1								
**Visit Number**		*01	02	02A	03	04	05	06	08	10	11
**Week of Study**		-12 to 0	W0	W1	W1	W2	W4	W16	W18	W28	W40
**Day of Study**		-84 to 0	[1]D0	D1	D6	D14	D28	D112	D126	D196	D280
**Clinical**	Tube										
*VRC 500 Screening Consent		X									
VRC 316 AoU; Consent			X								
[2]Physical exam for eligibility, height /weight/ vitals at screening; vital signs and targeted exam (as needed) other visits.		X	X		X	X	X	X	X	X	X
Medical history targeted to eligibility at screening; then interim medical history		X	X		X	X	X	X	X	X	X
[3]Study Product Administration: **Part I, Group 1**			**HA-F A/Sing**								
Phone evaluation (clinic visit as needed)				X							
Begin diary card			X								
[4]Pregnancy test: urine or serum		X	X				X				X
[4]Pregnancy prevention counseling/ Reproductive Information Form		X	X				X				X
CBC with differential	EDTA	3	3			3	3				3
Iron		X					X				
Total bilirubin, AST, ALT, and ALP	GLT	4	4			4	4				4
Creatinine		X	X		4						
[5]HLA type	EDTA						20				
HIV (other tests, if needed)	SST	4									
**Research Samples**											
H. pylori ferritin antibody and Human ferritin antibody	SST	X					X				
Antibody assays. Serum storage	SST	32	32		32		32	32	16	32	32
Immunological assays (T cell and antibody responses). PBMCs and plasma storage	EDTA	80	80		80		80	80	40	80	80
**Daily Volume (mL)**		123	119	0	116	7	139	112	56	112	119
**Max. Cumulative Volume (mL)**		123	242	242	358	365	504	616	672	784	903

* VRC 500: Most screening evaluations must be no more than 84 days prior to Day 0 to be used for eligibility (pregnancy test from Day 0 must be used for eligibility). If clinical assessment on Day 0 suggests significant changes may have occurred since screening, then physical examination & laboratory studies done on Day 0 are used for eligibility.

[1] Day 0=day of enrollment and first vaccine injection. Day 0 evaluations prior to first injection are the baseline for assessing adverse events subsequently.

[2] Screening visit includes physical exam with vital signs. At other visits, physical examination is done if indicated by interim history or laboratory test results. Otherwise only blood pressure (BP), pulse, and temperature are required.

[3] Study Product Administration: Part I: Group 1 will receive HA-F A/Sing 20 mcg IM at Day 0. Complete post vaccination evaluations (BP, pulse, temperature, respiration and injection site assessment) at 1 hour or longer after study injection.

[4] Negative pregnancy test results must be confirmed for women of reproductive potential prior to each study injection.

[5] HLA type blood sample is collected once at any time-point in the study and is shown as a Visit 05 evaluation for convenience; however, if HLA type is already available in the medical record, it does not need to be repeated. HLA type may also be obtained from a frozen sample.

Visit windows: Schedule Visits 02A through 11 with respect to Day 0; the following visit windows apply: Visits 02A (+1 day). Visit 03 (±1 day). Visits 04 and 05 (±2 days). Visits 06 (± 7 days). Visit 08 (±14 days). Visit 10 and 11 (± 14 days).

		VRC 500	VRC 316 Schedule of Evaluations PART I: Group 2											
**Visit Number**		*01	02	02A	03	04	05	06	06A	07	08	09	10	11
**Week of Study**		-12 to 0	W0	W1	W1	W2	W4	W16	W17	W17	W18	W20	W28	W40
**Day of Study**		-84 to 0	[1]D0	D1	D6	D14	D28	D112	D113	[6]D118	D126	D140	D196	D280
**Clinical**	Tube													
*VRC 500 Screening Consent		X												
VRC 316 AoU; Consent			X											
[2]Physical exam for eligibility, height /weight/ vitals at screening; vital signs and targeted exam (as needed) other visits.		X	X		X	X	X	X		X	X	X	X	X
Medical history targeted to eligibility at screening; then interim medical history		X	X		X	X	X	X		X	X	X	X	X
[3]Study Product Administration: **Part I**, **Group 2**			**HA-F A/Sing**					**HA-F A/Sing**						
Phone evaluation (clinic visit as needed)				X					X					
Begin diary card			X											
[4]Pregnancy test: urine or serum		X	X				X	X			X	X		X
[4]Pregnancy prevention counseling/ Reproductive Information Form		X	X				X	X			X	X		X
CBC with differential	EDTA	3	3			3	3	3				3		3
Iron		X					X					X		
Total bilirubin, AST, ALT, and ALP	GLT	4	4			4	4	4				4		4
Creatinine		X	X		4									
[5]HLA type	EDTA						20							
HIV (other tests, if needed)	SST	4												
**Research Samples**														
H. pylori ferritin antibody and Human ferritin antibody	SST	X					X					X		
Antibody assays. Serum storage	SST	32	32		32		32	32		32	32	32	32	32
Immunological assays (T cell and antibody responses). Multi-OMICS analysis. PBMCs and plasma storage	EDTA	80	80		80		80	80		[6]80	[7]120 or Apheresis (6)	80	80	80
**Daily Volume (mL)**		123	119	0	116	7	139	119	0	112	152	119	112	119
**Max. Cumulative Volume (mL)**		123	242	242	358	365	504	623	623	735	887	1006	1118	1237

* VRC 500: Most screening evaluations must be no more than 84 days prior to Day 0 to be used for eligibility (pregnancy test from Day 0 must be used for eligibility). If clinical assessment on Day 0 suggests significant changes may have occurred since screening, then physical examination & laboratory studies done on Day 0 are used for eligibility.

[1] Day 0=day of enrollment and first vaccine injection. Day 0 evaluations prior to first injection are the baseline for assessing adverse events subsequently.

[2] Screening visit includes physical exam with vital signs. At other visits, physical examination is done if indicated by interim history or laboratory test results. Otherwise only blood pressure (BP), pulse, and temperature are required.

[3] Study Product Administration: Part I: Group 2 will receive HA-F A/Sing 60 mcg IM at Day 0 and at Week 16. Complete post vaccination evaluations (BP, pulse, temperature, respiration and injection site assessment) at 1 hour or longer after each study injection.

[4] Negative pregnancy test results must be confirmed for women of reproductive potential prior to each study injection.

[5] HLA type blood sample is collected once at any time-point in the study and is shown as a Visit 05 evaluation for convenience; however, if HLA type is already available in the medical record, it does not need to be repeated. HLA type may also be obtained from a frozen sample.

[6] Visit 07: Two tubes of PBMCs collected in EDTA tubes will be sent to Building 40, while the remainder of the blood collected will be sent to VITL.

[7] If optional apheresis occurs, ONLY draw 32mL in SST (DO NOT draw 120 ml in EDTA tubes). For women of reproductive potential, pregnancy test must be negative within 72 hours prior to apheresis procedure.

Visit windows: Schedule Visits 02A through 06 with respect to Day 0; Schedule Visits 06A through 11 with respect to Visit 06. The following visit windows apply: Visits 02A and 06A (+1 day). Visit 03 and 07 (±1 day). Visits 04, 05 and 09 (±2 days). Visit 06 (±28 days). Visits 08 (±3 days). Visits 10 and 11 (±14 days).

		VRC 500	VRC 316 Schedule of Evaluations PART II: Group 3A/B and Group 4A/B											
**Visit Number**		*01	02	02A	03	04	05	06	06A	07	08	09	10	11
**Week of Study**		-12 to 0	W0	W1	W1	W2	W4	W16	W17	W17	W18	W20	W28	W40
**Day of Study**		-84 to 0	[1]D0	D1	D6	D14	D28	D112	D113	[6]D118	D126	D140	D196	D280
**Clinical**	Tube													
*VRC 500 Screening Consent		X												
VRC 316 AoU; Consent			X											
[2]Physical exam for eligibility, height /weight/ vitals at screening; vital signs and targeted exam (as needed) other visits.		X	X		X	X	X	X		X	X	X	X	X
Medical history targeted to eligibility at screening; then interim medical history		X	X		X	X	X	X		X	X	X	X	X
[3]Study Product Administration: **Part II, Group 3A/B**			**DNA A/Sing**					**HA-F A/Sing**						
[3]Study Product Administration: **Part II, Group 4A/B**			**HA-F A/Sing**					**HA-F A/Sing**						
Phone evaluation (clinic visit as needed)				X					X					
Begin diary card			X											
[4]Pregnancy test: urine or serum		X	X				X	X			X	X		X
[4]Pregnancy prevention counseling/ Reproductive Information Form		X	X				X	X			X	X		X
CBC with differential	EDTA	3	3			3	3	3				3		3
Iron		X					X					X		
Total bilirubin, AST, ALT, and ALP	GLT	4	4			4	4	4				4		4
Creatinine		X	X		4									
[5]HLA type	EDTA						20							
HIV (other tests, if needed)	SST	4												
**Research Samples**														
H. pylori ferritin antibody and Human ferritin antibody	SST	X					X					X		
Antibody assays. Serum storage	SST	32	32		32		32	32		32	32	32	32	32
Immunological assays (T cell and antibody responses). Multi-OMICS analysis. PBMCs and plasma storage	EDTA	80	80		80		80	80		[6]80	[7]120 or Apheresis	80	80	80
**Daily Volume (mL)**		123	119	0	116	7	139	119	0	112	152	119	112	119
**Max. Cumulative Volume (mL)**		123	242	242	358	365	504	623	623	735	887	1006	1118	1237

* VRC 500: Most screening evaluations must be no more than 84 days prior to Day 0 to be used for eligibility (pregnancy test from Day 0 must be used for eligibility). If clinical assessment on Day 0 suggests significant changes may have occurred since screening, then physical examination & laboratory studies done on Day 0 are used for eligibility.

[1] Day 0=day of enrollment and first vaccine injection. Day 0 evaluations prior to first injection are the baseline for assessing adverse events subsequently.

[2] Screening visit includes physical exam with vital signs. At other visits, physical examination is done if indicated by interim history or laboratory test results. Otherwise only blood pressure (BP), pulse, and temperature are required.

[3] Study Product Administration: Part II: Group 3A/B will receive DNA A/Sing HA-F A/Sing 4 mg IM at Day 0 and HA-F A/Sing 60 mcg IM at Week 16. Complete post vaccination evaluations (BP, pulse, temperature, respiration and injection site assessment) at 1 hour or longer after each study injection. Group 4A/B will receive HA-F A/Sing 60 mcg IM at Day 0 and at Week 16. Complete post vaccination evaluations (BP, pulse, temperature, respiration and injection site assessment) at 30 minutes or longer after each study injection.

[4] Negative pregnancy test results must be confirmed for women of reproductive potential prior to each study injection.

[5] HLA type blood sample is collected once at any time-point in the study and is shown as a Visit 05 evaluation for convenience; however, if HLA type is already available in the medical record, it does not need to be repeated. HLA type may also be obtained from a frozen sample.

[6] Visit 07: Two tubes of PBMCs collected in EDTA tubes will be sent to Building 40, while the remainder of the blood collected will be sent to VITL.

[7] If optional apheresis occurs, ONLY draw 32mL in SST (DO NOT draw 120 ml in EDTA tubes). For women of reproductive potential, pregnancy test must be negative within 72 hours prior to apheresis procedure.

Visit windows: Schedule Visits 02A through 06 with respect to Day 0; Schedule Visits 06A through 11 with respect to Visit 06. The following visit windows apply: Visits 02A and 06A (+1 day). Visit 03 and 07 (±1 day). Visits 04, 05 and 09 (±2 days). Visit 06 (±28 days). Visits 08 (±3 days). Visits 10 and 11 (±14 days).

APPENDIX III: ASSESSMENT OF RELATIONSHIP TO VACCINE AND GRADING SEVERITY OF ADVERSE EVENTS

Assessment of Relationship of an Adverse Event to Study Vaccine:

The relationship between an AE and the vaccine will be assessed by the investigator on the basis of his or her clinical judgment and the definitions below.

- **Definitely Related**. The AE and administration of study agent are related in time, and a direct association can be demonstrated.
- **Probably Related**. The AE and administration of study agent are reasonably related in time, and the AE is more likely explained by study agent than other causes.
- **Possibly Related**. The AE and administration of study agent are reasonably related in time, but the AE can be explained equally well by causes other than study agent.
- **Not Related**. The AE is clearly explained by another cause not related to the study product.

For purposes of preparing summary data reports in which AE attributions are simplified to “Related” or “Not Related”, in this protocol, the “Definitely, Probably and Possibly” attributions above will be mapped to the “Related” category, while the “Unlikely/Probably Not Related” and “Not Related” attributions above will be mapped to the “Not Related” category. The definitions that apply when these two attribution categories alone are used are as follows:

- **Related** – There is a reasonable possibility that the AE may be related to the study product(s).
- **Not Related** – There is not a reasonable possibility that the AE is related to the study product(s).

Grading the Severity of Adverse Events:

The FDA Guidance for Industry (September 2007): “Toxicity Grading Scale for Healthy Adult and Adolescent Volunteers Enrolled in Preventive Vaccine Clinical Trials” is the basis for the severity grading of AEs in this protocol. Several modifications were made to the table as follows:

- “Emergency room visit” is not automatically considered a life-threatening event; these words have been removed from any “Grade 4” definition where they appear in the table copied from the guidance document.
- Laboratory value shown as a “graded” value in the table that is within the institutional normal range will not be severity graded or recorded as an AE.
- Severity grading for hemoglobin decrease on the basis of the magnitude of decrease from baseline is not applicable at the Grade 1 level; only absolute hemoglobin will be used to define Grade 1.
- Severity grading for Grade 4 local reaction to injectable product (Erythema/Redness and Induration/Swelling) refer to necrosis or exfoliative dermatitis “requiring medical attention.”

When not otherwise specified, the following guidance will be used to assign a severity grade:

- **Grade 1 (Mild)**: No effect on activities of daily living

- **Grade 2 (Moderate)**: Some interference with activity not requiring medical intervention
- **Grade 3 (Severe)**: Prevents daily activity and requires medical intervention
- **Grade 4 (Life-threatening)**: Hospitalization; immediate medical intervention or therapy required to prevent death.
- **Grade 5 (Death)**: Death is assigned a Grade 5 severity. Only the single AE that is assessed as the primary cause of death should be assigned "Grade 5" severity.

Toxicity Grading Scale for Healthy Adult and Adolescent Volunteers Enrolled in Preventive Vaccine Clinical Trials
Modified from FDA Guidance - September 2007

A. Tables for Clinical Abnormalities

**Local Reaction to Injectable Product**	**Mild (Grade 1)**	**Moderate (Grade 2)**	**Severe (Grade 3)**	**Potentially Life Threatening (Grade 4)**
Pain	Does not interfere with activity	Repeated use of non-narcotic pain reliever > 24 hours or interferes with activity	Any use of narcotic pain reliever or prevents daily activity	Hospitalization
Tenderness	Mild discomfort to touch	Discomfort with movement	Significant discomfort at rest	Hospitalization
[1]Erythema/Redness	2.5 – 5 cm	5.1 – 10 cm	> 10 cm	Necrosis or exfoliative dermatitis requiring medical attention
[2] Induration/Swelling	2.5 – 5 cm and does not interfere with activity	5.1 – 10 cm or interferes with activity	> 10 cm or prevents daily activity	Necrosis requiring medical attention
**[3] Vital Signs**	**Mild (Grade 1)**	**Moderate (Grade 2)**	**Severe (Grade 3)**	**Potentially Life Threatening (Grade 4)**
[4] Fever (°C)<br>(°F)	38.0 – 38.4<br>100.4 – 101.1	38.5 – 38.9<br>101.2 – 102.0	39.0 – 40<br>102.1 – 104	> 40<br>> 104
Tachycardia - beats per minute	101 – 115	116 – 130	> 130	Hospitalization for arrhythmia

[5] Bradycardia - beats per Minute	50 – 54	45 – 49	< 45	Hospitalization for arrhythmia
Hypertension (systolic) - mm Hg	141 – 150	151 – 155	> 155	Hospitalization for malignant hypertension
Hypertension (diastolic) - mm Hg	91 – 95	96 – 100	> 100	Hospitalization for malignant hypertension
Hypotension (systolic) – mm Hg	85 – 89	80 – 84	< 80	Hospitalization for hypotensive shock
Respiratory Rate – breaths per minute	17 – 20	21 – 25	> 25	Intubation

1. In addition to grading the measured local reaction at the greatest single diameter, the measurement should be recorded as a continuous variable.
2. Induration/Swelling should be evaluated and graded using the functional scale as well as the actual measurement.
3. Subject should be at rest for all vital sign measurements.
4. Oral temperature; no recent hot or cold beverages or smoking.
5. When resting heart rate is between 60 – 100 beats per minute. Use clinical judgment when characterizing
   Bradycardia among some healthy subject populations, for example, conditioned athletes.

**Systemic (General)**	**Mild (Grade 1)**	**Moderate (Grade 2)**	**Severe (Grade 3)**	**Potentially Life Threatening (Grade 4)**
Nausea/vomiting	No interference with activity or 1 – 2 episodes/24 hours	Some interference with activity or > 2 episodes/24 hours	Prevents daily activity, requires outpatient IV hydration	Hospitalization for hypotensive shock
Diarrhea	2 – 3 loose stools or < 400 gms/24 hours	4 – 5 stools or 400 – 800 gms/24 hours	6 or more watery stools or > 800gms/24 hours or requires outpatient IV hydration	Hospitalization
Headache	No interference with activity	Repeated use of non-narcotic pain	Significant; any use of narcotic pain reliever or	Hospitalization

		reliever > 24 hours or some interference with activity	prevents daily activity	
Fatigue	No interference with activity	Some interference with activity	Significant; prevents daily activity	Hospitalization
Myalgia	No interference with activity	Some interference with activity	Significant; prevents daily activity	Hospitalization

**Systemic Illness**	**Mild (Grade 1)**	**Moderate (Grade 2)**	**Severe (Grade 3)**	**Potentially Life Threatening (Grade 4)**
Illness or clinical adverse event (as defined according to applicable regulations)	No interference with activity	Some interference with activity not requiring medical intervention	Prevents daily activity and requires medical intervention	Hospitalization

B. Tables for Laboratory Abnormalities

The laboratory values provided in the tables below serve as guidelines and are dependent upon the institutional normal parameters. Institutional normal reference ranges should be provided to demonstrate that they are appropriate.

**Serum ***	**Mild (Grade 1)**	**Moderate (Grade 2)**	**Severe (Grade 3)**	**Potentially Life Threatening (Grade 4) ****
Sodium – Hyponatremia mEq/L	132 – 134	130 – 131	125 – 129	< 125
Sodium – Hypernatremia mEq/L	144 – 145	146 – 147	148 – 150	> 150
Potassium – Hyperkalemia mEq/L	5.1 – 5.2	5.3 – 5.4	5.5 – 5.6	> 5.6
Potassium – Hypokalemia mEq/L	3.5 – 3.6	3.3 – 3.4	3.1 – 3.2	< 3.1
Glucose – Hypoglycemia mg/dL	65 – 69	55 – 64	45 – 54	< 45
Glucose – Hyperglycemia<br>Fasting – mg/dL<br>Random – mg/dL	 100 – 110<br>110 – 125	 111 – 125<br>126 – 200	 >125<br>>200	Insulin requirements or hyperosmolar coma
Blood Urea Nitrogen BUN mg/dL	23 – 26	27 – 31	> 31	Requires dialysis
Creatinine – mg/dL	1.5 – 1.7	1.8 – 2.0	2.1 – 2.5	> 2.5 or requires dialysis
Calcium – hypocalcemia mg/dL	8.0 – 8.4	7.5 – 7.9	7.0 – 7.4	< 7.0
Calcium – hypercalcemia mg/dL	10.5 – 11.0	11.1 – 11.5	11.6 – 12.0	> 12.0
Magnesium – hypomagnesemia mg/dL	1.3 – 1.5	1.1 – 1.2	0.9 – 1.0	< 0.9
Phosphorous – hypophosphatemia mg/dL	2.3 – 2.5	2.0 – 2.2	1.6 – 1.9	< 1.6
CPK – mg/dL	1.25 – 1.5 x ULN***	>1.5 – 3.0 x ULN	>3.0 –10 x ULN	> 10 x ULN
Albumin – Hypoalbuminemia g/dL	2.8 – 3.1	2.5 – 2.7	< 2.5	--
Total Protein – Hypoproteinemia g/dL	5.5 – 6.0	5.0 – 5.4	< 5.0	--
Alkaline phosphate – increase by factor	1.1 – 2.0 x ULN	2.1 – 3.0 x ULN	> 3.0 – 10 x ULN	> 10 x ULN

**Serum ***	**Mild (Grade 1)**	**Moderate (Grade 2)**	**Severe (Grade 3)**	**Potentially Life Threatening (Grade 4) ****
Liver Function Tests – ALT, AST increase by factor	1.1 – 2.5 x ULN	> 2.6 – 5.0 x ULN	> 5.1 – 10 x ULN	> 10 x ULN
Bilirubin – when accompanied by any increase in Liver Function Test increase by factor	1.1 – 1.25 x ULN	> 1.26 – 1.5 x ULN	> 1.51 – 1.75 x ULN	> 1.75 x ULN
Bilirubin – when Liver Function Test is normal; increase by factor	1.1 – 1.5 x ULN	1.6 – 2.0 x ULN	2.0 – 3.0 x ULN	> 3.0 x ULN
Cholesterol	201 – 210	211 – 225	> 226	---
Pancreatic enzymes – amylase, lipase	1.1 – 1.5 x ULN	1.6 – 2.0 x ULN	2.1 – 5.0 x ULN	> 5.0 x ULN

* The laboratory values provided in the tables serve as guidelines and are dependent upon institutional normal parameters. Institutional normal reference ranges should be provided to demonstrate that they are appropriate.

** The clinical signs or symptoms associated with laboratory abnormalities might result in characterization of the laboratory abnormalities as Potentially Life Threatening (Grade 4). For example, a low sodium value that falls within a Grade 3 parameter (125-129 mE/L) should be recorded as a Grade 4 hyponatremia event if the subject had a new seizure associated with the low sodium value.

***ULN" is the upper limit of the normal range.

Hematology *	Mild (Grade 1)	Moderate (Grade 2)	Severe (Grade 3)	Potentially Life Threatening (Grade 4)
Hemoglobin (Female) - gm/dL	11.0 – 12.0	9.5 – 10.9	8.0 – 9.4	< 8.0
Hemoglobin (Female) decrease from baseline value - gm/dL	not applicable	1.6 – 2.0	2.1 – 5.0	> 5.0
Hemoglobin (Male) - gm/dL	12.5 – 13.5	10.5 – 12.4	8.5 – 10.4	< 8.5
Hemoglobin (Male) decrease from baseline value – gm/dL	not applicable	1.6 – 2.0	2.1 – 5.0	> 5.0
WBC Increase - cell/mm^3	10,800 – 15,000	15,001 – 20,000	20,001 – 25, 000	> 25,000
WBC Decrease - cell/mm^3	2,500 – 3,500	1,500 – 2,499	1,000 – 1,499	< 1,000
Lymphocytes Decrease - cell/mm^3	750 – 1,000	500 – 749	250 – 499	< 250
Neutrophils Decrease - cell/mm^3	1,500 – 2,000	1,000 – 1,499	500 – 999	< 500
Eosinophils - cell/mm^3	650 – 1500	1501 - 5000	> 5000	Hypereosinophilic
Platelets Decreased - cell/mm^3	125,000 – 140,000	100,000 – 124,000	25,000 – 99,000	< 25,000
PT – increase by factor (prothrombin time)	1.10 x ULN**	> 1.11 – 1.20 x ULN	1.21 – 1.25 x ULN	> 1.25 ULN
PTT – increase by factor (partial thromboplastin time)	1.10 – 1.20 x ULN	1.21 – 1.4 x ULN	1.4 – 1.5 x ULN	> 1.5 x ULN
Fibrinogen increase - mg/dL	400 – 500	501 – 600	> 600	--
Fibrinogen decrease - mg/dL	150 – 200	125 – 149	100 – 124	< 100 or associated with gross bleeding or disseminated intravascular coagulation (DIC)

* The laboratory values provided in the tables serve as guidelines and are dependent upon institutional normal parameters. Institutional normal reference ranges should be provided to demonstrate that they are appropriate.

**ULN" is the upper limit of the normal range.

INSTITUTE: National Institute of Allergy and Infectious Diseases

STUDY NUMBER: 17-I-0110 PRINCIPAL INVESTIGATOR: Grace Chen, M.D., M.P.H.

STUDY TITLE: VRC 316: A Phase I Open-Label Clinical Trial to Evaluate Dose, Safety, Tolerability, and Immunogenicity of an Influenza HA Ferritin Vaccine, Alone or in Prime-Boost Regimens with an Influenza DNA Vaccine in Healthy Adults

Continuing Review Approved by the IRB on 04/23/18
Amendment Approved by the IRB on 11/13/18 (J) Date Posted to Web: 11/24/18
Standard Study Consent, Version 5.0

INTRODUCTION

We invite you to take part in a research study at the National Institutes of Health (NIH).

First, we want you to know that:

- Taking part in NIH research is entirely voluntary.
- You may choose not to take part, or you may withdraw from the study at any time. In either case, you will not lose any benefits to which you are otherwise entitled. However, to receive care at the NIH, you must be taking part in a study or be under evaluation for study participation.
- You may receive no benefit from taking part. The research may give us knowledge that may help people in the future.

Second, some people have personal, religious or ethical beliefs that may limit the kinds of medical or research treatments they would want to receive (such as blood transfusions). If you have such beliefs, please discuss them with your NIH doctors or research team before you agree to the study.

Now we will describe this research study. Before you decide to take part, please take as much time as you need to ask any questions and discuss this study with anyone at NIH, or with family, friends or your personal physician or other health professional.

PURPOSE OF THE STUDY

This is the first study in people of two experimental vaccines for prevention of H2 influenza (flu). "Experimental" means that the study vaccines have not been approved by the Food and Drug Administration (FDA). The FDA allows these vaccines to be used for research purposes only. These vaccines have never been given to humans before this study. We do not know if these vaccines work. The main purpose of this study is to see if the experimental vaccines are safe and if there are any side effects. We also want to study immune responses to these vaccines.

STUDY VACCINES

Vaccines are given to teach the body to prevent or fight an infection. In this study, we are testing two experimental vaccines that were developed by the Vaccine Research Center (VRC) at the NIH: VRC-FLUNPF081-00-VP, also called "HA-F A/Sing Vaccine" and VRC-FLUDNA082-00-VP, also called "DNA A/Sing Vaccine." These vaccines are intended to help the

body to make an immune response to H2 flu.

HA-F A/Sing Vaccine: Most vaccines are made of proteins that are injected into a muscle. Proteins are natural substances that the body uses as building blocks. This vaccine is made in the laboratory with two proteins: one protein from the H2 flu virus and one protein from a type of bacteria called *Helicobacter pylori* (*H. pylori*). These two proteins have been modified in the laboratory. When combined, they make a particle that looks like the outside of the H2 flu virus. The body's immune system may respond to this particle. There is no virus or bacteria in the vaccine.

DNA A/Sing Vaccine: DNA (deoxyribonucleic acid) is a molecule that serves as nature's code (instructions) for protein production in the body. The vaccine tested in this study is made from the DNA that is the code for an H2 flu virus protein. When you get the DNA vaccine, it tells your body to make a small amount of H2 protein. Your body may use this protein to build an immune response. There is no virus in the vaccine.

Similar DNA vaccines for other types of influenza were tested in many people. There were no serious side effects related to these DNA vaccines. The NIH has tested several DNA vaccines made by the VRC. These vaccines were found to be safe and well-tolerated in people.

This is the first study to give these two vaccines to humans. We do not know if the HA-F A/Sing or DNA A/Sing vaccine will protect you from the flu.

There is no virus or bacteria in the vaccines, so you cannot get an H2 flu infection or bacterial infection from these vaccines.

ELIGIBILITY

You are eligible to take part in this study because you have completed the screening process and are:

- 18-47 or 52-70 years old, unless you were born in 1966-1969
- In general good health without significant medical problems as determined at screening
- Willing to get the experimental vaccines
- Willing to donate blood samples for future research
- Willing to use birth control from at least 21 days prior to enrollment through the end of the study, if female and able to become pregnant

STUDY PLAN

About 50 people will take part in this study at the NIH Clinical Center in Bethesda, MD. You will be in the study for about 40 weeks (10 months) and get either 1 or 2 vaccinations. You will get the experimental vaccines by injections (shots) in the upper arm muscle. This is called an intramuscular "IM" injection. We will check you for any side effects from the vaccine.

We will tell you if we learn anything new during this study that might cause you to change your mind about staying in the study. At the end of the study, we will tell you when study results may be available and how to learn about them.

The study has two Parts:

Part I will test a lower dose (20 mcg) and higher dose (60 mcg) of the HA-F A/Sing vaccine. The study will start by enrolling people to get the lower dose in Group 1. If there are no safety concerns at this dose, we will start enrolling people to get the higher dose in Group 2. If there are no safety concerns at this dose, we will start enrolling people in Part II. We will use a needle and syringe to give you the HA-F A/Sing vaccine.

The table below shows the plan for Part I:

Part I				
**Group**	**Age**	**Subjects**	**Day 0**	**Week 16**
1	18-47 years	5	HA-F A/Sing, 20 mcg	
2	18-47 years	5	HA-F A/Sing, 60 mcg	HA-F A/Sing, 60 mcg
**Total**		**10**		

Part II will test the DNA A/Sing vaccine and the higher dose of the HA-F A/Sing vaccine. People will be grouped by age and randomly assigned (like pulling a number from a hat) to 1 of 2 groups (in Group 3 or Group 4). This is done so that researchers may compare the immune response when people were either exposed or not exposed to the H2 flu virus during their lifetime. Once enrolled, you will know which group you are in.

We will use a needle and syringe to give the HA-F A/Sing vaccine.

We will use a needle-free injection device called the PharmaJet Stratis® Needle Free Jet Injector (PharmaJet) to give the DNA A/Sing vaccine, it will be given as two injections at the visit, one in each upper arm. The needle-free injection device delivers the vaccine through the skin without the use of a needle. Instead, it uses high pressure to push the vaccine through your skin into the muscle. The needle-free injection device is "needleless," but may cause pain, some people have said that the sensation is similar of being snapped with a rubber band or as a mild punch in the arm. Needleless systems have been used to give vaccines and other medications since 1947. This system has been cleared by the FDA for giving vaccine injections into the muscle. Previous studies have shown that some people who get injections with PharmaJet have more local reactions at the injection site than people who get injections with a needle and syringe. These local reactions were mild.

The table below shows the plan for Part II:

Part II					
**Group**		**Age**	**Subjects**	**Day 0**	**Week 16**
3	A	18-47 years	10	DNA A/Sing, 4 mg; needle-free injector	HA-F A/Sing, 60 mcg; needle and syringe
	B	52-70 years	10		
4	A	18-47 years	10	HA-F A/Sing, 60 mcg; needle and syringe	HA-F A/Sing, 60 mcg; needle and syringe
	B	52-70 years	10		
	**Total**		**40**		

STUDY PROCEDURES

If you agree to take part in the study and are enrolled in:

- Group 1: you will have 1 vaccination visit. The HA-F A/Sing vaccine will be given as one injection at Day 0
- Group 2: you will have 2 vaccination visits. The HA-F A/Sing vaccine will be given as one injection at Day 0 and at Week 16.
- Group 3A/B: you will have 2 vaccination visits. The DNA A/Sing will be given as two injections at Day 0 and the HA-F A/Sing vaccine will be given as one injection Week 16.
- Group 4A/B: you will have 2 vaccination visits. The HA-F A/Sing vaccine will be given as one injection at Day 0 and at Week 16.

The study duration will last for 40 weeks (10 months) with about 8-10 clinic visits and 1 or 2 telephone contacts, depending on the group.

If you are a woman who could get pregnant, we will do a pregnancy test before each injection. The test must show that you are not pregnant to get the injection.

Each vaccination visit will take about 4-6 hours. Most follow-up clinic visits will take about 1 to 2 hours; the optional apheresis visit will take 2-4 hours.

If you are enrolled in Group 1, Group 2, or Group 3A/B, you will need to stay in the clinic for at least 1 hour after each vaccination. If you are enrolled in Group 4A/B, you will need to stay in the clinic for at least 30 minutes after each vaccination.

After each vaccination, the clinic staff will call you to check on you. Also, after each vaccination you will need to complete a diary card for 7 days after each injection. The purpose of the diary card is to record any symptoms that you may have. We will give you a thermometer to record your temperature even if you feel well. We will also give you a ruler to measure any skin changes at the injection site(s). You will get a password to a secure website to enter this data online. If you do not have access to a computer, you may use a paper diary card instead.

If you have any symptoms or feel unwell, you should tell a clinic nurse or doctor as soon as possible. You can reach the staff by phone 24 hours a day. If you have symptoms, you may be asked to come to the clinic for a checkup. It is very important that you follow the instructions from the clinic staff.

At each visit, we will check you for any health changes or problems. We will ask you how you are feeling and if you have taken any new medications. We will draw your blood at each visit, taking about 2 to 16 tubes of blood. We will tell you right away if any of your test results show a health problem. Some blood samples will be used for research only, to study your immune response to the vaccine. Results of these research tests are not used to check your health and will not be given to you.

Apheresis: After your final study injection we would like to collect your blood by a method called "Apheresis." This procedure is optional, and it is only being offered to subjects enrolled in Group 2, Group 3A/B, and Group 4A/B.

To be eligible for apheresis:

- You must not have an unstable heart as indicated by your medical history and test results
- You must not have blood pressure greater than 180/100
- You must not have a known blood clotting disorder
- You must not be pregnant or breast feeding
- You must not have a condition that the attending physician or the apheresis clinic staff considers a reason to not do an apheresis procedure.

Before apheresis, we will check your weight, pulse and blood pressure. We will ask questions about your general health and medical history. If you are a woman who could get pregnant, we will do a pregnancy test before the apheresis procedure. The test must show that you are not pregnant.

During procedure, you will lie on a recliner, couch, or hospital bed. The procedure requires placing a needle into a vein in one or both arms using a sterile method. The kits used to collect apheresis samples are sterile, single-use, disposable sets that are not in contact with any person's body fluids other than yours. No blood products are given to you during these procedures. The apheresis is done at the NIH Clinical Center, and a physician from the Department of Transfusion Medicine will be available in or near the apheresis donor area at all times.

In the apheresis procedure, blood is removed through a needle in the vein of one arm, spun in a machine that permits separation of the desired blood component (white blood cells or plasma), and then the remainder is returned through a needle in the other arm. Citrate, a medication to prevent blood from clotting, is added to the blood while in the machine to prevent it from clotting.

The purpose of these procedures is to allow the investigator to obtain and study a larger number of white blood cells or plasma than would otherwise be possible by simple blood drawing. The number of white blood cells or plasma collected is a small fraction of the total amount in your body. The body quickly replaces removed cells and plasma. Similar procedures are used daily in the Blood Bank of the Clinical Center and by other blood banks to obtain blood products from normal donors and as a form of therapy for certain diseases. However, we will not use your samples for transfusion or therapy. The procedure will take approximately 1-3 hours.

MONITORING OF THE STUDY

This study will be monitored by a group of physicians and scientists at NIH. This group will review the study information and will pay close attention to any reactions. If there are serious side effects, study injections may be delayed or canceled.

GENETIC TESTING

Some of the blood drawn from you during this study will be used for genetic tests. Some genetic tests are done in research studies to see if genetic differences in people cause different types of immune responses. Your blood sample

used in these genetic tests will not have your name on it and the results will not be in your medical record except for Human Leukocyte Antigen (HLA) typing results. These tests are not used to check your health and we will not tell you the results.

A special genetic test, called HLA typing, is done by the NIH Clinical Center medical laboratory. These results will be in your medical record but they will not be used to check your health. Any genetic testing, including HLA testing, is for research purposes only. Any genetic information collected or learned about you will be kept confidential. Medical records, including HLA test results are kept securely. We will not give any genetic information that is in your medical record to anyone without your permission.

STORED SAMPLES

We will collect blood samples from you during the study. We will keep these samples for future research indefinitely to learn more about flu virus, vaccines, the immune system, and other research questions. Results from research with your samples will not be in your medical record or reported to you.

Labeling of Stored Samples: Your stored samples will be labeled by a special code or number and not your personal information. Only the study team can link this code to you. Any identifying information about you (like name or date of birth) will be kept as confidential as allowable by law.

Risks of Stored Samples: There is a risk of unplanned release of information from your medical record. The chance that this information will be given to an unauthorized person without your permission is very small. Possible problems with the unplanned release of information include discrimination when applying for insurance or employment. Similar problems may occur if you give information about yourself or agree to have your medical record released.

Future studies: In the future, other investigators at NIH or outside of NIH may wish to study your stored samples. When your stored samples are shared, they will be marked with a code.

Your samples will not have any identifying information on them. Some information about you, like your gender, age, health history, or ethnicity may be shared with other researchers. Any future research studies using your samples will be conducted in a way that protects the rights and privacy of study participants.

Your stored samples will only be used for research and will not be sold. The research done with your materials may be used to develop new products in the future but you will not receive payment for such products.

Making your Choice: You cannot take part in this study if you do not want us to collect or store your blood samples. If you agree to take part in this study, you must also agree to let us keep any of your samples for future research. If you decide not to take part in this study, you may still take part in other studies at NIH.

HUMAN DATA SHARING

To advance science, it is helpful for researchers to share information they get from studying humans by putting it into shared scientific databases. Researchers can then study the information combined from many studies to learn even more about health and diseases.

If you agree to take part in this study, some of your data will be placed into one or more scientific databases. We will remove identifying information like your name, address, and birth date. The data may then be used for future research and shared broadly for research purposes.

Only researchers who are approved to access the database may be able to see and use your information. You will not get any direct benefits from future research that uses your data and information.

You may stop participating in this study at any time and withdraw permission for your individual data, specimens, and health information to be used for additional or future research. You may ask to have your research data destroyed. However, it may not be possible to withdraw or delete data once they have been shared with other researchers.

POSSIBLE STUDY RISKS

Possible **risks of injections:** Temporary stinging, pain, redness, soreness, itchiness, swelling, or bruising.

A needleless injector system called the PharmaJet Stratis® Needle Free Jet Injector (PharmaJet) will be used to give the DNA vaccine given in Group 3A/B. It uses pressure to push the vaccine into the arm.

There is a small chance the vaccine may spray on the arm and a small chance that the needle-free device could not work properly. As of June 26, 2017, in another research study called VRC 705 Part A, there have been 8 out of 426 (1.9%) injections that involved device malfunction. This includes the syringe breaking and study product spraying during administration. No harm to subjects has occurred when this happened. As a result, if you are enrolled in Groups 3A/B, we ask that you and the nurse wear safety glasses when PharmaJet is used for injection.

There is a very small chance of infection.

Vaccines given by needleless injector systems have been associated with mild skin lesions, and a small scab about 1-2 weeks after being given the vaccine.

Possible **risks of any vaccine:** Fever, chills, rash, aches and pains, nausea, headache, dizziness, and feeling tired and/or unwell. These types of reactions are usually greatest within the first 24 hours after vaccination and typically last 1 to 3 days. Over-the-counter medicine, like acetaminophen (Tylenol) or ibuprofen, may be used to help these symptoms.

Very rarely, a serious allergic reaction with symptoms like hives, trouble breathing, or sudden weakness may occur shortly after any vaccination. This is called "anaphylaxis" and may be life-threatening. While you are waiting in the clinic after the vaccination, we will monitor you for anaphylaxis. Treatment for anaphylaxis will be given right away if it occurs.

Possible **risks of the HA-F A/Sing vaccine**: The HA-F A/Sing vaccine has not been given to people before. It may have unknown risks. It has been tested in mice and rabbits. The vaccine did not cause any unusual side effects and met the safety criteria to be tested in humans.

Possible **risks of DNA A/Sing vaccine**: Temporary drop in white blood cell count, sore arm, skin rash or hives. Some people get a small red bump and then a scab for a few days where the shot is given. Possible side effects from intramuscular injections are stinging, arm discomfort, redness of the skin, or mild bruising.

***Possible* risks of blood drawing:** Pain, bleeding, bruising, feeling lightheaded, and fainting. Rarely, infection may occur at the site where the blood is taken.

***Possible* risks of apheresis**: Apheresis is generally safe and side effects are rare. Pain, bruising or discomfort at the needle placement site may occur. Sometimes apheresis causes a tingling sensation around the lips, nose and mouth, coolness all over and slight nausea. This can usually be relieved by slowing or temporarily stopping the apheresis or taking an antacid with calcium pill, like Tums®. Other possible side effects are anxiety, vomiting and lightheadedness. Temporary lowering of the blood pressure may develop. There is the rare possibility of infection, fainting or seizure. Very rarely a nerve problem at the needle placement site may occur. Also, very rarely, a machine malfunction may occur and result in the loss of about one unit (one pint) of blood.

There are theoretical risks from re-infusion of the blood after processing by the machine like infection or an adverse reaction to the blood components. However, this has not been seen in many thousands of volunteers who have undergone this or similar procedures to date. There may be other risks of apheresis that are unknown at this time.

***Unknown* safety risks**: There may be side effects from the study vaccines - even serious or life threatening ones- that we do not yet know about. Please tell the study staff about any side effect you think you are having. This is important for your safety.

***Possible* risks of genetic testing**: Unplanned release of information that could be used by insurers or employers to discriminate against you or your family; discovering a gene that suggests risk of disease for you or your family; discovering unknown family relationships.

***Possible* risks of data sharing:** Information in the shared databases could be linked back to you and used to discriminate against you or your family. State and federal laws provide some protections against genetic and pre-existing conditions discrimination.

***Possible* risks during pregnancy**: We do not know how the experimental vaccines may affect a fetus or nursing infant. Therefore, women who are able to become pregnant must have a negative pregnancy test before each injection and agree to use effective birth control beginning at least 21 days before the first injection until the end of the study. We will discuss effective methods of birth control with you. If you are pregnant or want to become pregnant in the next 40 weeks, you cannot participate.

You must tell the clinic staff right away if you become pregnant or think that you might be pregnant during the study. If you are pregnant, you will not get any more vaccinations. You will be asked to continue with follow-up visits so that we can check your health. We will ask you the outcome of the pregnancy.

***Possible* other risks**: We do not know if the study vaccine(s) will change how your body responds to flu virus infections in the future.

You may not donate blood at a blood bank while taking part in this study. You may not donate blood for one year after the last experimental vaccine injection.

POSSIBLE BENEFITS

This study is not designed to benefit you. We do not know if the vaccines will work. The study is not designed to protect you from flu. You and others may benefit in the future from the information that will be learned from the study. The study visits are used to check your health for research purposes, not to provide health care. However, we will tell you right away if any of your test results show a possible health problem.

COSTS TO YOU FOR PARTICIPATION

There are no costs to you for taking part in this study. We will not charge you or your insurance carrier for any health evaluations or services. You or your health insurance will have to pay for all costs for medical care that you get outside this study. It is possible that you may have some costs that are not covered by the study compensation we give you.

COMPENSATION TO YOU FOR PARTICIPATION

You will be compensated for your time and inconvenience by the NIH Clinical Research Volunteer Program. It is possible that you may have some expenses that are not covered by the compensation provided.

The compensation for is $275 for vaccination visit(s). You will get $25 total for the timely completion of all 7 days of an electronic diary. You will get $175 for each scheduled follow-up visit that includes a research blood draw. You will get $75 for all other visits that do not include research blood draws. You will get $250 for apheresis.

If you are enrolled in Group 1, the total compensation for completion of all study visits is about $1,525.

If you are enrolled in Groups 2-4 the total compensation for completion of all study visits is about $2,000 without apheresis and about $2,075 with apheresis.

Actual compensation is based on the number and type of study visits you complete. Your compensation may need to be reported to the Internal Revenue Service (IRS) as taxable income.

REASONS FOR STOPPING STUDY INJECTIONS

You may not get all of your planned study vaccinations. Reasons for this may include:

- You don't keep appointments or follow procedures.
- You get a serious illness that needs ongoing medical care.
- You have a serious side effect thought to be due to the study vaccine.
- You become pregnant.
- You need to get treatment with a medication that affects your immune system (such as a steroid like prednisone).
- The study is stopped by regulatory agencies, the study sponsor or study investigators. If this happens, we will tell you why.

If you agree to take part in this study, it is important for you to keep all of your appointments. Your participation in this

study is completely voluntary. You can choose to stop taking part in the study at any time. There is no penalty or loss of benefits choosing to leave the study.

If you get the first but not the second injection (for Groups 2, 3A/B, or 4A/B), we will ask you to continue with your planned follow-up visits until the end of the study. It is important that we continue to check your health even if you do not get a second injection.

ALTERNATIVES

This study is not designed to treat or prevent any disease. You may choose to not take part in this study. You may be eligible for other studies.

CONFLICT OF INTEREST

The NIH research staff is checked yearly for conflicts of interest. You may ask the research team for more information. This study may have investigators who are not NIH employees. Non-NIH investigators are expected to follow the principles of the Protocol Review Guide but are not required to report their personal financial holdings to the NIH.

The NIH, including some members of the VRC scientific staff, developed the investigational vaccines being used in this research study. The results of this study could play a role in whether the FDA will approve the vaccine for sale at some time in the future. If approved, the future sale of the vaccine could lead to payments to NIH and some NIH scientists. By U.S. law, government scientists are required to receive such payments for their inventions. You will not get any money from the development or sale of the product.

CLINICALTRIALS.GOV

A description of this clinical trial will be available on www.ClinicalTrials.gov. This website will not include information that can identify you. At most, the website will include a summary of the results. You can search this website at any time.

OTHER PERTINENT INFORMATION

1. Confidentiality. When results of an NIH research study are reported in medical journals or at scientific meetings, the people who take part are not named and identified. In most cases, the NIH will not release any information about your research involvement without your written permission. However, if you sign a release of information form, for example, for an insurance company, the NIH will give the insurance company information from your medical record. This information might affect (either favorably or unfavorably) the willingness of the insurance company to sell you insurance.

The Federal Privacy Act protects the confidentiality of your NIH medical records. However, you should know that the Act allows release of some information from your medical record without your permission, for example, if it is required by the Food and Drug Administration (FDA), members of Congress, law enforcement officials, or authorized hospital accreditation organizations.

2. Policy Regarding Research-Related Injuries. The Clinical Center will provide short-term medical care for any injury resulting from your participation in research here. In general, no long-term medical care or financial compensation for research-related injuries will be provided by the National Institutes of Health, the Clinical Center, or the Federal Government. However, you have the right to pursue legal remedy if you believe that your injury justifies such action.

3. Payments. The amount of payment to research volunteers is guided by the National Institutes of Health policies. In general, patients are not paid for taking part in research studies at the National Institutes of Health. Reimbursement of travel and subsistence will be offered consistent with NIH guidelines.

4. Problems or Questions. If you have any problems or questions about this study, or about your rights as a research participant, or about any research-related injury, contact the Principal Investigator, [redacted] or the Study Coordinator, [redacted].

You may also call the Clinical Center Patient Representative at [redacted].

5. Consent Document. Please keep a copy of this document in case you want to read it again.

COMPLETE APPROPRIATE ITEM(S) BELOW:

A. Adult Patient's Consent
I have read the explanation about this study and have been given the opportunity to discuss it and to ask questions. I hereby consent to take part in this study.

Signature of Adult Patient/Legal Representative ______ Date ______

Print Name ______

B. Parent's Permission for Minor Patient.
I have read the explanation about this study and have been given the opportunity to discuss it and to ask questions. I hereby give permission for my child to take part in this study.
(Attach NIH 2514-2, Minor's Assent, if applicable.)

Signature of Parent(s)/Guardian ______ Date ______

Print Name ______

C. Child's Verbal Assent (If Applicable)
The information in the above consent was described to my child and my child agrees to participate in the study.

Signature of Parent(s)/Guardian ______ Date ______ Print Name ______

THIS CONSENT DOCUMENT HAS BEEN APPROVED FOR USE
FROM APRIL 23, 2018 THROUGH APRIL 22, 2019.

Signature of Investigator ______ Date ______ Signature of Witness ______ Date ______

Print Name ______ Print Name ______

INSTITUTE: National Institute of Allergy and Infectious Diseases

STUDY NUMBER: 17-I-0110 PRINCIPAL INVESTIGATOR: Grace Chen, M.D., M.P.H.

STUDY TITLE: VRC 316: A Phase I Open-Label Clinical Trial to Evaluate Dose, Safety, Tolerability, and Immunogenicity of an Influenza HA Ferritin Vaccine, Alone or in Prime-Boost Regimens with an Influenza DNA Vaccine in Healthy Adults

Continuing Review Approved by the IRB on 04/23/18
Amendment Approved by the IRB on 11/13/18 (J) Date Posted to Web: 11/24/18
Supplementary Study Consent, Version 5.0

Supplementary consent for an extended genetic testing (optional)

You agreed to participate in VRC 316 study for testing of two investigational vaccines to prevent H2 influenza (flu) virus infection. This additional consent covers optional extended genetic testing that you may agree to. If you do not agree to this additional testing, you still can participate in the VRC 316 study.

There is a new type of genetic test that lets us look at the expressions of genes, called transcriptome sequencing. This test lets us look at the genes that are actively expressed at any given moment. However, it does not measure the amount of protein produced. Also, this new genetic test is still in development and researchers are working on understanding the data and how that data can be utilized in various clinical applications, such as in medicine to help prevent disease.

This test may take a long time to understand, and we may not have any news to give you about it. Since we are looking for genes that control infection or immune response, we will not report to you or your doctors things that we find that are not related to infection or immunity. However, if we find something in your DNA that we think is urgent to deal with because of your health, we will confirm the result and then tell you about it. We think this sort of problem will be rare.

Genetic Data Sharing

Following genetic testing for transcriptome sequencing, your sequence data will be shared in a controlled access public database, for other investigators to benefit from it. However, no personal, identifiable information will be shared in this process, as the shared results will be coded with no link back to you.

OTHER PERTINENT INFORMATION

1. Confidentiality. When results of an NIH research study are reported in medical journals or at scientific meetings, the people who take part are not named and identified. In most cases, the NIH will not release any information about your research involvement without your written permission. However, if you sign a release of information form, for example, for an insurance company, the NIH will give the insurance company information from your medical record. This information might affect (either favorably or unfavorably) the willingness of the insurance company to sell you insurance.

The Federal Privacy Act protects the confidentiality of your NIH medical records. However, you should know that the Act allows release of some information from your medical record without your permission, for example, if it is required by the Food and Drug Administration (FDA), members of Congress, law enforcement officials, or authorized hospital accreditation organizations.

2. Policy Regarding Research-Related Injuries. The Clinical Center will provide short-term medical care for any injury resulting from your participation in research here. In general, no long-term medical care or financial compensation for research-related injuries will be provided by the National Institutes of Health, the Clinical Center, or the Federal Government. However, you have the right to pursue legal remedy if you believe that your injury justifies such action.

3. Payments. The amount of payment to research volunteers is guided by the National Institutes of Health policies. In general, patients are not paid for taking part in research studies at the National Institutes of Health. Reimbursement of travel and subsistence will be offered consistent with NIH guidelines.

4. Problems or Questions. If you have any problems or questions about this study, or about your rights as a research participant, or about any research-related injury, contact the Principal Investigator [redacted] or the Study Coordinator, [redacted].
You may also call the Clinical Center Patient Representative at [redacted].

5. Consent Document. Please keep a copy of this document in case you want to read it again.

COMPLETE APPROPRIATE ITEM(S) BELOW:

A. Adult Patient's Consent
I have read the explanation about this study and have been given the opportunity to discuss it and to ask questions. I hereby consent to take part in this study.

Signature of Adult Patient/Legal Representative ______ Date ______

Print Name ______

B. Parent's Permission for Minor Patient.
I have read the explanation about this study and have been given the opportunity to discuss it and to ask questions. I hereby give permission for my child to take part in this study.
(Attach NIH 2514-2, Minor's Assent, if applicable.)

Signature of Parent(s)/Guardian ______ Date ______

Print Name ______

C. Child's Verbal Assent (If Applicable)
The information in the above consent was described to my child and my child agrees to participate in the study.

Signature of Parent(s)/Guardian ______ Date ______ Print Name ______

THIS CONSENT DOCUMENT HAS BEEN APPROVED FOR USE
FROM APRIL 23, 2018 THROUGH APRIL 22, 2019.

Signature of Investigator ______ Date ______ Signature of Witness ______ Date ______

Print Name ______ Print Name ______
